# New Cross-Talk Layer between Ultraconserved Non-Coding RNAs, MicroRNAs and Polycomb Protein YY1 in Bladder Cancer

**DOI:** 10.3390/genes7120127

**Published:** 2016-12-14

**Authors:** Sara Terreri, Montano Durso, Vincenza Colonna, Alessandra Romanelli, Daniela Terracciano, Matteo Ferro, Sisto Perdonà, Luigi Castaldo, Ferdinando Febbraio, Filomena de Nigris, Amelia Cimmino

**Affiliations:** 1 Institute of Genetics and Biophysics—CNR. Via P. Castellino, 111, 80131 Naples, Italy; sara.terreri@igb.cnr.it (S.T.); vincenza.colonna@igb.cnr.it (V.C.); 2 Bioker srl multimedica spa, via Brin, 49/65 80142 Naples, Italy; dursomontano@gmail.com; 3 Dipartimento di Farmacia, Università di Napoli “Federico II”, 80131 Naples, Italy; alessandra.romanelli@unina.it; 4 Department of Translational Medical Sciences, University of Naples “Federico II”, 80131 Naples, Italy; daniela.terracciano@unina.it; 5 Division of Urology, European Institute of Oncology, 20141 Milan, Italy; drmatteoferro@libero.it; 6 Division of Urology, IRCS National Tumor Institute, 80131 Naples, Italy; sisto.perdona@istitutotumori.na.it (S.P.); luigi.castaldo@gmail.it (L.C.); 7 Institute of Protein Biochemistry—CNR. Via P. Castellino, 111, 80131 Naples, Italy; f.febbraio@ibp.cnr.it; 8 Department of Biochemistry, Biophysic and General Pathology, University of Campania Luigi Vanvitelli, Via De Crecchio 7, 80138 Naples, Italy

**Keywords:** transcribed ultraconserved regions, microRNAs, bladder cancer, interaction network

## Abstract

MicroRNAs (miRNAs) are highly conserved elements in mammals, and exert key regulatory functions. Growing evidence shows that miRNAs can interact with another class of non-coding RNAs, so-called transcribed ultraconserved regions (T-UCRs), which take part in transcriptional, post-transcriptional and epigenetic regulation processes. We report here the interaction of miRNAs and T-UCRs as a network modulating the availability of these non-coding RNAs in bladder cancer cells. In our cell system, antagomiR-596 increased the expression of T-UCR 201+. Moreover, T-UCR 8+ silencing increased miR-596 expression, which in turn reduced total T-UCR 283+, showing that the perturbation of one element in this network changes the expression of other interactors. In addition, we identify the polycomb protein Yin Yang 1 (YY1) as mediator of binding between miR-596 and T-UCR 8+. These new findings describe for the first time a network between T-UCRs, miRNAs and YY1 protein, highlighting the existence of an additional layer of gene expression regulation.

## 1. Introduction

Long non-coding RNAs (lncRNAs) have been shown to play crucial roles in a variety of biological processes such as epigenetic control of gene expression, promoter-specific gene regulation [1,2,3], X-chromosome inactivation [4,5,6], imprinting [7,8,9,10,11], and maintenance of nuclear architecture [12,13,14]. They have also been implicated in many different diseases including cancer [15,16]. Recently, a new function of lncRNAs has been proposed, either as competitive endogenous RNAs (ceRNAs) for microRNAs (miRNAs) or naturally occurring miRNA sponges. ceRNA networks have been identified as key regulators of muscle differentiation [17] and involved in the phosphatase and tensin homolog (PTEN) tumor-suppressor pathway [18].

Transcribed ultraconserved regions (T-UCRs) are a particular class of lncRNAs. They include a group of 481 highly conserved sequences located in both intra- and intergenic regions. Little is known about their function, but their exact conservation in human, rat and mouse genome suggests an important regulatory role in gene expression [19]. Since T-UCR sequences are non-coding, they may influence gene expression by modulating miRNA activity or acting as antisense inhibitors of coding messenger RNAs (mRNAs) [20]. Most importantly, recent data suggest that T-UCRs are preferentially located in the cytoplasm, where they are able to establish interactions with other transcripts [21]. Experimental evidence supports an extensive targeting on T-UCRs by miRNAs, while other T-UCRs have significant antisense complementarity with miRNAs, which could lead to the formation of T-UCR/miRNA complexes by competing with endogenous target RNAs [22]. However, the full extent of the miRNA-dependent regulatory role of T-UCRs remains to be determined. Furthermore, as part of a regulatory loop, it seems that miRNAs can be involved in the transcriptional regulation of cancer-associated T-UCRs [21,22,23]. Consequently, aberrant T-UCR expression profiles can be used to differentiate cancer behaviors. Regulation of T-UCR expression has been found to occur via two main mechanisms: by interactions with miRNAs or by hypermethylation of CpG island promoters [24]. Accordingly, the transcription of T-UCRs located within protein-coding genes is associated with histone marks for active transcription [25,26].

Polycomb group (PcG) proteins are chromatin modifiers that have key roles in the silencing of developmental genes controlling the balance between differentiation and stem cell self-renewal. More recently, they have been found to have important functions in development and progression of several human cancers [27,28,29]. Growing evidence demonstrates that, apart from specific transcription factors that recruit PcG proteins, lncRNAs also recruit PcG proteins [30] to control gene expression by mediating changes in chromatin structure [31]. A recent study indicates that RNA transcribed from regulatory elements could sequester polycomb protein Yin Yang 1 (YY1) [31]. The constitutively active transcription factor YY1 is overexpressed in several cancers [32] and is associated with both DNA and RNA molecules [33,34]. The binding of YY1 to regulatory elements may maintain enhancers/promoters in active or silenced status depending on cellular context [35,36,37]. Our present work aims to verify whether T-UCRs can interact with miRNAs through an interconnected network involving YY1.

## 2. Materials and Methods

### 2.1. Identification of Binding Sites for MicroRNAs in T-UCR Sequences

T-UCR sequences were obtained from supplementary data by Bejerano et al. [19] and converted into reverse-complementary RNA using online software CAPRI from the Bioinformatics portal of CEINGE (CAPRI: a new web interface for sequence analysis programs) [38]. miRNA sequences were obtained from the online database miRBase release 19 (miRBase annotating high confidence microRNAs using deep sequencing data) [19]. We selected human miRNAs with “high confidence”, which presented a highly conserved level of identity through different mammalian species. We used RNAhybrid software (v 2.1, Bielefeld, Germany) available online at http://bibiserv.techfak.uni-bielefeld.de/rnahybrid/ [39,40] as target prediction tool to identify putative miRNA target sites in T-UCR sequences [39]. T-UCRs::miRNAs RNA duplex formation was evaluated in high stringency conditions, by using constraint of seed nucleotide matching 2–7 and a *p* value lower than 0.05 [40].

### 2.2. Cell Cultures, Constructs and Transfection

Human BlCa J28 cell lines (American Type Culture Collection) were cultured as a monolayer in Minimal Essential Medium supplemented with 10% foetal bovine serum. Cells were grown in a humidified incubator in a 5% carbon dioxide atmosphere at 37 °C.

For the transient silencing of T-UCR 8+, J82 cells were transfected with small interference RNAs (siRNAs) using HiPerFect transfection reagent (Qiagen, Hilden, Germany), according to the manufacturer’s instructions. siRNAs were designed using siDirect software [41] with input of the complete T-UCR 8+ sequence [38]. According to the scores, we selected three sequences designated as siRNA-1, siRNA-2, and siRNA-3 on the basis of their reduced capability to induce off-target effects correlated with thermodynamic stability of the seed-target duplex (<10 °C for siRNA-1 and siRNA-3 and <15 °C for siRNA-2) [38]. We performed all T-UCR 8+ silencing experiments using siRNA-3 [38].

For the transient silencing of YY1 in J82 cells, we used pBLOCK-iT RNAi entry Vector kit (Invitrogen, Carlsbad, California, USA) and the following oligonucleotide: TGCTGTATGAGGGCAAGCTATTGTTCGTTTTGGTTTTGGCCACTGACTGACTGACGAACAATAGCTTGCCCTCATAC or Scramble oligos. J82 cells were plated in six-well plates (0.18 × 10^6^ cells/well) and incubated for about 3 h at 37 °C. After incubation, cells were transfected with 1 μg of empty vector and 0.01, 0.2, 1, and 5 μg of shYY1 vector using Lipofectamine 2000 (Life Technology, Carlsbad, California, USA) transfection reagent, according to the manufacturer’s instructions. Cells were harvested 48 h after transfection and were subjected to RNA extraction and Western blotting analysis.

### 2.3. RNA Extraction, Reverse Transcription, and qRT-PCR

Total RNA was extracted from J82 cells using TRIzol reagent. The concentration of RNA was determined by 260/280 nm absorbance using a NanoDrop ND-1000 spectrophotometer (Thermo Scientific, Waltham, Massachusetts, USA), and the integrity of RNA was checked by gel electrophoresis. Total RNA (1 μg) was then reverse-transcribed using the QuantiTect Reverse Transcription Kit (Qiagen). Quantitative real time PCR (qRT-PCR) was performed using strand-specific primers for T-UCR analysis (Table S1). A miRCURY LNA Universal RT miR PCR kit (Exiqon Vedbaek, Denmark) was used for miR-596 and U6 (control) analysis according to the manufacturer’s instructions. RT-PCR analysis was performed using the iQ SYBR Green Supermix (BioRad, Hercules, California, USA) protocol with a CFX96 Real-Time PCR Detection System (BioRad) according to the manufacturer’s instructions. Small nuclear RNA U6 was used as reference for both T-UCRs and miRNAs. Table S1 lists primers used in this study for qRT-PCR. Each sample was analysed in triplicate. The 2^−Δ/Δ*C*t^ method was used for relative quantitation of gene expression, and results are expressed as fold change [42].

### 2.4. Western Blotting Assay

Total cellular proteins were size-fractionated on Bis-acrylamide 12% gels (Sigma-Aldrich Saint Louis, Missouri, USA) using Mini-Cell (Biorad) and transferred onto 0.45-μm nitrocellulose membranes using Immobilon-P (Millipore, Billerica, Massachusetts, USA) according to the manufacturer’s recommendations. For this experiment we used YY1 (C20, Santa Cruz Biotechnology, Santa Cruz, USA) as primary antibody. Signal quantification was performed using Amersham Hyperfilm ECL (GE Healthcare, Little Chalfont, UK) and normalized to loading control (anti-α actin, Sigma). A dilution of 1:5000 of anti-rabbit IgG (Amersham, GE Healthcare) was used as secondary antibody.

### 2.5. RNA-Chromatin Immunoprecipitation (RNA-ChIP)

J82 bladder cancer cells, (1 × 10^7^ cells), were fixed with 1% formaldehyde for 10 min at room temperature. Cells were washed extensively with phosphate-buffered saline (PBS), and the chromatin was sheared by sonication (Bioruptor, Diagenode Seraing, Ougrée, Belgium). The cross-linked RNA-protein complexes were immunoprecipitated with YY1 (C20, Santa Cruz Biotechnology) antibody at 4 °C overnight. Normal mouse IgG was used as negative controls. Immunocomplexes were pulled down through A/G plus resin (Santa Cruz Biotechnology) incubation and RNAs obtained from the decross-linking reaction were retro-transcribed and amplified by quantitative PCR (qPCR). Real time PCRs were performed with T-UCR 8+ and T-UCR 201+ primers (as shown in Table S1) and data were expressed as ΔCt of the housekeeping gene and fold change to non-immunoprecipitated samples. Finally, DNase (Sigma-Aldrich) was used at final concentration of 10 μM.

### 2.6. Magnetic Labelling and Isolation of Biotinylated Molecules (T-UCR Fishing)

Total RNA (300 μg) extracted from J82 cell line was incubated in an appropriate buffer with 100 pmol of 5′-biotinylated oligonucleotides T-UCR 8+, T-UCR 201+, T-UCR 128+ and one oligonucleotide scramble in miR-596 binding site overnight at 4 °C with rotation. After complex formation, 100 μL of μMACS Streptavidin MicroBeads (Miltenyi Biotec, Bergisch, Gladbach, Germany) were added and incubated for 30 min at 4 °C. μMACS columns were equilibrated with 100 μL of Equilibration buffer (for nucleic acid; supplied with the kit) and rinsed with the same buffer used for the binding reaction. Labelled complex was applied onto the top of the column matrix. Columns were washed with 4 × 100 μL of washing buffer (supplied with the kit) to remove non-specifically binding molecules. RNA labelled to biotinylated oligonucleotides was eluted with 150 μL of elution buffer (supplied with the kit) according to manufacturer’s instructions μMACSTM Streptavidin Kit).

### 2.7. Statistical Analysis

Results are reported as mean ± standard deviation. Statistical comparisons of all data were performed using the Student *t*-test with GraphPad Prism software (GraphPad Software, California, USA). Comparisons were considered statistically significant when *p* < 0.05.

## 3. Results

### 3.1. Bladder Specific T-UCRs Associate with miR-596

**Figure 1:**
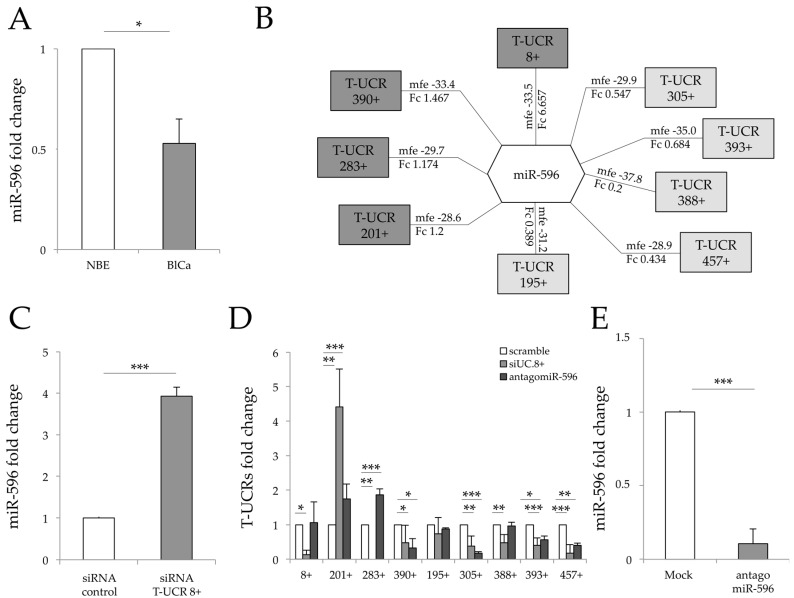
Transcribed ultraconserved regions (T-UCRs)::miR-596 interaction network and long non-coding RNA (lncRNA) expression levels following T-UCR 8+ and miR-596 downregulation. (**A**) miR-596 fold change comparison between bladder cancer (BlCa) and normal bladder epithelium (NBE) samples. We compared miR-596 expression in 24 BlCa patient samples and 17 NBE samples (clinical characteristics shown in Table S4); (**B**) Schematic representation of T-UCRs::miRNA-596 possible network. For each T-UCR, the minimum free energy (mfe) involved in miRNA-596 binding and the fold change (Fc) in BlCa samples compared to NBE are reported. Upregulated and downregulated T-UCRs in J82 bladder cancer cell lines are shown in dark grey and light grey respectively; (**C**) miR-596 expression change in T-UCR 8+-silenced cells vs scramble oligo-transfected cells; (**D**) Real-time quantification of the selected T-UCRs in T-UCR 8+ J82-silenced cells and in antagomiR-596 and scramble oligo-transfected cells; (**E**) Real-time dosage of miR-596 in antagomiR-596-transfected cells. Data are reported as fold change considering scrambles equal to 1 and as mean ± standard deviation (SD) of triplicate values. *p* values were obtained using the Student *t*-test for three independent samples. * *p* < 0.05, ** *p* < 0.01, *** *p* < 0.001 vs controls.

To predict the extent of miRNA-mediated regulation by T-UCRs in bladder carcinogenesis, we first scanned all sense and antisense T-UCRs (*n* = 962) previously selected from those described [19], and all miRNAs from the miRBase online database by using RNAhybrid software. We then selected pairs of T-UCRs and miRNAs by considering the minimum free energy in the biological context of bladder cancer (BlCa; Table S2). We focused on the 293 T-UCRs (≈ 60% of all T-UCRs analysed) that were differentially expressed at a statistically significant level (*p* < 0.05, *q* < 0.025) in BlCa tissues. From this analysis, we found that ≈ 11% of T-UCRs are interconnected by a single miRNA binding site, 83% of T-UCRs bind multiple miRNAs, and ≈ 6% have no binding sites for miRNAs (Tables S2 and S3). To facilitate our approach, we chose T-UCRs that share a binding site for the same single miRNA. We previously found miR-596 significantly downregulated in BlCa tissues compared to normal epithelium, as shown in Figure 1A, and established its function as tumour suppressor in BlCa tumorigenesis [38]. We therefore selected miRNA-596 as hypothetical binder of nine T-UCRs (T-UCR 8+, 195+, 201+, 283+, 305+, 388+, 390+, 393+ and 457+) (Figure 1B). T-UCR 8+, 201+, 283+ and 390+ resulted upregulated, while T-UCR 195+, 305+, 388+, 393+ and 457+ were downregulated in BlCa compared to normal bladder epithelium samples (Supplementary Table S2 and Figure 1B). To better understand the network of interactions between these nine T-UCRs and miR-596 (Figure 1B), we performed loss-of-function experiments on T-UCR 8+ since it is the most strongly expressed T-UCR in BlCa tissues (Figure 1C). In addition, T-UCR 8+ was previously found to contain miR-596-binding elements and to function as a competitive sponge for miR-596 binding [38]. We first investigated the impact of overexpression of miR-596 on the other T-UCRs involved in the network. As shown in Figure 1C,D, the silencing of T-UCR 8+ in J82 cells increased miR-596 expression by about fourfold (*p* < 0.001), completely abrogated T-UCR 283+ transcription (Figure 1D), and upregulated T-UCR 201+ expression by an almost fourfold change compared to control (*p* < 0.01) (Figure 1D). We then knocked down miR-596 (Figure 1E) by using an antagomiR that decreased the endogenous expression of miR-596 by about 10-fold (*p* < 0.001), resulting in increased expression of both T-UCR 201+ and T-UCR 283+ mRNAs by twofold (*p* < 0.001) compared to the control Figure 1D. These data suggest that T-UCR 283+ may be a target of miR-596 while T-UCR 201+ could function as a sponge in combination with T-UCR 8+ in order to repress miR-596 intracellular expression.

### 3.2. Identification of T-UCR 201+/miR-596 Interaction Using a Fishing Approach

**Figure 2:**
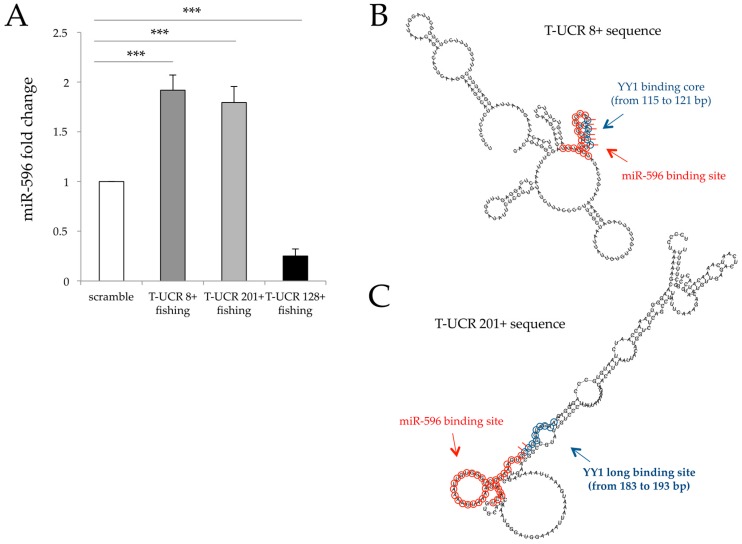
miR-596::T-UCR 8+/201+ binding, T-UCR 8+/201+ secondary structure and miR-596 and YY1 binding site. (**A**) Real-time PCRs and fold change of miR-596 pull down with peptide nucleic acid (PNA)/T-UCR 8+, (PNA)/T-UCR 201+ and (PNA)/T-UCR 128+ in J82 cancer cell line; (**B**) Predicted RNA secondary structure of T-UCR 8+ with miR-596 (circles in red) and YY1 (circles in blue) binding site. The short red lines indicate regions in which both miR-596 and YY1 bind T-UCR 8+ sequence; (**C**) Predicted RNA secondary structure of T-UCR 201+ with miR-596 (circles in red) and YY1 (circles in blue) binding site. The short red lines indicate regions in which both miR-596 and YY1 bind T-UCR 201+ sequence. Scramble value is considered equal to 1. Data are expressed as the mean ± SD of triplicate values. *p* values were obtained using the Student *t*-test for three independent samples. *** *p* < 0.001 vs. control.

In order to validate T-UCR network accuracy, we performed three RNA fishing experiments to demonstrate the effective interaction of these macromolecules. We identified a single-strand region of T-UCR 8+, T-UCR 128+ and T-UCR 201+, and designed specific antisense, complementary and biotinylated peptide nucleic acid (PNA) probes as baits (Table S1). T-UCR 8+-PNA was used as positive control while T-UCR 128+-PNA was used as negative control since T-UCR 128+ has no predicted binding site for any miRNAs. As reported in Figure 2A, real-time PCR amplification on pull-down RNAs indicated a significant enrichment of miR-596 (about 2-fold higher than scramble PNAs; *p* < 0.001) with both T-UCR 201+ and T-UCR 8+. Validation of the binding specificity of these two T-UCRs and miR-596 in vitro support the hypothesis of the existence of a complex mechanism of regulation.

### 3.3. YY1 and T-UCR Regulatory Circuit

**Figure 3:**
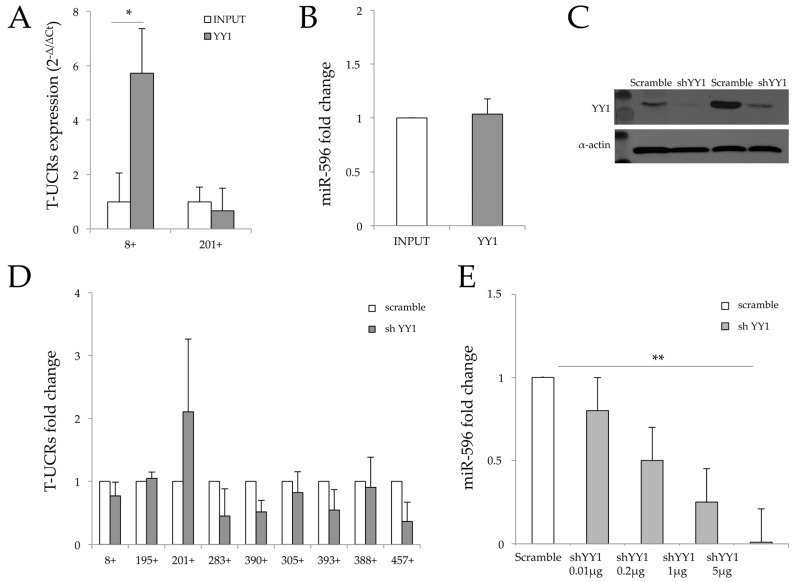
Role of polycomb YY1 in T-UCR network. (**A**) RNA-chromatin immunoprecipitation (RNA-ChIP) using YY1 and antibodies followed by real-time PCR (RT-PCR) with T-UCR 8+ and T-UCR 201+ set-primers on pull down materials. Data were expressed as ΔCt of the housekeeping gene U6 and fold change to non-immunoprecipitated samples INPUT (non-immunoprecipitated material) and reported as 2^−Δ/Δ*C*t^; (**B**) RNA-ChIP immunoprecipitation using YY1 antibodies followed by RT-PCR with miR-596 set-primers on pull down materials. Data were expressed as ΔCt of the housekeeping gene U6 and fold change to non-immunoprecipitated samples INPUT (non-immunoprecipitated material); (**C**) Total protein extracts from J82 cell lines transfected with pbloKit-shYY1 and pbloKit-scramble vectors analysed by Western blot using YY1 antibody. Actin was used as control to equally load the samples; (**D**) real time PCR (RT-PCR) of the selected T-UCRs, in shYY1-silenced J82 cells compared to empty vector-transfected cells. White boxes indicate cells transfected with pblockiT scramble vector, and grey boxes indicate cells transfected with pblockiT shYY1; (**E**) qRT-PCR of miR-596 in pblockiT-scramble-transfected cells (white box) and cells transfected with pblockiT shYY1 at different doses as indicated (grey boxes). Data are reported as fold change considering pblockiT-scramble equal to 1 and as mean ± SD of triplicate values. *p* values were obtained using the Student *t*-test for three independent samples. * *p* < 0.05, ** *p* < 0.01 vs. control.

Since YY1 proteins have already been reported to interact with multiple DNA and/or RNA sequences [34], we scanned T-UCR sequences for YY1 consensus binding sites with MatInspector Professional 8.0 software (http://www.genomatix.de) [43]. The program predicted one possible YY1 binding site in both T-UCR 8+ and T-UCR 201+ sequences, as shown in Figure 2B,C. To confirm these bindings, we performed an RNA chromatin immunoprecipitation (ChIP) assay. A significant enrichment of T-UCR 8+ was observed with YY1 antibody (sixfold higher) compared to input (Figure 3A). In contrast, YY1 was not able to co-immunoprecipitate either T-UCR 201+ or miR-596 (Figure 3A,B). To better understand the role of YY1 in miR-596::T-UCR 8+ crosstalk, we silenced YY1 (Figure 3C). Our results indicated that YY1 silencing did not significantly affect the amount of T-UCR 8+, T-UCR 201+ and the selected T-UCRs analysed (Figure 3D), while it reduced miR-596 availability in a dose-dependent manner (Figure 3E). These findings suggest that the occupancy of YY1 on T-UCR 8+ may change its conformation, inhibiting miR-596 binding, by acting as a competitor of sponge mechanism. In contrast, when YY1 is downregulated, miR-596 is free to bind T-UCR 8+.

## 4. Discussion

Differential expression of T-UCRs has been reported in BlCa tissues, although the mechanism remains unknown [44]. Here, we have computationally identified a network of nine T-UCRs (T-UCR 8+, 195+, 201+, 283+, 305+, 388+, 390+, 393+ and 457+), both up- and downregulated in BlCa tissues that share binding sites for miR-596. miR-596 is located at the short arm of chromosome 8, which is often affected by focal break points in cancer [45]. A large deletion involving miR-596 was found in urothelial carcinomas, supporting the hypothesis of its key role in carcinogenesis [38]. In vitro RNA fishing experiments validated the direct biding between miR-596, T-UCR 8+ and T-UCR 201+, thus reinforcing a possible regulatory role of T-UCRs on miRNAs. The formation of T-UCR::miRNA pairs may have different roles, either targeting T-UCRs or leading to the formation of sponges that trap miRNAs. We report here the interaction of miRNAs and T-UCRs as networks modulating the availability of these lncRNAs in BlCa cells. Specifically, when intracellular miR-596 levels were modulated using a specific antagomiR, in our cell system, we observed an increased expression of T-UCR 201+, showing that the perturbation of one element in this network changes the expression levels of other interactors. Moreover, in absence of T-UCR 8+, miR-596 is available to bind other T-UCRs, such as T-UCR 283+, and may regulate its expression. While one-to-one interactions between miRNAs and T-UCRs are described in literature, this is the first evidence of network interaction between these two classes of non-coding RNAs (ncRNAs), which warrant more comprehensive studies. In addition, we report that in this intricate network the polycomb protein YY1 affects the viability of miR-596. The polycomb group (PcG) of proteins has epigenetic regulatory functions [35] and is already described to regulate miRNAs [33] and recruit lncRNAs to strengthen their activity [46]. YY1 is the only polycomb protein family member having a sequence-specific element for binding of DNA, RNA and miRNAs with pleiotropic roles [33,34]. A well-characterized YY1-miRNA regulatory loop involves miR-200 and miR-15/16, implicated in vascular endothelial growth factor A (VEGFA) regulation in sarcoma [36] and drug resistance in leukaemia [47]. Based on our observations, we hypothesize that the binding of YY1 to T-UCR 8+ may alter its conformation, inhibiting the binding of miR-596. In agreement, dose-dependent shYY1 plasmids affect the available amount of miR-596, opening up the possibility of new and as yet unexplored regulatory mechanisms orchestrated by polycomb YY1.

## Supplementary Materials

The following supplementary materials can be found at http://www.mdpi.com/2073-4425/7/12/127/s1, Table S1: Primers used in the study, Table S2: Comparison of top-ranked transcribed ultraconserved regions (T-UCRs) in bladder cancer (BlCa) and normal bladder epithelium samples, Table S3: T-UCRs/miRNAs predicted single and multiple binding sites, Table S4: Clinical characteristics of BlCa patients.
