# Supplementary material for: New Cross-Talk Layer between Ultraconserved Non-Coding RNAs, MicroRNAs and Polycomb Protein YY1 in Bladder Cancer
Source: Genes (Basel). 2016 Dec 14;7(12):127. doi: 10.3390/genes7120127 (PMC5192503; doi:10.3390/genes7120127)
Supplement: Supplementary file 1 [file genes-07-00127-s001.docx]

Supplementary Materials: New Cross-Talk Layer between Ultraconserved Non-Coding RNAs, MicroRNAs and Polycomb Protein YY1 in Bladder Cancer

Sara Terreri, Montano Durso, Vincenza Colonna, Alessandra Romanelli, Daniela Terracciano, Matteo Ferro, Sisto Perdonà, Luigi Castaldo, Ferdinando Febbraio, Filomena de Nigris and Amelia Cimmino

**Table S1.** Primers used in the study.

| **Procedures** | **Primer Name** | **Sequence** |
| --- | --- | --- |
| RT-PCR | T-UCR 8+ | Fw-GGTCGCCATGGATATGACA |
|  |  | Rv-CACTGTGGCTTTAAACTCAGGA |
| RT-PCR | T-UCR 195+ | Fw-GGGGTTACTCCCAGACTGAA |
|  |  | Rv-TGTCCTGAGGGTCAGGCTTA |
| RT-PCR | T-UCR 201+ | Fw-GCCGAGTGGAATGTTTTGAT |
|  |  | Rv-CGGCCGTATTGTCCCTTATT |
| RT-PCR | T-UCR 283+ | Fw-GGCGCATAATTGGTTCTGTA |
|  |  | Rv-GATGCATCTTGAGTGGGTCA |
| RT-PCR | T-UCR 305+ | Fw-CTTAAAGCCTGGGCCAATTA |
|  |  | Rv-CAGCTGAGCATCTCCTCCTC |
| RT-PCR | T-UCR 388+ | Fw-CATGTGTTTGTGCCCGATAG |
|  |  | Rv-CATTCCACATGACCAAACCA |
| RT-PCR | T-UCR 390+ | Fw-TTGTAAATGGCCCTGATGTG |
|  |  | Rv-AGGCCATGTATGTTCCGTTC |
| RT-PCR | T-UCR 393+ | Fw-GTCTCCACCTCTGCCTTTGA |
|  |  | Rv-CTGCGCTTACTGCTCTGTTG |
| RT-PCR | T-UCR 457+ | Fw-GTCCATCTGGCAAAGCATCT |
|  |  | Rv-CAGGATTCTGGGAAGGTTGT |
| RT-PCR | U6 | Fw-CTCGCTTCGGCAGCACATAT |
|  |  | Rv-AACGCTTCACGAATTTGCGT |
| RT-PCR | Syn-hsa-miR-596 | AAGCCUGCCCGGCUCCUCGGG |
| T-UCR 8+ silencing | siRNA-anti T-UCR 8+ | Passenger-CAUAUGUGUGUGUAUAUAUCUdTdT |
|  |  | Guide-AUAUAUACACACACAUAUGUGdTdT |
|  | siRNA control | Passenger-UUCUUCGAACGUGUCACGUdTdT |
|  |  | Guide-ACGUGACACGUUCGGAGAAdTdT |
| miR-596 silencing | antagomiR-596 | CCCGAGGAGCCGGGCAGGCTT-R8 |
| Fishing | PNA T-UCR 8+ | Biotin-HAx-CTGAAAACAACACAATAA-NH2 |
|  | PNA T-UCR 201+ | Biotin-HAx-CGTGGGCCGTGGGCTGGG-NH2 |
|  | PNA T-UCR 128+ | Biotin-HAx-TAATAATATGGGATGACT-NH2 |
|  | PNA scramble | Biotin-HAx-ACATAACATAACGAACAA-NH2 |

**Table S2.** Comparison of top-ranked transcribed ultraconserved regions (T-UCRs) in bladder cancer (BlCa) and normal bladder epithelium samples * on the basis of both fold change and minimum free energy of predicted miRNAs binding.

| **T-UCRs** | **miRNA** | **mfe  (kcal/mol)** | **Fold  Change** | **T-UCRs** | **miRNA** | **mfe  (kcal/mol)** | **Fold  Change** |
| --- | --- | --- | --- | --- | --- | --- | --- |
| **1+** | hsa-miR-28-5p | −26.7 | 1.341 | **335+** | hsa-miR-554 | −30.1 | 1.347 |
| **1+** | hsa-miR-96-5p | −27.0 | 1.341 | **335+** | hsa-miR-619 | −28.7 | 1.347 |
| **1+** | hsa-miR-122-5p | −26.2 | 1.341 | **335+** | hsa-miR-621 | −26.1 | 1.347 |
| **1+** | hsa-miR-188-5p | −27.2 | 1.341 | **335+** | hsa-miR-637 | −29.7 | 1.347 |
| **1+** | hsa-miR-188-3p | −34.1 | 1.341 | **335+** | hsa-miR-638 | −30.1 | 1.347 |
| **1+** | hsa-miR-423-3p | −30.0 | 1.341 | **335+** | hsa-miR-644a | −25.2 | 1.347 |
| **1+** | hsa-miR-409-3p | −29.9 | 1.341 | **335+** | hsa-miR-663a | −29.0 | 1.347 |
| **1+** | hsa-miR-518c-5p | −28.9 | 1.341 | **335+** | hsa-miR-660-5p | −27.1 | 1.347 |
| **1+** | hsa-miR-572 | −27.0 | 1.341 | **335+** | hsa-miR-769-5p | −27.4 | 1.347 |
| **1+** | hsa-miR-612 | −35.3 | 1.341 | **335+** | hsa-miR-885-5p | −26.4 | 1.347 |
| **1+** | hsa-miR-639 | −29.0 | 1.341 | **335+** | hsa-miR-760 | −27.1 | 1.347 |
| **1+** | hsa-miR-708-5p | −28.4 | 1.341 | **335+** | hsa-miR-939-3p | −27.5 | 1.347 |
| **10+** | hsa-miR-423-5p | −30.9 | 0.656 | **335+** | hsa-miR-663b | −31.1 | 1.347 |
| **10+** | hsa-miR-765 | −27.4 | 0.656 | **340+** | hsa-miR-31-5p | −28.3 | 0.713 |
| **10+** | hsa-miR-877-5p | −30.6 | 0.656 | **340+** | hsa-miR-30c-2-3p | −28.2 | 0.713 |
| **10+** | hsa-miR-939-5p | −39.1 | 0.656 | **340+** | hsa-miR-214-5p | −27.9 | 0.713 |
| **102+** | hsa-miR-593-3p | −27.0 | 0.664 | **340+** | hsa-miR-125a-3p | −29.7 | 0.713 |
| **102+A** | hsa-miR-149-3p | −29.2 | 0.868 | **340+** | hsa-miR-30c-1-3p | −27.3 | 0.713 |
| **102+A** | hsa-miR-939-5p | −32.6 | 0.868 | **340+** | hsa-miR-433 | −28.4 | 0.713 |
| **106+A** | hsa-miR-193b-5p | −29.1 | 0.356 | **340+** | hsa-miR-762 | −31.1 | 0.713 |
| **111+** | hsa-miR-27a-5p | −28.3 | 0.768 | **340+** | hsa-miR-675-5p | −29.5 | 0.713 |
| **111+** | hsa-miR-197-5p | −29.4 | 0.768 | **340+** | hsa-miR-920 | −27.9 | 0.713 |
| **111+** | hsa-miR-198 | −28.1 | 0.768 | **340+** | hsa-miR-937-3p | −34.1 | 0.713 |
| **111+** | hsa-miR-217 | −28.1 | 0.768 | **340+** | hsa-miR-939-5p | −33.5 | 0.713 |
| **111+** | hsa-miR-125b-1-3p | −28.5 | 0.768 | **342+** | hsa-let-7f-1-3p | −25.9 | 0.392 |
| **111+** | hsa-miR-145-5p | −28.2 | 0.768 | **342+** | hsa-let-7i-3p | −28.0 | 0.392 |
| **111+** | hsa-miR-149-3p | −32.0 | 0.768 | **342+** | hsa-miR-375 | −27.3 | 0.392 |
| **111+** | hsa-miR-185-3p | −30.4 | 0.768 | **342+** | hsa-miR-339-3p | −30.5 | 0.392 |
| **111+** | hsa-miR-365a-5p | −30.7 | 0.768 | **342+** | hsa-miR-588 | −27.4 | 0.392 |
| **111+** | hsa-miR-365b-5p | −29.9 | 0.768 | **342+** | hsa-miR-615-3p | −29.7 | 0.392 |
| **111+** | hsa-miR-331-5p | −29.4 | 0.768 | **342+** | hsa-miR-298 | −28.5 | 0.392 |
| **111+** | hsa-miR-486-3p | −31.7 | 0.768 | **342+** | hsa-miR-942 | −27.5 | 0.392 |
| **111+** | hsa-miR-638 | −35.8 | 0.768 | **343+** | hsa-miR-28-3p | −28.6 | 1.200 |
| **111+** | hsa-miR-658 | −30.1 | 0.768 | **343+** | hsa-miR-216a-3p | −29.2 | 1.200 |
| **111+** | hsa-miR-671-5p | −31.9 | 0.768 | **343+** | hsa-miR-134 | −29.3 | 1.200 |
| **111+** | hsa-miR-877-3p | −31.0 | 0.768 | **343+** | hsa-miR-149-3p | −30.8 | 1.200 |
| **111+** | hsa-miR-665 | −30.5 | 0.768 | **343+** | hsa-miR-371a-5p | −28.7 | 1.200 |
| **122+A** | hsa-miR-766-3p | −27.6 | 0.793 | **344+A** | hsa-miR-632 | −28.4 | 1.111 |
| **122+A** | hsa-miR-665 | −35.0 | 0.793 | **344+A** | hsa-miR-636 | −29.1 | 1.111 |
| **122+A** | hsa-miR-873-3p | −27.8 | 0.793 | **344+A** | hsa-miR-661 | −33.6 | 1.111 |
| **122+A** | hsa-miR-921 | −28.2 | 0.793 | **344+A** | hsa-miR-663a | −39.4 | 1.111 |
| **122+A** | hsa-miR-499b-5p | −28.4 | 0.793 | **344+A** | hsa-miR-654-5p | −28.4 | 1.111 |
| **125+A** | hsa-miR-222-5p | −29.8 | 0.808 | **344+A** | hsa-miR-659-3p | −29.0 | 1.111 |
| **126+** | hsa-miR-204-3p | −27.1 | 0.827 | **344+A** | hsa-miR-744-5p | −29.0 | 1.111 |
| **126+** | hsa-miR-128 | −26.8 | 0.827 | **344+A** | hsa-miR-760 | −27.1 | 1.111 |
| **126+** | hsa-miR-449a | −26.8 | 0.827 | **344+A** | hsa-miR-937-5p | −29.0 | 1.111 |
| **126+** | hsa-miR-502-5p | −26.8 | 0.827 | **344+A** | hsa-miR-939-5p | −32.4 | 1.111 |
| **126+A** | hsa-miR-563 | −25.7 | 0.777 | **344+A** | hsa-miR-548au-3p | −27.0 | 1.111 |
| **126+A** | hsa-miR-615-3p | −28.8 | 0.777 | **345+A** | hsa-miR-93-3p | −27.7 | 0.720 |
| **126+A** | hsa-miR-659-3p | −28.6 | 0.777 | **345+A** | hsa-miR-197-5p | −34.0 | 0.720 |
| **129+** | hsa-miR-18a-3p | −31.6 | 1.481 | **345+A** | hsa-miR-143-5p | −29.2 | 0.720 |
| **129+** | hsa-miR-33a-5p | −25.6 | 1.481 | **345+A** | hsa-miR-378a-5p | −29.8 | 0.720 |
| **129+** | hsa-miR-93-3p | −28.2 | 1.481 | **345+A** | hsa-miR-608 | −45.1 | 0.720 |
| **129+** | hsa-miR-96-3p | −25.5 | 1.481 | **345+A** | hsa-miR-638 | −32.1 | 0.720 |
| **129+** | hsa-miR-218-5p | −25.5 | 1.481 | **345+A** | hsa-miR-769-3p | −33.9 | 0.720 |
| **129+** | hsa-miR-224-5p | −28.3 | 1.481 | **345+A** | hsa-miR-675-5p | −30.6 | 0.720 |
| **129+** | hsa-miR-149-5p | −27.6 | 1.481 | **345+A** | hsa-miR-541-3p | −31.7 | 0.720 |
| **129+** | hsa-miR-361-3p | −28.7 | 1.481 | **346+** | hsa-miR-27a-5p | −27.2 | 2.494 |
| **129+** | hsa-miR-367-5p | −29.4 | 1.481 | **346+** | hsa-miR-27a-3p | −27.6 | 2.494 |
| **129+** | hsa-miR-370 | −27.3 | 1.481 | **346+** | hsa-miR-92a-1-5p | −30.0 | 2.494 |
| **129+** | hsa-miR-324-3p | −32.9 | 1.481 | **346+** | hsa-miR-216a-3p | −27.8 | 2.494 |
| **129+** | hsa-miR-423-3p | −31.9 | 1.481 | **346+** | hsa-miR-218-1-3p | −27.2 | 2.494 |
| **129+** | hsa-miR-412 | −30.9 | 1.481 | **346+** | hsa-miR-27b-3p | −25.6 | 2.494 |
| **129+** | hsa-miR-193b-3p | −26.8 | 1.481 | **346+** | hsa-miR-128 | −27.9 | 2.494 |
| **129+** | hsa-miR-532-3p | −29.0 | 1.481 | **346+** | hsa-miR-149-3p | −32.5 | 2.494 |
| **129+** | hsa-miR-551a | −33.1 | 1.481 | **346+** | hsa-miR-185-3p | −30.0 | 2.494 |
| **129+** | hsa-miR-551b-3p | −25.8 | 1.481 | **346+** | hsa-miR-296-5p | −26.8 | 2.494 |
| **129+** | hsa-miR-573 | −26.6 | 1.481 | **346+** | hsa-miR-296-3p | −34.7 | 2.494 |
| **129+** | hsa-miR-33b-5p | −30.1 | 1.481 | **346+** | hsa-miR-330-5p | −29.9 | 2.494 |
| **129+** | hsa-miR-636 | −32.1 | 1.481 | **346+** | hsa-miR-326 | −28.6 | 2.494 |
| **129+** | hsa-miR-648 | −25.3 | 1.481 | **346+** | hsa-miR-431-5p | −28.3 | 2.494 |
| **129+** | hsa-miR-892a | −26.6 | 1.481 | **346+** | hsa-miR-433 | −32.6 | 2.494 |
| **129+** | hsa-miR-744-3p | −26.9 | 1.481 | **346+** | hsa-miR-557 | −27.8 | 2.494 |
| **13+A** | hsa-miR-25-5p | −33.1 | 0.628 | **346+** | hsa-miR-608 | −29.9 | 2.494 |
| **13+A** | hsa-miR-27a-5p | −30.5 | 0.628 | **346+** | hsa-miR-637 | −29.9 | 2.494 |
| **13+A** | hsa-miR-197-5p | −36.7 | 0.628 | **346+** | hsa-miR-639 | −36.0 | 2.494 |
| **13+A** | hsa-miR-211-5p | −29.7 | 0.628 | **346+** | hsa-miR-650 | −26.5 | 2.494 |
| **13+A** | hsa-miR-211-3p | −32.1 | 0.628 | **346+** | hsa-miR-762 | −33.3 | 2.494 |
| **13+A** | hsa-let-7g-3p | −26.7 | 0.628 | **346+** | hsa-miR-760 | −26.6 | 2.494 |
| **13+A** | hsa-let-7i-3p | −28.3 | 0.628 | **346+** | hsa-miR-939-5p | −31.3 | 2.494 |
| **13+A** | hsa-miR-23b-5p | −27.9 | 0.628 | **346+A** | hsa-miR-19b-1-5p | −26.1 | 2.494 |
| **13+A** | hsa-miR-125b-1-3p | −31.9 | 0.628 | **346+A** | hsa-miR-23a-5p | −29.4 | 2.494 |
| **13+A** | hsa-miR-138-5p | −33.7 | 0.628 | **346+A** | hsa-miR-27a-5p | −31.4 | 2.494 |
| **13+A** | hsa-miR-141-5p | −27.3 | 0.628 | **346+A** | hsa-miR-129-1-3p | −25.8 | 2.494 |
| **13+A** | hsa-miR-143-5p | −29.7 | 0.628 | **346+A** | hsa-miR-145-5p | −28.3 | 2.494 |
| **13+A** | hsa-miR-125a-3p | −27.7 | 0.628 | **346+A** | hsa-miR-127-5p | −30.3 | 2.494 |
| **13+A** | hsa-miR-146a-5p | −28.1 | 0.628 | **346+A** | hsa-miR-129-2-3p | −27.8 | 2.494 |
| **13+A** | hsa-miR-149-3p | −29.7 | 0.628 | **346+A** | hsa-miR-185-3p | −27.5 | 2.494 |
| **13+A** | hsa-miR-184 | −27.3 | 0.628 | **346+A** | hsa-miR-320a | −29.0 | 2.494 |
| **13+A** | hsa-miR-185-3p | −27.9 | 0.628 | **346+A** | hsa-miR-99b-3p | −28.0 | 2.494 |
| **13+A** | hsa-miR-188-5p | −27.7 | 0.628 | **346+A** | hsa-miR-381-5p | −27.0 | 2.494 |
| **13+A** | hsa-miR-296-5p | −35.2 | 0.628 | **346+A** | hsa-miR-328 | −32.2 | 2.494 |
| **13+A** | hsa-miR-296-3p | −28.6 | 0.628 | **346+A** | hsa-miR-342-5p | −26.9 | 2.494 |
| **13+A** | hsa-miR-370 | −30.4 | 0.628 | **346+A** | hsa-miR-326 | −28.4 | 2.494 |
| **13+A** | hsa-miR-331-5p | −30.5 | 0.628 | **346+A** | hsa-miR-423-5p | −28.5 | 2.494 |
| **13+A** | hsa-miR-324-5p | −28.3 | 0.628 | **346+A** | hsa-miR-193b-5p | −27.5 | 2.494 |
| **13+A** | hsa-miR-339-5p | −28.8 | 0.628 | **346+A** | hsa-miR-593-3p | −25.3 | 2.494 |
| **13+A** | hsa-miR-484 | −32.8 | 0.628 | **346+A** | hsa-miR-617 | −25.9 | 2.494 |
| **13+A** | hsa-miR-486-3p | −28.3 | 0.628 | **346+A** | hsa-miR-661 | −28.9 | 2.494 |
| **13+A** | hsa-miR-146b-5p | −28.8 | 0.628 | **346+A** | hsa-miR-663a | −33.3 | 2.494 |
| **13+A** | hsa-miR-146b-3p | −28.8 | 0.628 | **346+A** | hsa-miR-671-5p | −31.0 | 2.494 |
| **13+A** | hsa-miR-202-3p | −33.0 | 0.628 | **346+A** | hsa-miR-671-3p | −28.4 | 2.494 |
| **13+A** | hsa-miR-517a-3p | −29.7 | 0.628 | **346+A** | hsa-miR-320b | −27.3 | 2.494 |
| **13+A** | hsa-miR-517b-3p | −29.7 | 0.628 | **346+A** | hsa-miR-762 | −35.7 | 2.494 |
| **13+A** | hsa-miR-517c-3p | −26.9 | 0.628 | **346+A** | hsa-miR-939-5p | −31.7 | 2.494 |
| **13+A** | hsa-miR-503-3p | −27.7 | 0.628 | **346+A** | hsa-miR-663b | −31.4 | 2.494 |
| **13+A** | hsa-miR-92b-5p | −31.2 | 0.628 | **346+A** | hsa-miR-323b-5p | −34.5 | 2.494 |
| **13+A** | hsa-miR-564 | −26.2 | 0.628 | **349+** | hsa-miR-449c-3p | −31.7 | 1.400 |
| **13+A** | hsa-miR-572 | −34.9 | 0.628 | **349+** | hsa-miR-675-5p | −30.3 | 1.400 |
| **13+A** | hsa-miR-550a-5p | −29.4 | 0.628 | **349+** | hsa-miR-103b | −27.7 | 1.400 |
| **13+A** | hsa-miR-593-3p | −26.8 | 0.628 | **350+** | hsa-miR-103a-2-5p | −26.8 | 0.523 |
| **13+A** | hsa-miR-608 | −33.5 | 0.628 | **350+** | hsa-miR-346 | −31.6 | 0.523 |
| **13+A** | hsa-miR-612 | −33.8 | 0.628 | **350+** | hsa-miR-92b-5p | −31.7 | 0.523 |
| **13+A** | hsa-miR-615-3p | −29.8 | 0.628 | **350+** | hsa-miR-103b | −29.8 | 0.523 |
| **13+A** | hsa-miR-623 | −32.3 | 0.628 | **352+** | hsa-miR-15a-3p | −26.5 | 1.585 |
| **13+A** | hsa-miR-632 | −33.5 | 0.628 | **352+** | hsa-miR-532-5p | −26.4 | 1.585 |
| **13+A** | hsa-miR-635 | −26.8 | 0.628 | **354+A** | hsa-miR-221-3p | −28.4 | 0.266 |
| **13+A** | hsa-miR-636 | −28.3 | 0.628 | **354+A** | hsa-miR-222-3p | −28.9 | 0.266 |
| **13+A** | hsa-miR-642a-5p | −27.0 | 0.628 | **354+A** | hsa-miR-135a-3p | −31.2 | 0.266 |
| **13+A** | hsa-miR-663a | −37.7 | 0.628 | **354+A** | hsa-miR-365a-5p | −29.3 | 0.266 |
| **13+A** | hsa-miR-657 | −33.9 | 0.628 | **354+A** | hsa-miR-365b-5p | −31.3 | 0.266 |
| **13+A** | hsa-miR-658 | −34.3 | 0.628 | **354+A** | hsa-miR-339-5p | −30.8 | 0.266 |
| **13+A** | hsa-miR-659-3p | −39.0 | 0.628 | **354+A** | hsa-miR-423-3p | −29.1 | 0.266 |
| **13+A** | hsa-miR-550a-3-5p | −26.9 | 0.628 | **354+A** | hsa-miR-409-5p | −27.3 | 0.266 |
| **13+A** | hsa-miR-766-5p | −29.3 | 0.628 | **354+A** | hsa-miR-512-5p | −27.1 | 0.266 |
| **13+A** | hsa-miR-762 | −31.1 | 0.628 | **354+A** | hsa-miR-597 | −27.0 | 0.266 |
| **13+A** | hsa-miR-675-5p | −34.0 | 0.628 | **354+A** | hsa-miR-612 | −29.1 | 0.266 |
| **13+A** | hsa-miR-874 | −29.3 | 0.628 | **354+A** | hsa-miR-671-5p | −31.6 | 0.266 |
| **13+A** | hsa-miR-541-3p | −27.6 | 0.628 | **354+A** | hsa-miR-769-5p | −28.5 | 0.266 |
| **13+A** | hsa-miR-877-5p | −27.3 | 0.628 | **354+A** | hsa-miR-514b-3p | −28.0 | 0.266 |
| **13+A** | hsa-miR-760 | −32.9 | 0.628 | **356+** | hsa-miR-106a-3p | −26.0 | 0.630 |
| **13+A** | hsa-miR-933 | −29.9 | 0.628 | **356+** | hsa-miR-139-5p | −29.1 | 0.630 |
| **13+A** | hsa-miR-938 | −27.0 | 0.628 | **356+** | hsa-miR-214-5p | −29.1 | 0.630 |
| **13+A** | hsa-miR-939-5p | −36.0 | 0.628 | **356+** | hsa-miR-23b-5p | −26.7 | 0.630 |
| **13+A** | hsa-miR-940 | −29.8 | 0.628 | **356+** | hsa-miR-143-5p | −33.4 | 0.630 |
| **13+A** | hsa-miR-663b | −33.5 | 0.628 | **356+** | hsa-miR-639 | −31.2 | 0.630 |
| **13+A** | hsa-miR-718 | −32.5 | 0.628 | **358+** | hsa-miR-27a-5p | −28.0 | 0.859 |
| **13+A** | hsa-miR-550b-2-5p | −27.0 | 0.628 | **358+** | hsa-miR-210 | −27.6 | 0.859 |
| **13+A** | hsa-miR-378g | −27.8 | 0.628 | **358+** | hsa-miR-330-3p | −29.9 | 0.859 |
| **133+A** | hsa-miR-647 | −29.5 | 0.526 | **358+** | hsa-miR-557 | −27.7 | 0.859 |
| **134+** | hsa-miR-330-3p | −27.2 | 0.613 | **358+** | hsa-miR-885-3p | −28.3 | 0.859 |
| **134+** | hsa-miR-485-5p | −27.9 | 0.613 | **359+** | hsa-miR-330-3p | −31.0 | 0.721 |
| **134+** | hsa-miR-575 | −26.6 | 0.613 | **359+** | hsa-miR-92b-5p | −29.0 | 0.721 |
| **134+** | hsa-miR-548b-3p | −25.3 | 0.613 | **359+A** | hsa-miR-194-3p | −29.3 | 0.721 |
| **134+** | hsa-miR-744-5p | −29.3 | 0.613 | **36+A** | hsa-miR-296-3p | −29.1 | 0.867 |
| **136+** | hsa-miR-324-3p | −30.0 | 1.673 | **36+A** | hsa-miR-371a-5p | −26.6 | 0.867 |
| **136+** | hsa-miR-339-5p | −29.9 | 1.673 | **36+A** | hsa-miR-493-3p | −28.8 | 0.867 |
| **136+** | hsa-miR-502-5p | −27.3 | 1.673 | **36+A** | hsa-miR-578 | −25.9 | 0.867 |
| **136+** | hsa-miR-668 | −29.2 | 1.673 | **36+A** | hsa-miR-584-3p | −30.1 | 0.867 |
| **136+** | hsa-miR-675-5p | −38.2 | 1.673 | **36+A** | hsa-miR-593-3p | −26.7 | 0.867 |
| **137+A** | hsa-miR-625-5p | −27.9 | 0.731 | **36+A** | hsa-miR-650 | −28.8 | 0.867 |
| **137+A** | hsa-miR-661 | −30.9 | 0.731 | **360+** | hsa-miR-211-3p | −27.4 | 0.588 |
| **137+A** | hsa-miR-744-5p | −32.1 | 0.731 | **360+** | hsa-miR-885-3p | −30.5 | 0.588 |
| **138+** | hsa-miR-365a-5p | −29.9 | 0.465 | **362+A** | hsa-miR-23a-3p | −26.7 | 0.321 |
| **139+** | hsa-miR-187-5p | −33.1 | 1.500 | **362+A** | hsa-miR-107 | −27.9 | 0.321 |
| **139+** | hsa-miR-484 | −29.1 | 1.500 | **362+A** | hsa-miR-27b-3p | −26.2 | 0.321 |
| **139+** | hsa-miR-744-5p | −31.1 | 1.500 | **362+A** | hsa-miR-125b-1-3p | −28.6 | 0.321 |
| **14+** | hsa-miR-197-5p | −28.8 | 1.177 | **362+A** | hsa-miR-320a | −27.7 | 0.321 |
| **14+** | hsa-miR-204-3p | −28.0 | 1.177 | **362+A** | hsa-miR-324-3p | −28.3 | 0.321 |
| **14+** | hsa-miR-219-5p | −25.9 | 1.177 | **362+A** | hsa-miR-339-5p | −30.0 | 0.321 |
| **14+** | hsa-miR-125a-3p | −27.1 | 1.177 | **362+A** | hsa-miR-486-3p | −30.4 | 0.321 |
| **14+** | hsa-miR-365a-5p | −27.2 | 1.177 | **362+A** | hsa-miR-487a | −26.6 | 0.321 |
| **14+** | hsa-miR-371a-3p | −29.4 | 1.177 | **362+A** | hsa-miR-539-3p | −26.2 | 0.321 |
| **14+** | hsa-miR-372 | −26.5 | 1.177 | **362+A** | hsa-miR-650 | −30.9 | 0.321 |
| **14+** | hsa-miR-342-5p | −25.8 | 1.177 | **362+A** | hsa-miR-663a | −29.5 | 0.321 |
| **14+** | hsa-miR-424-5p | −25.2 | 1.177 | **362+A** | hsa-miR-320b | −28.4 | 0.321 |
| **14+** | hsa-miR-193b-5p | −27.8 | 1.177 | **362+A** | hsa-miR-937-5p | −26.7 | 0.321 |
| **14+** | hsa-miR-505-5p | −31.5 | 1.177 | **363+A** | hsa-miR-510 | −30.6 | 0.480 |
| **14+** | hsa-miR-558 | −24.6 | 1.177 | **363+A** | hsa-miR-608 | −29.9 | 0.480 |
| **14+** | hsa-miR-575 | −25.5 | 1.177 | **363+A** | hsa-miR-762 | −33.0 | 0.480 |
| **14+** | hsa-miR-593-3p | −25.0 | 1.177 | **363+A** | hsa-miR-935 | −31.2 | 0.480 |
| **14+** | hsa-miR-608 | −32.5 | 1.177 | **366+** | hsa-miR-148a-3p | −26.9 | 1.384 |
| **14+** | hsa-miR-33b-3p | −27.9 | 1.177 | **366+** | hsa-miR-381-5p | −29.2 | 1.384 |
| **14+** | hsa-miR-637 | −29.1 | 1.177 | **366+** | hsa-miR-517-5p | −31.4 | 1.384 |
| **14+** | hsa-miR-638 | −37.6 | 1.177 | **366+** | hsa-miR-516b-3p | −26.3 | 1.384 |
| **14+** | hsa-miR-647 | −26.0 | 1.177 | **366+** | hsa-miR-516a-3p | −26.3 | 1.384 |
| **14+** | hsa-miR-663a | −32.3 | 1.177 | **366+** | hsa-miR-557 | −28.9 | 1.384 |
| **14+** | hsa-miR-762 | −40.1 | 1.177 | **366+** | hsa-miR-770-5p | −29.7 | 1.384 |
| **14+** | hsa-miR-892a | −26.2 | 1.177 | **366+** | hsa-miR-744-5p | −27.5 | 1.384 |
| **14+** | hsa-miR-744-5p | −28.1 | 1.177 | **369+** | hsa-miR-212-5p | −27.0 | 2.484 |
| **14+** | hsa-miR-920 | −28.3 | 1.177 | **369+** | hsa-miR-34b-5p | −26.1 | 2.484 |
| **14+** | hsa-miR-921 | −28.1 | 1.177 | **369+** | hsa-miR-330-5p | −26.9 | 2.484 |
| **140+A** | hsa-miR-23a-5p | −27.3 | 0.637 | **369+** | hsa-miR-331-3p | −26.9 | 2.484 |
| **140+A** | hsa-miR-30b-3p | −28.7 | 0.637 | **369+** | hsa-miR-939-3p | −28.5 | 2.484 |
| **140+A** | hsa-miR-450b-3p | −25.4 | 0.637 | **369+** | hsa-miR-664a-5p | −27.3 | 2.484 |
| **140+A** | hsa-miR-760 | −36.8 | 0.637 | **371+A** | hsa-miR-365b-5p | −32.3 | 0.764 |
| **141+** | hsa-miR-132-3p | −28.1 | 0.750 | **371+A** | hsa-miR-342-5p | −27.3 | 0.764 |
| **141+** | hsa-miR-610 | −27.7 | 0.750 | **371+A** | hsa-miR-515-5p | −27.8 | 0.764 |
| **142+A** | hsa-miR-211-3p | −27.5 | 0.329 | **371+A** | hsa-miR-627 | −27.2 | 0.764 |
| **142+A** | hsa-miR-149-3p | −32.4 | 0.329 | **371+A** | hsa-miR-298 | −31.2 | 0.764 |
| **142+A** | hsa-miR-941 | −27.9 | 0.329 | **371+A** | hsa-miR-541-5p | −30.0 | 0.764 |
| **143+A** | hsa-miR-29b-2-5p | −27.6 | 0.606 | **371+A** | hsa-miR-873-5p | −30.8 | 0.764 |
| **143+A** | hsa-miR-129-5p | −28.4 | 0.606 | **372+** | hsa-let-7a-2-3p | −36.4 | 0.448 |
| **143+A** | hsa-miR-370 | −27.8 | 0.606 | **372+** | hsa-let-7e-3p | −35.7 | 0.448 |
| **143+A** | hsa-miR-326 | −27.2 | 0.606 | **372+** | hsa-miR-210 | −30.1 | 0.448 |
| **143+A** | hsa-miR-518c-5p | −28.7 | 0.606 | **372+** | hsa-miR-23b-5p | −30.4 | 0.448 |
| **143+A** | hsa-miR-508-5p | −27.5 | 0.606 | **372+** | hsa-miR-135a-5p | −26.4 | 0.448 |
| **143+A** | hsa-miR-612 | −30.6 | 0.606 | **372+** | hsa-miR-127-3p | −28.7 | 0.448 |
| **143+A** | hsa-miR-718 | −28.0 | 0.606 | **372+** | hsa-miR-195-5p | −26.1 | 0.448 |
| **144+** | hsa-miR-17-3p | −27.5 | 0.780 | **372+** | hsa-miR-135b-5p | −26.4 | 0.448 |
| **144+** | hsa-miR-18a-3p | −30.4 | 0.780 | **372+** | hsa-miR-486-5p | −30.1 | 0.448 |
| **144+** | hsa-miR-23a-5p | −28.9 | 0.780 | **372+** | hsa-miR-503-5p | −27.7 | 0.448 |
| **144+** | hsa-miR-92a-1-5p | −27.4 | 0.780 | **372+** | hsa-miR-612 | −32.3 | 0.448 |
| **144+** | hsa-miR-149-3p | −27.6 | 0.780 | **372+A** | hsa-miR-149-3p | −31.0 | 0.798 |
| **144+** | hsa-miR-150-3p | −29.4 | 0.780 | **372+A** | hsa-miR-622 | −27.8 | 0.798 |
| **144+** | hsa-miR-363-5p | −26.7 | 0.780 | **372+A** | hsa-miR-661 | −29.8 | 0.798 |
| **144+** | hsa-miR-365a-5p | −26.9 | 0.780 | **372+A** | hsa-miR-939-5p | −40.8 | 0.798 |
| **144+** | hsa-miR-365b-5p | −29.1 | 0.780 | **374+A** | hsa-miR-106a-3p | −25.3 | 0.481 |
| **144+** | hsa-miR-370 | −29.1 | 0.780 | **374+A** | hsa-miR-431-5p | −32.7 | 0.481 |
| **144+** | hsa-miR-375 | −26.9 | 0.780 | **374+A** | hsa-miR-510 | −26.6 | 0.481 |
| **144+** | hsa-miR-324-3p | −28.6 | 0.780 | **374+A** | hsa-miR-642a-5p | −26.2 | 0.481 |
| **144+** | hsa-miR-486-5p | −27.3 | 0.780 | **374+A** | hsa-miR-668 | −28.8 | 0.481 |
| **144+** | hsa-miR-511 | −28.8 | 0.780 | **377+** | hsa-miR-367-5p | −26.9 | 0.560 |
| **144+** | hsa-miR-432-5p | −27.4 | 0.780 | **377+** | hsa-miR-346 | −29.3 | 0.560 |
| **144+** | hsa-miR-525-5p | −25.9 | 0.780 | **377+** | hsa-miR-744-3p | −27.5 | 0.560 |
| **144+** | hsa-miR-632 | −26.1 | 0.780 | **377+** | hsa-miR-943 | −28.5 | 0.560 |
| **144+** | hsa-miR-636 | −31.9 | 0.780 | **377+A** | hsa-let-7d-5p | −25.8 | 0.744 |
| **144+** | hsa-miR-639 | −28.5 | 0.780 | **377+A** | hsa-let-7e-5p | −27.6 | 0.744 |
| **144+** | hsa-miR-765 | −27.1 | 0.780 | **377+A** | hsa-miR-92a-1-5p | −29.9 | 0.744 |
| **144+** | hsa-miR-675-5p | −28.4 | 0.780 | **377+A** | hsa-miR-107 | −28.7 | 0.744 |
| **144+** | hsa-miR-675-3p | −25.7 | 0.780 | **377+A** | hsa-miR-149-3p | −31.5 | 0.744 |
| **144+** | hsa-miR-877-5p | −25.9 | 0.780 | **377+A** | hsa-miR-337-5p | −26.2 | 0.744 |
| **145+A** | hsa-miR-92a-2-5p | −26.4 | 2.326 | **377+A** | hsa-miR-658 | −29.3 | 0.744 |
| **145+A** | hsa-miR-103a-3p | −29.3 | 2.326 | **377+A** | hsa-miR-765 | −26.7 | 0.744 |
| **145+A** | hsa-miR-107 | −29.3 | 2.326 | **377+A** | hsa-miR-885-3p | −32.4 | 0.744 |
| **145+A** | hsa-miR-197-5p | −32.8 | 2.326 | **378+A** | hsa-miR-15a-3p | −29.2 | 0.318 |
| **145+A** | hsa-miR-150-3p | −28.4 | 2.326 | **378+A** | hsa-miR-25-3p | −27.5 | 0.318 |
| **145+A** | hsa-miR-195-5p | −26.0 | 2.326 | **378+A** | hsa-miR-31-3p | −27.2 | 0.318 |
| **145+A** | hsa-miR-296-3p | −27.8 | 2.326 | **378+A** | hsa-miR-211-5p | −29.3 | 0.318 |
| **145+A** | hsa-miR-486-3p | −29.5 | 2.326 | **378+A** | hsa-miR-125a-5p | −28.3 | 0.318 |
| **145+A** | hsa-miR-376a-2-5p | −26.3 | 2.326 | **378+A** | hsa-miR-34c-3p | −27.3 | 0.318 |
| **145+A** | hsa-miR-564 | −28.2 | 2.326 | **378+A** | hsa-miR-296-3p | −30.7 | 0.318 |
| **145+A** | hsa-miR-593-5p | −35.3 | 2.326 | **378+A** | hsa-miR-330-5p | −28.0 | 0.318 |
| **145+A** | hsa-miR-612 | −29.2 | 2.326 | **378+A** | hsa-miR-326 | −28.5 | 0.318 |
| **145+A** | hsa-miR-646 | −28.7 | 2.326 | **378+A** | hsa-miR-331-3p | −37.1 | 0.318 |
| **145+A** | hsa-miR-650 | −27.5 | 2.326 | **378+A** | hsa-miR-491-5p | −29.8 | 0.318 |
| **145+A** | hsa-miR-663a | −29.2 | 2.326 | **378+A** | hsa-miR-639 | −32.2 | 0.318 |
| **145+A** | hsa-miR-671-5p | −29.5 | 2.326 | **378+A** | hsa-miR-659-3p | −33.1 | 0.318 |
| **145+A** | hsa-miR-449c-5p | −29.3 | 2.326 | **378+A** | hsa-miR-877-5p | −29.5 | 0.318 |
| **145+A** | hsa-miR-762 | −30.2 | 2.326 | **378+A** | hsa-miR-939-5p | −30.7 | 0.318 |
| **145+A** | hsa-miR-744-5p | −27.8 | 2.326 | **378+A** | hsa-miR-939-3p | −30.3 | 0.318 |
| **145+A** | hsa-miR-885-3p | −28.4 | 2.326 | **378+A** | hsa-miR-940 | −31.1 | 0.318 |
| **145+A** | hsa-miR-940 | −28.7 | 2.326 | **378+A** | hsa-miR-663b | −32.7 | 0.318 |
| **145+A** | hsa-miR-548q | −26.8 | 2.326 | **381+** | hsa-miR-15a-3p | −27.5 | 0.765 |
| **146+** | hsa-miR-363-3p | −26.6 | 0.671 | **381+** | hsa-miR-33a-5p | −28.5 | 0.765 |
| **146+** | hsa-miR-675-3p | −26.8 | 0.671 | **381+** | hsa-miR-138-5p | −28.5 | 0.765 |
| **149+** | hsa-miR-26b-3p | −28.4 | 0.603 | **381+** | hsa-miR-148b-3p | −28.3 | 0.765 |
| **149+** | hsa-miR-129-5p | −27.4 | 0.603 | **381+** | hsa-miR-608 | −29.5 | 0.765 |
| **149+** | hsa-miR-367-3p | −26.6 | 0.603 | **381+** | hsa-miR-33b-3p | −33.7 | 0.765 |
| **149+** | hsa-miR-339-5p | −32.0 | 0.603 | **381+** | hsa-miR-885-3p | −28.3 | 0.765 |
| **149+** | hsa-miR-511 | −25.2 | 0.603 | **381+** | hsa-miR-642b-5p | −26.7 | 0.765 |
| **149+** | hsa-miR-523-3p | −28.6 | 0.603 | **382+A** | hsa-miR-34a-5p | −27.1 | 0.679 |
| **149+** | hsa-miR-563 | −25.6 | 0.603 | **382+A** | hsa-miR-135a-3p | −29.2 | 0.679 |
| **149+** | hsa-miR-575 | −27.3 | 0.603 | **382+A** | hsa-miR-320a | −27.1 | 0.679 |
| **149+** | hsa-miR-767-5p | −26.9 | 0.603 | **382+A** | hsa-miR-365a-5p | −28.5 | 0.679 |
| **149+** | hsa-miR-874 | −29.2 | 0.603 | **382+A** | hsa-miR-330-3p | −30.3 | 0.679 |
| **149+** | hsa-miR-937-5p | −26.7 | 0.603 | **382+A** | hsa-miR-622 | −27.4 | 0.679 |
| **149+** | hsa-miR-892c-3p | −27.0 | 0.603 | **382+A** | hsa-miR-638 | −30.8 | 0.679 |
| **149+A** | hsa-miR-31-5p | −26.4 | 0.694 | **382+A** | hsa-miR-646 | −26.2 | 0.679 |
| **149+A** | hsa-miR-29b-1-5p | −26.7 | 0.694 | **382+A** | hsa-miR-320b | −27.1 | 0.679 |
| **149+A** | hsa-miR-34a-5p | −27.1 | 0.694 | **382+A** | hsa-miR-762 | −28.6 | 0.679 |
| **149+A** | hsa-miR-27b-5p | −26.1 | 0.694 | **382+A** | hsa-miR-939-5p | −29.4 | 0.679 |
| **149+A** | hsa-miR-125a-3p | −27.4 | 0.694 | **385+A** | hsa-miR-15a-3p | −26.6 | 0.760 |
| **149+A** | hsa-miR-149-3p | −28.7 | 0.694 | **388+A** | hsa-miR-15a-5p | −28.2 | 0.328 |
| **149+A** | hsa-miR-320a | −29.9 | 0.694 | **388+** | hsa-let-7d-3p | −28.3 | 0.2 |
| **149+A** | hsa-miR-422a | −29.3 | 0.694 | **388+** | hsa-miR-191-3p | −28.9 | 0.2 |
| **149+A** | hsa-miR-423-3p | −28.6 | 0.694 | **388+** | hsa-miR-185-3p | −29.2 | 0.2 |
| **149+A** | hsa-miR-449a | −27.1 | 0.694 | **388+** | hsa-miR-370 | −29.1 | 0.2 |
| **149+A** | hsa-miR-557 | −27.3 | 0.694 | **388+** | hsa-miR-342-5p | −28.4 | 0.2 |
| **149+A** | hsa-miR-659-3p | −32.3 | 0.694 | **388+** | hsa-miR-326 | −27.6 | 0.2 |
| **149+A** | hsa-miR-320b | −30.6 | 0.694 | **388+** | hsa-miR-331-5p | −28.8 | 0.2 |
| **149+A** | hsa-miR-320c | −26.9 | 0.694 | **388+** | hsa-miR-324-3p | −29.4 | 0.2 |
| **149+A** | hsa-miR-320d | −25.3 | 0.694 | **388+** | hsa-miR-346 | −30.6 | 0.2 |
| **149+A** | hsa-miR-378e | −25.6 | 0.694 | **388+** | hsa-miR-519c-5p | −32.1 | 0.2 |
| **150+** | hsa-miR-744-5p | −31.0 | 0.567 | **388+** | hsa-miR-520a-5p | −29.1 | 0.2 |
| **151+A** | hsa-miR-19b-1-5p | −26.8 | 0.487 | **388+** | hsa-miR-519b-5p | −32.1 | 0.2 |
| **151+A** | hsa-miR-105-5p | −27.1 | 0.487 | **388+** | hsa-miR-525-5p | −32.6 | 0.2 |
| **151+A** | hsa-miR-105-3p | −26.5 | 0.487 | **388+** | hsa-miR-523-5p | −32.1 | 0.2 |
| **151+A** | hsa-miR-370 | −27.3 | 0.487 | **388+** | hsa-miR-518f-5p | −28.5 | 0.2 |
| **151+A** | hsa-miR-431-5p | −29.4 | 0.487 | **388+** | hsa-miR-526a | −29.6 | 0.2 |
| **151+A** | hsa-miR-511 | −27.0 | 0.487 | **388+** | hsa-miR-520c-5p | −29.6 | 0.2 |
| **151+A** | hsa-miR-614 | −28.5 | 0.487 | **388+** | hsa-miR-518c-5p | −28.4 | 0.2 |
| **151+A** | hsa-miR-638 | −30.7 | 0.487 | **388+** | hsa-miR-516b-3p | −26.3 | 0.2 |
| **151+A** | hsa-miR-767-3p | −27.3 | 0.487 | **388+** | hsa-miR-518e-5p | −32.1 | 0.2 |
| **151+A** | hsa-miR-449c-5p | −30.7 | 0.487 | **388+** | hsa-miR-518d-5p | −29.6 | 0.2 |
| **151+A** | hsa-miR-378b | −28.8 | 0.487 | **388+** | hsa-miR-522-5p | −32.1 | 0.2 |
| **151+A** | hsa-miR-378e | −25.8 | 0.487 | **388+** | hsa-miR-519a-5p | −32.1 | 0.2 |
| **153+A** | hsa-miR-197-5p | −29.5 | 0.262 | **388+** | hsa-miR-516a-3p | −26.3 | 0.2 |
| **153+A** | hsa-miR-20b-3p | −31.2 | 0.262 | **388+** | hsa-miR-508-5p | −33.0 | 0.2 |
| **153+A** | hsa-miR-193b-5p | −31.2 | 0.262 | **388+** | hsa-miR-593-3p | −26.3 | 0.2 |
| **153+A** | hsa-miR-615-5p | −28.4 | 0.262 | **388+** | hsa-miR-596 | −37.8 | 0.2 |
| **153+A** | hsa-miR-767-5p | −28.3 | 0.262 | **388+** | hsa-miR-602 | −31.5 | 0.2 |
| **156+A** | hsa-miR-96-5p | −27.0 | 0.840 | **388+** | hsa-miR-613 | −27.6 | 0.2 |
| **157+A** | hsa-miR-15a-5p | −26.0 | 0.631 | **388+** | hsa-miR-615-5p | −29.4 | 0.2 |
| **157+A** | hsa-miR-16-5p | −27.8 | 0.631 | **388+** | hsa-miR-623 | −30.6 | 0.2 |
| **157+A** | hsa-miR-92a-3p | −31.0 | 0.631 | **388+** | hsa-miR-636 | −31.9 | 0.2 |
| **157+A** | hsa-miR-103a-3p | −27.4 | 0.631 | **388+** | hsa-miR-766-3p | −30.1 | 0.2 |
| **157+A** | hsa-miR-218-2-3p | −27.0 | 0.631 | **388+** | hsa-miR-874 | −31.4 | 0.2 |
| **157+A** | hsa-miR-143-5p | −27.2 | 0.631 | **388+** | hsa-miR-939-5p | −35.6 | 0.2 |
| **157+A** | hsa-miR-191-3p | −27.1 | 0.631 | **388+** | hsa-miR-942 | −27.0 | 0.2 |
| **157+A** | hsa-miR-136-3p | −26.6 | 0.631 | **388+** | hsa-miR-663b | −34.8 | 0.2 |
| **157+A** | hsa-miR-346 | −32.0 | 0.631 | **388+A** | hsa-miR-15b-5p | −29.8 | 0.328 |
| **157+A** | hsa-miR-486-3p | −27.0 | 0.631 | **388+A** | hsa-miR-143-5p | −29.0 | 0.328 |
| **157+A** | hsa-miR-497-5p | −27.1 | 0.631 | **388+A** | hsa-miR-150-3p | −32.3 | 0.328 |
| **157+A** | hsa-miR-92b-3p | −34.8 | 0.631 | **388+A** | hsa-miR-296-3p | −33.0 | 0.328 |
| **157+A** | hsa-miR-615-3p | −30.4 | 0.631 | **388+A** | hsa-miR-365a-5p | −34.3 | 0.328 |
| **157+A** | hsa-miR-661 | −30.4 | 0.631 | **388+A** | hsa-miR-365b-5p | −31.4 | 0.328 |
| **157+A** | hsa-miR-662 | −26.9 | 0.631 | **388+A** | hsa-miR-345-3p | −36.8 | 0.328 |
| **157+A** | hsa-miR-663a | −29.3 | 0.631 | **388+A** | hsa-miR-20b-3p | −28.4 | 0.328 |
| **157+A** | hsa-miR-1264 | −26.2 | 0.631 | **388+A** | hsa-miR-146b-3p | −30.5 | 0.328 |
| **157+A** | hsa-miR-769-5p | −27.1 | 0.631 | **388+A** | hsa-miR-497-5p | −28.5 | 0.328 |
| **157+A** | hsa-miR-885-3p | −27.0 | 0.631 | **388+A** | hsa-miR-509-5p | −27.1 | 0.328 |
| **157+A** | hsa-miR-939-3p | −29.1 | 0.631 | **388+A** | hsa-miR-593-5p | −29.8 | 0.328 |
| **158+A** | hsa-miR-26b-3p | −29.7 | 0.610 | **388+A** | hsa-miR-608 | −33.0 | 0.328 |
| **158+A** | hsa-miR-92a-3p | −26.7 | 0.610 | **388+A** | hsa-miR-637 | −34.1 | 0.328 |
| **158+A** | hsa-miR-194-3p | −29.2 | 0.610 | **388+A** | hsa-miR-638 | −36.3 | 0.328 |
| **158+A** | hsa-miR-219-2-3p | −29.5 | 0.610 | **388+A** | hsa-miR-654-5p | −30.5 | 0.328 |
| **158+A** | hsa-miR-323a-5p | −29.6 | 0.610 | **388+A** | hsa-miR-509-3-5p | −27.7 | 0.328 |
| **158+A** | hsa-miR-339-5p | −34.0 | 0.610 | **388+A** | hsa-miR-663b | −29.2 | 0.328 |
| **158+A** | hsa-miR-497-5p | −26.6 | 0.610 | **388+A** | hsa-miR-103b | −28.8 | 0.328 |
| **158+A** | hsa-miR-503-5p | −29.8 | 0.610 | **388+A** | hsa-miR-15a-5p | −28.2 | 0.328 |
| **158+A** | hsa-miR-509-5p | −25.9 | 0.610 | **388+A** | hsa-miR-92a-2-5p | −27.0 | 0.328 |
| **158+A** | hsa-miR-557 | −27.8 | 0.610 | **388+A** | hsa-miR-15b-5p | −29.8 | 0.328 |
| **158+A** | hsa-miR-612 | −29.0 | 0.610 | **388+A** | hsa-miR-143-5p | −29.0 | 0.328 |
| **158+A** | hsa-miR-646 | −25.6 | 0.610 | **388+A** | hsa-miR-150-3p | −32.3 | 0.328 |
| **158+A** | hsa-miR-661 | −29.1 | 0.610 | **388+A** | hsa-miR-296-3p | −33.0 | 0.328 |
| **158+A** | hsa-miR-421 | −26.2 | 0.610 | **388+A** | hsa-miR-365a-5p | −34.3 | 0.328 |
| **158+A** | hsa-miR-663b | −31.4 | 0.610 | **388+A** | hsa-miR-365b-5p | −31.4 | 0.328 |
| **158+A** | hsa-miR-378b | −27.4 | 0.610 | **388+A** | hsa-miR-345-3p | −36.8 | 0.328 |
| **158+A** | hsa-miR-378h | −28.5 | 0.610 | **388+A** | hsa-miR-20b-3p | −28.4 | 0.328 |
| **159+A** | hsa-miR-214-3p | −32.2 | 0.613 | **388+A** | hsa-miR-146b-3p | −30.5 | 0.328 |
| **159+A** | hsa-miR-615-5p | −33.6 | 0.613 | **388+A** | hsa-miR-497-5p | −28.5 | 0.328 |
| **159+A** | hsa-miR-622 | −28.8 | 0.613 | **388+A** | hsa-miR-509-5p | −27.1 | 0.328 |
| **159+A** | hsa-miR-638 | −38.4 | 0.613 | **389+** | hsa-miR-210 | −28.0 | 0.524 |
| **159+A** | hsa-miR-542-5p | −29.8 | 0.613 | **389+** | hsa-miR-222-5p | −28.0 | 0.524 |
| **159+A** | hsa-miR-760 | −29.0 | 0.613 | **389+** | hsa-miR-370 | −28.5 | 0.524 |
| **16+** | hsa-miR-204-5p | −26.3 | 0.379 | **389+** | hsa-miR-623 | −28.7 | 0.524 |
| **16+** | hsa-miR-185-3p | −27.5 | 0.379 | **389+** | hsa-miR-664a-5p | −28.3 | 0.524 |
| **16+** | hsa-miR-296-3p | −27.4 | 0.379 | **390+** | hsa-miR-25-3p | −27.5 | 1.467 |
| **16+** | hsa-miR-519c-5p | −26.9 | 0.379 | **390+** | hsa-miR-596 | −33.4 | 1.467 |
| **16+** | hsa-miR-519b-5p | −26.9 | 0.379 | **390+** | hsa-miR-622 | −25.9 | 1.467 |
| **16+** | hsa-miR-523-5p | −26.9 | 0.379 | **390+** | hsa-miR-660-3p | −25.8 | 1.467 |
| **16+** | hsa-miR-518e-5p | −26.9 | 0.379 | **390+** | hsa-miR-939-5p | −36.7 | 1.467 |
| **16+** | hsa-miR-522-5p | −26.9 | 0.379 | **392+A** | hsa-miR-27a-5p | −28.2 | 0.508 |
| **16+** | hsa-miR-519a-5p | −26.9 | 0.379 | **392+A** | hsa-miR-197-5p | −29.4 | 0.508 |
| **16+** | hsa-miR-623 | −28.3 | 0.379 | **392+A** | hsa-miR-223-3p | −27.6 | 0.508 |
| **16+** | hsa-miR-708-3p | −26.4 | 0.379 | **392+A** | hsa-miR-125a-3p | −28.8 | 0.508 |
| **160+** | hsa-miR-204-3p | −29.8 | 2.156 | **392+A** | hsa-miR-149-3p | −32.4 | 0.508 |
| **160+** | hsa-miR-214-5p | −27.9 | 2.156 | **392+A** | hsa-miR-557 | −29.0 | 0.508 |
| **160+** | hsa-miR-346 | −37.4 | 2.156 | **392+A** | hsa-miR-593-3p | −26.4 | 0.508 |
| **160+** | hsa-miR-572 | −30.6 | 2.156 | **392+A** | hsa-miR-650 | −27.5 | 0.508 |
| **160+** | hsa-miR-610 | −32.7 | 2.156 | **393+** | hsa-miR-92a-1-5p | −29.1 | 0.684 |
| **160+** | hsa-miR-623 | −30.6 | 2.156 | **393+** | hsa-miR-197-5p | −32.6 | 0.684 |
| **160+** | hsa-miR-632 | −26.5 | 2.156 | **393+** | hsa-miR-181a-2-3p | −29.4 | 0.684 |
| **160+** | hsa-miR-663b | −36.5 | 2.156 | **393+** | hsa-miR-211-3p | −27.6 | 0.684 |
| **162+** | hsa-miR-15a-3p | −27.6 | 0.584 | **393+** | hsa-miR-149-5p | −34.4 | 0.684 |
| **162+** | hsa-miR-197-5p | −32.2 | 0.584 | **393+** | hsa-miR-412 | −29.3 | 0.684 |
| **162+** | hsa-miR-34a-5p | −28.1 | 0.584 | **393+** | hsa-miR-511 | −26.8 | 0.684 |
| **162+** | hsa-miR-30b-3p | −26.1 | 0.584 | **393+** | hsa-miR-193b-3p | −36.5 | 0.684 |
| **162+** | hsa-miR-149-3p | −29.1 | 0.584 | **393+** | hsa-miR-551a | −28.1 | 0.684 |
| **162+** | hsa-miR-185-5p | −26.6 | 0.584 | **393+** | hsa-miR-596 | −35.0 | 0.684 |
| **162+** | hsa-miR-34b-5p | −26.8 | 0.584 | **393+** | hsa-miR-608 | −33.4 | 0.684 |
| **162+** | hsa-miR-431-5p | −28.7 | 0.584 | **393+** | hsa-miR-638 | −36.5 | 0.684 |
| **162+** | hsa-miR-193b-5p | −28.5 | 0.584 | **393+** | hsa-miR-671-5p | −30.8 | 0.684 |
| **162+** | hsa-miR-504 | −29.2 | 0.584 | **393+** | hsa-miR-671-3p | −32.1 | 0.684 |
| **162+** | hsa-miR-612 | −30.9 | 0.584 | **393+** | hsa-miR-675-5p | −30.8 | 0.684 |
| **162+** | hsa-miR-33b-3p | −35.0 | 0.584 | **393+** | hsa-miR-888-3p | −30.9 | 0.684 |
| **162+** | hsa-miR-663a | −31.9 | 0.584 | **393+** | hsa-miR-892b | −31.2 | 0.684 |
| **162+** | hsa-miR-449b-5p | −27.6 | 0.584 | **393+** | hsa-miR-885-3p | −30.5 | 0.684 |
| **162+** | hsa-miR-668 | −27.9 | 0.584 | **393+** | hsa-miR-877-5p | −28.8 | 0.684 |
| **162+** | hsa-miR-449c-5p | −30.3 | 0.584 | **393+** | hsa-miR-920 | −26.9 | 0.684 |
| **162+** | hsa-miR-541-3p | −27.8 | 0.584 | **393+** | hsa-miR-514b-3p | −28.2 | 0.684 |
| **162+** | hsa-miR-744-5p | −27.2 | 0.584 | **393+** | hsa-miR-892c-3p | −28.2 | 0.684 |
| **162+** | hsa-miR-939-5p | −30.6 | 0.584 | **396+** | hsa-let-7a-2-3p | −26.4 | 1.239 |
| **162+A** | hsa-miR-139-3p | −31.7 | 0.599 | **396+** | hsa-miR-19a-5p | −28.9 | 1.239 |
| **162+A** | hsa-miR-149-3p | −30.3 | 0.599 | **396+** | hsa-miR-19b-1-5p | −27.1 | 1.239 |
| **162+A** | hsa-miR-365b-5p | −29.1 | 0.599 | **396+** | hsa-miR-191-3p | −29.2 | 1.239 |
| **162+A** | hsa-miR-370 | −29.1 | 0.599 | **396+** | hsa-miR-557 | −30.1 | 1.239 |
| **162+A** | hsa-miR-326 | −26.9 | 0.599 | **396+** | hsa-miR-620 | −24.7 | 1.239 |
| **162+A** | hsa-miR-146b-3p | −30.9 | 0.599 | **396+** | hsa-miR-758-5p | −26.8 | 1.239 |
| **162+A** | hsa-miR-572 | −27.0 | 0.599 | **396+** | hsa-miR-769-5p | −27.3 | 1.239 |
| **162+A** | hsa-miR-629-3p | −27.6 | 0.599 | **397+A** | hsa-miR-214-5p | −28.2 | 0.596 |
| **162+A** | hsa-miR-758-5p | −28.3 | 0.599 | **397+A** | hsa-miR-145-5p | −28.0 | 0.596 |
| **162+A** | hsa-miR-550a-3-5p | −25.7 | 0.599 | **397+A** | hsa-miR-296-3p | −29.4 | 0.596 |
| **162+A** | hsa-miR-769-3p | −28.1 | 0.599 | **397+A** | hsa-miR-331-3p | −29.1 | 0.596 |
| **162+A** | hsa-miR-762 | −35.2 | 0.599 | **397+A** | hsa-miR-324-5p | −30.4 | 0.596 |
| **162+A** | hsa-miR-885-3p | −30.8 | 0.599 | **397+A** | hsa-miR-346 | −29.8 | 0.596 |
| **162+A** | hsa-miR-873-3p | −28.1 | 0.599 | **397+A** | hsa-miR-424-5p | −26.7 | 0.596 |
| **162+A** | hsa-miR-939-5p | −35.0 | 0.599 | **397+A** | hsa-miR-490-5p | −28.6 | 0.596 |
| **162+A** | hsa-miR-939-3p | −32.3 | 0.599 | **397+A** | hsa-miR-491-5p | −28.6 | 0.596 |
| **162+A** | hsa-miR-550b-2-5p | −27.0 | 0.599 | **397+A** | hsa-miR-500a-3p | −27.6 | 0.596 |
| **163+** | hsa-miR-615-5p | −36.4 | 0.512 | **397+A** | hsa-miR-502-3p | −27.6 | 0.596 |
| **163+** | hsa-miR-658 | −33.2 | 0.512 | **397+A** | hsa-miR-550a-5p | −28.6 | 0.596 |
| **163+** | hsa-miR-762 | −31.0 | 0.512 | **397+A** | hsa-miR-610 | −26.7 | 0.596 |
| **166+** | hsa-miR-516a-5p | −28.4 | 0.817 | **397+A** | hsa-miR-615-5p | −29.9 | 0.596 |
| **166+** | hsa-miR-671-5p | −31.2 | 0.817 | **397+A** | hsa-miR-637 | −31.0 | 0.596 |
| **168+A** | hsa-miR-16-1-3p | −25.9 | 0.632 | **398+** | hsa-miR-18a-3p | −30.3 | 1.680 |
| **168+A** | hsa-miR-185-3p | −27.7 | 0.632 | **398+** | hsa-miR-23a-5p | −31.3 | 1.680 |
| **168+A** | hsa-miR-298 | −27.9 | 0.632 | **398+** | hsa-miR-25-5p | −29.0 | 1.680 |
| **17+A** | hsa-miR-103a-3p | −26.9 | 0.561 | **398+** | hsa-miR-30a-3p | −28.4 | 1.680 |
| **17+A** | hsa-miR-152 | −26.8 | 0.561 | **398+** | hsa-miR-103a-2-5p | −27.5 | 1.680 |
| **17+A** | hsa-miR-409-5p | −27.6 | 0.561 | **398+** | hsa-miR-214-5p | −28.0 | 1.680 |
| **170+** | hsa-let-7a-2-3p | −28.9 | 0.545 | **398+** | hsa-miR-133a | −28.7 | 1.680 |
| **170+** | hsa-let-7e-3p | −32.4 | 0.545 | **398+** | hsa-miR-138-5p | −29.6 | 1.680 |
| **170+** | hsa-miR-25-5p | −30.7 | 0.545 | **398+** | hsa-miR-191-3p | −28.2 | 1.680 |
| **170+** | hsa-miR-211-3p | −28.5 | 0.545 | **398+** | hsa-miR-149-5p | −30.8 | 1.680 |
| **170+** | hsa-miR-365a-5p | −28.6 | 0.545 | **398+** | hsa-miR-149-3p | −29.5 | 1.680 |
| **170+** | hsa-miR-485-3p | −30.7 | 0.545 | **398+** | hsa-miR-193a-5p | −31.4 | 1.680 |
| **170+** | hsa-miR-298 | −31.9 | 0.545 | **398+** | hsa-miR-130b-5p | −27.2 | 1.680 |
| **170+** | hsa-miR-939-5p | −33.8 | 0.545 | **398+** | hsa-miR-328 | −33.8 | 1.680 |
| **170+A** | hsa-miR-27a-5p | −27.6 | 0.587 | **398+** | hsa-miR-342-5p | −27.3 | 1.680 |
| **170+A** | hsa-miR-197-5p | −30.3 | 0.587 | **398+** | hsa-miR-324-3p | −28.5 | 1.680 |
| **170+A** | hsa-miR-211-3p | −31.5 | 0.587 | **398+** | hsa-miR-133b | −28.7 | 1.680 |
| **170+A** | hsa-miR-9-5p | −27.0 | 0.587 | **398+** | hsa-miR-346 | −41.2 | 1.680 |
| **170+A** | hsa-miR-920 | −27.6 | 0.587 | **398+** | hsa-miR-423-3p | −30.1 | 1.680 |
| **170+A** | hsa-miR-939-5p | −33.0 | 0.587 | **398+** | hsa-miR-491-5p | −28.6 | 1.680 |
| **172+A** | hsa-miR-92a-2-5p | −27.7 | 1.317 | **398+** | hsa-miR-146b-3p | −29.1 | 1.680 |
| **177+A** | hsa-miR-103a-2-5p | −27.1 | 0.508 | **398+** | hsa-miR-512-3p | −27.9 | 1.680 |
| **177+A** | hsa-miR-214-5p | −29.0 | 0.508 | **398+** | hsa-miR-572 | −36.1 | 1.680 |
| **177+A** | hsa-let-7i-3p | −28.0 | 0.508 | **398+** | hsa-miR-584-3p | −29.5 | 1.680 |
| **177+A** | hsa-miR-188-5p | −32.0 | 0.508 | **398+** | hsa-miR-585 | −26.9 | 1.680 |
| **177+A** | hsa-miR-370 | −28.4 | 0.508 | **398+** | hsa-miR-593-5p | −29.8 | 1.680 |
| **177+A** | hsa-miR-623 | −29.1 | 0.508 | **398+** | hsa-miR-608 | −32.2 | 1.680 |
| **179+** | hsa-miR-212-5p | −27.4 | 1.121 | **398+** | hsa-miR-612 | −31.2 | 1.680 |
| **179+** | hsa-miR-504 | −27.4 | 1.121 | **398+** | hsa-miR-615-5p | −34.0 | 1.680 |
| **179+** | hsa-miR-514a-5p | −30.8 | 1.121 | **398+** | hsa-miR-621 | −28.6 | 1.680 |
| **179+** | hsa-miR-608 | −29.4 | 1.121 | **398+** | hsa-miR-625-5p | −27.5 | 1.680 |
| **179+** | hsa-miR-638 | −31.0 | 1.121 | **398+** | hsa-miR-636 | −41.0 | 1.680 |
| **179+** | hsa-miR-885-3p | −28.3 | 1.121 | **398+** | hsa-miR-663a | −34.8 | 1.680 |
| **18+** | hsa-miR-214-5p | −28.4 | 1.726 | **398+** | hsa-miR-658 | −33.6 | 1.680 |
| **18+** | hsa-miR-328 | −28.2 | 1.726 | **398+** | hsa-miR-659-3p | −29.6 | 1.680 |
| **18+** | hsa-miR-323a-5p | −27.8 | 1.726 | **398+** | hsa-miR-671-3p | −30.2 | 1.680 |
| **18+** | hsa-miR-647 | −26.2 | 1.726 | **398+** | hsa-miR-766-3p | −36.3 | 1.680 |
| **18+** | hsa-miR-663a | −32.0 | 1.726 | **398+** | hsa-miR-762 | −32.2 | 1.680 |
| **18+** | hsa-miR-764 | −27.5 | 1.726 | **398+** | hsa-miR-744-5p | −39.1 | 1.680 |
| **180+** | hsa-miR-197-5p | −28.8 | 0.758 | **398+** | hsa-miR-760 | −29.0 | 1.680 |
| **180+** | hsa-miR-139-3p | −30.1 | 0.758 | **398+** | hsa-miR-920 | −28.7 | 1.680 |
| **180+** | hsa-miR-187-3p | −27.3 | 0.758 | **398+** | hsa-miR-937-3p | −34.1 | 1.680 |
| **180+** | hsa-miR-143-5p | −26.7 | 0.758 | **398+** | hsa-miR-939-5p | −37.6 | 1.680 |
| **180+** | hsa-miR-511 | −27.7 | 0.758 | **398+** | hsa-miR-663b | −35.2 | 1.680 |
| **180+** | hsa-miR-885-3p | −30.7 | 0.758 | **398+** | hsa-miR-718 | −37.3 | 1.680 |
| **180+A** | hsa-miR-92a-3p | −28.3 | 0.801 | **398+A** | hsa-miR-219-1-3p | −28.3 | 1.680 |
| **180+A** | hsa-miR-143-5p | −28.3 | 0.801 | **398+A** | hsa-miR-222-3p | −27.9 | 1.680 |
| **180+A** | hsa-miR-345-5p | −30.4 | 0.801 | **398+A** | hsa-miR-125b-1-3p | −29.4 | 1.680 |
| **180+A** | hsa-miR-346 | −32.3 | 0.801 | **398+A** | hsa-miR-143-5p | −27.9 | 1.680 |
| **180+A** | hsa-miR-493-3p | −27.9 | 0.801 | **398+A** | hsa-miR-149-3p | −34.1 | 1.680 |
| **180+A** | hsa-miR-508-5p | −28.2 | 0.801 | **398+A** | hsa-miR-185-3p | −31.3 | 1.680 |
| **180+A** | hsa-miR-632 | −25.6 | 0.801 | **398+A** | hsa-miR-365b-5p | −28.2 | 1.680 |
| **180+A** | hsa-miR-639 | −31.2 | 0.801 | **398+A** | hsa-miR-370 | −34.1 | 1.680 |
| **180+A** | hsa-miR-670 | −28.7 | 0.801 | **398+A** | hsa-miR-328 | −34.6 | 1.680 |
| **181+** | hsa-miR-26b-3p | −27.2 | 0.772 | **398+A** | hsa-miR-323a-5p | −29.5 | 1.680 |
| **181+** | hsa-miR-197-5p | −30.3 | 0.772 | **398+A** | hsa-miR-324-5p | −29.7 | 1.680 |
| **181+** | hsa-miR-185-5p | −28.3 | 0.772 | **398+A** | hsa-miR-423-3p | −37.3 | 1.680 |
| **181+** | hsa-miR-185-3p | −36.9 | 0.772 | **398+A** | hsa-miR-18b-3p | −28.7 | 1.680 |
| **181+** | hsa-miR-365a-5p | −29.3 | 0.772 | **398+A** | hsa-miR-483-5p | −30.5 | 1.680 |
| **181+** | hsa-miR-767-3p | −29.3 | 0.772 | **398+A** | hsa-miR-491-5p | −28.9 | 1.680 |
| **182+** | hsa-miR-143-5p | −29.3 | 0.555 | **398+A** | hsa-miR-511 | −30.4 | 1.680 |
| **182+** | hsa-miR-150-3p | −27.9 | 0.555 | **398+A** | hsa-miR-512-3p | −28.7 | 1.680 |
| **182+** | hsa-miR-593-5p | −30.8 | 0.555 | **398+A** | hsa-miR-566 | −30.4 | 1.680 |
| **182+** | hsa-miR-650 | −27.3 | 0.555 | **398+A** | hsa-miR-572 | −29.6 | 1.680 |
| **182+** | hsa-miR-548q | −27.4 | 0.555 | **398+A** | hsa-miR-575 | −35.1 | 1.680 |
| **183+** | hsa-let-7d-5p | −28.0 | 0.703 | **398+A** | hsa-miR-550a-5p | −31.2 | 1.680 |
| **183+** | hsa-miR-19b-1-5p | −28.4 | 0.703 | **398+A** | hsa-miR-593-3p | −28.5 | 1.680 |
| **183+** | hsa-miR-19b-2-5p | −26.8 | 0.703 | **398+A** | hsa-miR-608 | −37.8 | 1.680 |
| **183+** | hsa-miR-187-5p | −27.8 | 0.703 | **398+A** | hsa-miR-609 | −26.8 | 1.680 |
| **183+** | hsa-miR-212-5p | −27.9 | 0.703 | **398+A** | hsa-miR-612 | −30.9 | 1.680 |
| **183+** | hsa-let-7i-3p | −27.3 | 0.703 | **398+A** | hsa-miR-615-3p | −31.9 | 1.680 |
| **183+** | hsa-miR-143-5p | −32.1 | 0.703 | **398+A** | hsa-miR-619 | −31.1 | 1.680 |
| **183+** | hsa-miR-149-3p | −29.0 | 0.703 | **398+A** | hsa-miR-623 | −32.9 | 1.680 |
| **183+** | hsa-miR-150-3p | −29.7 | 0.703 | **398+A** | hsa-miR-631 | −28.3 | 1.680 |
| **183+** | hsa-miR-432-5p | −28.7 | 0.703 | **398+A** | hsa-miR-636 | −33.1 | 1.680 |
| **183+** | hsa-miR-450a-3p | −25.7 | 0.703 | **398+A** | hsa-miR-638 | −31.8 | 1.680 |
| **183+** | hsa-miR-564 | −26.4 | 0.703 | **398+A** | hsa-miR-661 | −32.2 | 1.680 |
| **183+** | hsa-miR-585 | −26.2 | 0.703 | **398+A** | hsa-miR-663a | −38.3 | 1.680 |
| **183+** | hsa-miR-588 | −31.2 | 0.703 | **398+A** | hsa-miR-550a-3-5p | −26.9 | 1.680 |
| **183+** | hsa-miR-593-5p | −31.2 | 0.703 | **398+A** | hsa-miR-769-3p | −33.8 | 1.680 |
| **183+** | hsa-miR-612 | −32.1 | 0.703 | **398+A** | hsa-miR-762 | −43.5 | 1.680 |
| **183+** | hsa-miR-637 | −31.4 | 0.703 | **398+A** | hsa-miR-765 | −27.8 | 1.680 |
| **183+** | hsa-miR-642a-3p | −26.8 | 0.703 | **398+A** | hsa-miR-675-5p | −30.5 | 1.680 |
| **183+** | hsa-miR-650 | −28.8 | 0.703 | **398+A** | hsa-miR-874 | −31.1 | 1.680 |
| **183+** | hsa-miR-661 | −32.7 | 0.703 | **398+A** | hsa-miR-744-5p | −30.3 | 1.680 |
| **183+** | hsa-miR-657 | −28.5 | 0.703 | **398+A** | hsa-miR-877-5p | −29.7 | 1.680 |
| **183+** | hsa-miR-449c-5p | −28.0 | 0.703 | **398+A** | hsa-miR-760 | −29.3 | 1.680 |
| **183+** | hsa-miR-765 | −26.9 | 0.703 | **398+A** | hsa-miR-936 | −31.3 | 1.680 |
| **183+** | hsa-miR-675-5p | −30.5 | 0.703 | **398+A** | hsa-miR-937-5p | −28.4 | 1.680 |
| **183+** | hsa-miR-147b | −28.6 | 0.703 | **398+A** | hsa-miR-939-5p | −32.1 | 1.680 |
| **183+** | hsa-miR-939-5p | −30.5 | 0.703 | **398+A** | hsa-miR-663b | −30.2 | 1.680 |
| **183+** | hsa-miR-548v | −25.8 | 0.703 | **398+A** | hsa-miR-718 | −29.4 | 1.680 |
| **183+** | hsa-miR-642b-3p | −30.2 | 0.703 | **399+** | hsa-miR-15a-3p | −27.5 | 1.192 |
| **185+** | hsa-miR-197-5p | −31.5 | 0.679 | **399+** | hsa-miR-185-3p | −29.2 | 1.192 |
| **185+** | hsa-miR-516b-5p | −29.8 | 0.679 | **399+** | hsa-miR-569 | −25.6 | 1.192 |
| **185+** | hsa-miR-572 | −30.0 | 0.679 | **399+A** | hsa-miR-638 | −29.8 | 1.340 |
| **185+** | hsa-miR-593-5p | −31.5 | 0.679 | **4+** | hsa-miR-615-5p | −34.6 | 0.452 |
| **185+** | hsa-miR-608 | −31.7 | 0.679 | **402+** | hsa-miR-143-5p | −32.1 | 0.717 |
| **185+** | hsa-miR-767-5p | −29.9 | 0.679 | **402+** | hsa-miR-106b-3p | −28.7 | 0.717 |
| **185+** | hsa-miR-892c-5p | −28.2 | 0.679 | **402+** | hsa-miR-423-3p | −28.3 | 0.717 |
| **185+A** | hsa-miR-92a-1-5p | −29.6 | 0.777 | **402+** | hsa-miR-671-5p | −29.4 | 0.717 |
| **185+A** | hsa-miR-296-3p | −30.9 | 0.777 | **402+A** | hsa-miR-325 | −27.3 | 0.609 |
| **185+A** | hsa-miR-146b-3p | −31.5 | 0.777 | **402+A** | hsa-miR-554 | −28.6 | 0.609 |
| **185+A** | hsa-miR-601 | −28.7 | 0.777 | **402+A** | hsa-miR-621 | −28.1 | 0.609 |
| **185+A** | hsa-miR-637 | −30.4 | 0.777 | **402+A** | hsa-miR-449c-3p | −29.6 | 0.609 |
| **185+A** | hsa-miR-661 | −30.9 | 0.777 | **403+** | hsa-miR-29b-1-5p | −27.8 | 0.898 |
| **185+A** | hsa-miR-663b | −32.0 | 0.777 | **403+** | hsa-miR-197-5p | −30.5 | 0.898 |
| **188+** | hsa-miR-548aw | −24.8 | 0.595 | **403+** | hsa-miR-939-5p | −30.9 | 0.898 |
| **189+** | hsa-miR-92a-1-5p | −30.9 | 0.404 | **403+A** | hsa-miR-198 | −31.5 | 0.898 |
| **189+** | hsa-miR-197-3p | −30.4 | 0.404 | **403+A** | hsa-miR-412 | −27.1 | 0.898 |
| **189+** | hsa-miR-138-5p | −33.9 | 0.404 | **403+A** | hsa-miR-658 | −31.9 | 0.898 |
| **189+** | hsa-miR-484 | −32.3 | 0.404 | **404+** | hsa-miR-198 | −31.5 | 0.447 |
| **189+** | hsa-miR-645 | −31.0 | 0.404 | **404+** | hsa-miR-525-5p | −29.3 | 0.447 |
| **189+** | hsa-miR-548au-3p | −28.3 | 0.405 | **404+** | hsa-miR-617 | −26.8 | 0.447 |
| **19+** | hsa-miR-193b-5p | −27.8 | 0.601 | **404+** | hsa-miR-933 | −28.8 | 0.447 |
| **19+** | hsa-miR-512-3p | −28.5 | 0.601 | **408+A** | hsa-miR-18a-3p | −28.2 | 0.599 |
| **19+** | hsa-miR-623 | −28.9 | 0.601 | **408+A** | hsa-miR-107 | −27.6 | 0.599 |
| **19+** | hsa-miR-638 | −30.6 | 0.601 | **408+A** | hsa-miR-23b-5p | −27.3 | 0.599 |
| **190+A** | hsa-miR-204-3p | −30.5 | 0.507 | **408+A** | hsa-miR-193a-3p | −27.1 | 0.599 |
| **190+A** | hsa-miR-124-5p | −29.6 | 0.507 | **408+A** | hsa-miR-296-5p | −30.5 | 0.599 |
| **190+A** | hsa-miR-149-3p | −30.2 | 0.507 | **408+A** | hsa-miR-324-3p | −29.2 | 0.599 |
| **190+A** | hsa-miR-320a | −29.0 | 0.507 | **408+A** | hsa-miR-345-3p | −28.8 | 0.599 |
| **190+A** | hsa-miR-193b-5p | −33.5 | 0.507 | **408+A** | hsa-miR-423-3p | −32.2 | 0.599 |
| **190+A** | hsa-miR-508-3p | −28.0 | 0.507 | **408+A** | hsa-miR-18b-3p | −31.3 | 0.599 |
| **190+A** | hsa-miR-320b | −29.3 | 0.507 | **408+A** | hsa-miR-490-5p | −27.7 | 0.599 |
| **190+A** | hsa-miR-320c | −25.5 | 0.507 | **408+A** | hsa-miR-146b-3p | −28.5 | 0.599 |
| **190+A** | hsa-miR-762 | −34.5 | 0.507 | **408+A** | hsa-miR-504 | −28.8 | 0.599 |
| **190+A** | hsa-miR-548v | −25.4 | 0.507 | **408+A** | hsa-miR-564 | −26.4 | 0.599 |
| **191+A** | hsa-miR-34c-5p | −26.6 | 0.877 | **408+A** | hsa-miR-584-3p | −28.2 | 0.599 |
| **191+A** | hsa-miR-345-5p | −27.8 | 0.877 | **408+A** | hsa-miR-550a-5p | −32.5 | 0.599 |
| **192+A** | hsa-let-7g-3p | −29.2 | 1.342 | **408+A** | hsa-miR-602 | −34.8 | 0.599 |
| **192+A** | hsa-miR-320a | −29.8 | 1.342 | **408+A** | hsa-miR-611 | −36.6 | 0.599 |
| **192+A** | hsa-miR-323a-5p | −28.2 | 1.342 | **408+A** | hsa-miR-615-5p | −32.7 | 0.599 |
| **192+A** | hsa-miR-320b | −29.8 | 1.342 | **408+A** | hsa-miR-33b-3p | −34.3 | 0.599 |
| **192+A** | hsa-miR-320c | −27.3 | 1.342 | **408+A** | hsa-miR-650 | −27.2 | 0.599 |
| **192+A** | hsa-miR-762 | −29.3 | 1.342 | **408+A** | hsa-miR-661 | −31.3 | 0.599 |
| **195+** | hsa-miR-26a-1-3p | −27.8 | 0.389 | **408+A** | hsa-miR-659-5p | −31.8 | 0.599 |
| **195+** | hsa-miR-31-5p | −27.4 | 0.389 | **408+A** | hsa-miR-762 | −31.3 | 0.599 |
| **195+** | hsa-miR-107 | −27.2 | 0.389 | **408+A** | hsa-miR-874 | −28.5 | 0.599 |
| **195+** | hsa-miR-151a-3p | −27.2 | 0.389 | **408+A** | hsa-miR-877-3p | −31.0 | 0.599 |
| **195+** | hsa-miR-339-5p | −29.6 | 0.389 | **408+A** | hsa-miR-937-5p | −27.9 | 0.599 |
| **195+** | hsa-miR-431-3p | −30.4 | 0.389 | **408+A** | hsa-miR-939-5p | −29.3 | 0.599 |
| **195+** | hsa-miR-488-5p | −27.0 | 0.389 | **408+A** | hsa-miR-939-3p | −29.0 | 0.599 |
| **195+** | hsa-miR-502-5p | −27.9 | 0.389 | **410+** | hsa-miR-664b-5p | −28.4 | 0.868 |
| **195+** | hsa-miR-596 | −31.2 | 0.389 | **412+A** | hsa-miR-214-5p | −27.9 | 0.439 |
| **195+** | hsa-miR-602 | −32.5 | 0.389 | **412+A** | hsa-let-7i-3p | −30.7 | 0.439 |
| **195+** | hsa-miR-612 | −30.4 | 0.389 | **412+A** | hsa-miR-361-3p | −27.5 | 0.439 |
| **195+** | hsa-miR-623 | −32.1 | 0.389 | **412+A** | hsa-miR-346 | −29.8 | 0.439 |
| **195+** | hsa-miR-637 | −33.4 | 0.389 | **412+A** | hsa-miR-596 | −32.8 | 0.439 |
| **195+** | hsa-miR-661 | −33.1 | 0.389 | **412+A** | hsa-miR-650 | −27.4 | 0.439 |
| **195+** | hsa-miR-657 | −30.4 | 0.389 | **414+** | hsa-miR-197-5p | −32.9 | 1.274 |
| **195+** | hsa-miR-663b | −30.2 | 0.389 | **414+** | hsa-miR-34a-5p | −28.9 | 1.274 |
| **195+** | hsa-miR-718 | −30.6 | 0.389 | **414+** | hsa-miR-210 | −29.9 | 1.274 |
| **195+** | hsa-miR-323b-5p | −28.1 | 0.389 | **414+** | hsa-miR-125b-1-3p | −29.1 | 1.274 |
| **196+** | hsa-miR-28-5p | −26.8 | 0.737 | **414+** | hsa-miR-149-3p | −30.5 | 1.274 |
| **196+** | hsa-miR-31-5p | −26.5 | 0.737 | **414+** | hsa-miR-296-3p | −33.6 | 1.274 |
| **196+** | hsa-miR-129-5p | −26.6 | 0.737 | **414+** | hsa-miR-363-5p | −27.6 | 1.274 |
| **196+** | hsa-miR-151a-5p | −29.7 | 0.737 | **414+** | hsa-miR-373-3p | −31.6 | 1.274 |
| **196+** | hsa-miR-431-5p | −28.3 | 0.737 | **414+** | hsa-miR-323a-5p | −29.6 | 1.274 |
| **196+** | hsa-miR-151b | −29.7 | 0.737 | **414+** | hsa-miR-485-5p | −28.9 | 1.274 |
| **196+** | hsa-miR-708-5p | −27.3 | 0.737 | **414+** | hsa-miR-486-3p | −28.4 | 1.274 |
| **196+** | hsa-miR-939-5p | −31.9 | 0.737 | **414+** | hsa-miR-503-5p | −29.2 | 1.274 |
| **20+** | hsa-miR-27a-5p | −29.1 | 0.262 | **414+** | hsa-miR-572 | −31.0 | 1.274 |
| **20+** | hsa-miR-23b-5p | −27.3 | 0.262 | **414+** | hsa-miR-608 | −29.6 | 1.274 |
| **20+** | hsa-miR-296-5p | −31.0 | 0.262 | **414+** | hsa-miR-612 | −34.0 | 1.274 |
| **20+** | hsa-miR-339-5p | −30.0 | 0.262 | **414+** | hsa-miR-615-5p | −29.6 | 1.274 |
| **20+** | hsa-miR-484 | −32.3 | 0.262 | **414+** | hsa-miR-663a | −34.1 | 1.274 |
| **20+** | hsa-miR-502-5p | −31.9 | 0.262 | **414+** | hsa-miR-658 | −31.5 | 1.274 |
| **20+** | hsa-miR-584-3p | −28.4 | 0.262 | **414+** | hsa-miR-671-3p | −30.3 | 1.274 |
| **20+** | hsa-miR-610 | −28.5 | 0.262 | **414+** | hsa-miR-668 | −30.2 | 1.274 |
| **20+** | hsa-miR-642a-5p | −29.9 | 0.262 | **414+** | hsa-miR-298 | −30.1 | 1.274 |
| **20+** | hsa-miR-658 | −35.3 | 0.262 | **414+** | hsa-miR-890 | −26.8 | 1.274 |
| **20+** | hsa-miR-762 | −29.4 | 0.262 | **414+** | hsa-miR-541-3p | −27.3 | 1.274 |
| **20+** | hsa-miR-675-3p | −29.9 | 0.262 | **414+** | hsa-miR-885-3p | −29.2 | 1.274 |
| **20+** | hsa-miR-708-5p | −27.3 | 0.262 | **414+** | hsa-miR-877-5p | −29.4 | 1.274 |
| **20+** | hsa-miR-877-3p | −33.8 | 0.262 | **414+** | hsa-miR-939-5p | −29.5 | 1.274 |
| **20+** | hsa-miR-940 | −39.2 | 0.262 | **416+** | hsa-miR-34a-5p | −28.0 | 1.163 |
| **20+** | hsa-miR-718 | −29.1 | 0.262 | **416+** | hsa-miR-210 | −29.5 | 1.163 |
| **200+** | hsa-miR-197-5p | −29.6 | 0.498 | **416+** | hsa-miR-145-3p | −26.5 | 1.163 |
| **200+** | hsa-miR-214-3p | −27.8 | 0.498 | **416+** | hsa-miR-323a-5p | −30.6 | 1.163 |
| **200+** | hsa-miR-34c-5p | −29.1 | 0.498 | **416+** | hsa-miR-339-5p | −31.5 | 1.163 |
| **200+** | hsa-miR-346 | −37.2 | 0.498 | **416+** | hsa-miR-449a | −26.8 | 1.163 |
| **200+** | hsa-miR-431-5p | −27.0 | 0.498 | **416+** | hsa-miR-486-5p | −29.8 | 1.163 |
| **200+** | hsa-miR-92b-5p | −30.5 | 0.498 | **416+** | hsa-miR-508-3p | −26.9 | 1.163 |
| **200+** | hsa-miR-608 | −31.3 | 0.498 | **416+** | hsa-miR-615-5p | −33.0 | 1.163 |
| **200+** | hsa-miR-623 | −28.2 | 0.498 | **416+** | hsa-miR-638 | −31.2 | 1.163 |
| **200+** | hsa-miR-645 | −25.8 | 0.498 | **416+** | hsa-miR-663a | −30.0 | 1.163 |
| **200+** | hsa-miR-650 | −29.4 | 0.498 | **416+** | hsa-miR-762 | −31.3 | 1.163 |
| **200+** | hsa-miR-449c-5p | −29.1 | 0.498 | **416+** | hsa-miR-885-3p | −31.3 | 1.163 |
| **200+** | hsa-miR-449c-3p | −30.9 | 0.498 | **416+** | hsa-miR-939-5p | −29.7 | 1.163 |
| **200+** | hsa-miR-663b | −29.0 | 0.498 | **416+** | hsa-miR-378g | −26.8 | 1.163 |
| **200+** | hsa-miR-664b-3p | −34.5 | 0.498 | **417+** | hsa-miR-187-5p | −29.3 | 0.784 |
| **201+** | hsa-let-7a-2-3p | −29.5 | 1.200 | **417+** | hsa-miR-221-3p | −27.0 | 0.784 |
| **201+** | hsa-miR-129-5p | −27.4 | 1.200 | **417+** | hsa-miR-222-3p | −28.6 | 0.784 |
| **201+** | hsa-let-7i-3p | −26.7 | 1.200 | **417+** | hsa-miR-423-3p | −29.9 | 0.784 |
| **201+** | hsa-miR-423-3p | −31.6 | 1.200 | **417+** | hsa-miR-433 | −26.8 | 0.784 |
| **201+** | hsa-miR-512-5p | −30.8 | 1.200 | **417+** | hsa-miR-554 | −26.6 | 0.784 |
| **201+** | hsa-miR-572 | −30.9 | 1.200 | **417+** | hsa-miR-584-5p | −26.3 | 0.784 |
| **201+** | hsa-miR-593-5p | −33.7 | 1.200 | **417+** | hsa-miR-647 | −27.8 | 0.784 |
| **201+** | hsa-miR-596 | −28.6 | 1.200 | **417+** | hsa-miR-661 | −34.7 | 0.784 |
| **201+** | hsa-miR-671-3p | −28.3 | 1.200 | **417+** | hsa-miR-323b-5p | −29.1 | 0.784 |
| **201+** | hsa-miR-663b | −29.6 | 1.200 | **419+** | hsa-miR-196a-5p | −29.6 | 1.187 |
| **208+** | hsa-miR-934 | −27.9 | 0.812 | **419+** | hsa-miR-196b-5p | −29.6 | 1.187 |
| **208+A** | hsa-miR-423-5p | −28.4 | 0.812 | **419+** | hsa-miR-501-5p | −28.7 | 1.187 |
| **21+A** | hsa-miR-585 | −25.5 | 0.485 | **419+** | hsa-miR-638 | −35.1 | 1.187 |
| **21+A** | hsa-miR-371b-5p | −27.3 | 0.485 | **419+** | hsa-miR-939-5p | −31.5 | 1.187 |
| **21+A** | hsa-miR-548au-3p | −25.8 | 0.485 | **420+** | hsa-miR-323a-3p | −27.6 | 0.670 |
| **213+A** | hsa-let-7i-3p | −27.8 | 0.287 | **420+** | hsa-miR-449c-5p | −28.2 | 0.670 |
| **213+A** | hsa-miR-342-5p | −27.4 | 0.287 | **420+** | hsa-miR-513c-5p | −26.0 | 0.670 |
| **213+A** | hsa-miR-608 | −29.0 | 0.287 | **420+** | hsa-miR-514b-5p | −26.9 | 0.670 |
| **213+A** | hsa-miR-663b | −29.6 | 0.287 | **421+** | hsa-miR-27a-5p | −29.9 | 1.165 |
| **215+A** | hsa-miR-204-3p | −27.7 | 0.694 | **421+** | hsa-miR-34a-3p | −29.4 | 1.165 |
| **215+A** | hsa-miR-149-3p | −31.4 | 0.694 | **421+** | hsa-miR-181c-3p | −28.0 | 1.165 |
| **215+A** | hsa-miR-320a | −35.2 | 0.694 | **421+** | hsa-miR-212-3p | −28.1 | 1.165 |
| **215+A** | hsa-miR-296-3p | −30.5 | 0.694 | **421+** | hsa-miR-125b-1-3p | −29.8 | 1.165 |
| **215+A** | hsa-miR-637 | −29.5 | 0.694 | **421+** | hsa-miR-132-3p | −28.2 | 1.165 |
| **215+A** | hsa-miR-320b | −35.2 | 0.694 | **421+** | hsa-miR-145-5p | −29.5 | 1.165 |
| **215+A** | hsa-miR-320c | −32.7 | 0.694 | **421+** | hsa-miR-381-5p | −30.5 | 1.165 |
| **215+A** | hsa-miR-766-5p | −27.2 | 0.694 | **421+** | hsa-miR-331-3p | −28.8 | 1.165 |
| **215+A** | hsa-miR-708-5p | −29.2 | 0.694 | **421+** | hsa-miR-346 | −30.9 | 1.165 |
| **215+A** | hsa-miR-320d | −28.0 | 0.694 | **421+** | hsa-miR-412 | −30.3 | 1.165 |
| **217+A** | hsa-miR-187-5p | −27.8 | 0.215 | **421+** | hsa-miR-578 | −29.9 | 1.165 |
| **217+A** | hsa-miR-218-2-3p | −31.7 | 0.215 | **421+** | hsa-miR-615-5p | −31.5 | 1.165 |
| **217+A** | hsa-miR-125a-3p | −27.3 | 0.215 | **421+** | hsa-miR-632 | −28.4 | 1.165 |
| **217+A** | hsa-miR-330-3p | −28.2 | 0.215 | **421+** | hsa-miR-663a | −31.8 | 1.165 |
| **217+A** | hsa-miR-339-3p | −28.8 | 0.215 | **421+** | hsa-miR-657 | −30.1 | 1.165 |
| **217+A** | hsa-miR-506-3p | −30.2 | 0.215 | **421+** | hsa-miR-762 | −33.6 | 1.165 |
| **217+A** | hsa-miR-572 | −29.1 | 0.215 | **421+** | hsa-miR-920 | −28.4 | 1.165 |
| **217+A** | hsa-miR-632 | −25.3 | 0.215 | **421+** | hsa-miR-323b-5p | −32.9 | 1.165 |
| **217+A** | hsa-miR-663a | −29.8 | 0.215 | **421+** | hsa-miR-664b-3p | −30.6 | 1.165 |
| **217+A** | hsa-miR-500b | −27.5 | 0.215 | **422+** | hsa-miR-92a-3p | −27.6 | 1.380 |
| **218+** | hsa-miR-520e | −26.0 | 0.713 | **422+** | hsa-miR-29b-2-5p | −26.5 | 1.380 |
| **218+** | hsa-miR-519e-3p | −27.5 | 0.713 | **422+** | hsa-miR-217 | −27.3 | 1.380 |
| **218+** | hsa-miR-520f | −28.2 | 0.713 | **422+** | hsa-miR-218-1-3p | −27.2 | 1.380 |
| **218+** | hsa-miR-519c-3p | −26.7 | 0.713 | **422+** | hsa-miR-221-3p | −32.5 | 1.380 |
| **218+** | hsa-miR-519b-3p | −27.8 | 0.713 | **422+** | hsa-miR-222-3p | −26.8 | 1.380 |
| **218+** | hsa-miR-520b | −28.9 | 0.713 | **422+** | hsa-miR-140-5p | −27.6 | 1.380 |
| **218+** | hsa-miR-520c-3p | −28.9 | 0.713 | **422+** | hsa-miR-370 | −33.2 | 1.380 |
| **218+** | hsa-miR-520d-3p | −29.6 | 0.713 | **422+** | hsa-miR-515-3p | −26.4 | 1.380 |
| **218+** | hsa-miR-555 | −26.8 | 0.713 | **422+** | hsa-miR-551a | −29.0 | 1.380 |
| **220+** | hsa-miR-181a-2-3p | −27.7 | 0.567 | **422+** | hsa-miR-574-5p | −26.8 | 1.380 |
| **220+** | hsa-miR-497-3p | −26.4 | 0.567 | **422+** | hsa-miR-608 | −30.9 | 1.380 |
| **220+** | hsa-miR-516b-3p | −32.7 | 0.567 | **422+** | hsa-miR-615-5p | −30.7 | 1.380 |
| **220+** | hsa-miR-516a-3p | −32.7 | 0.567 | **422+** | hsa-miR-623 | −30.6 | 1.380 |
| **223+A** | hsa-miR-185-3p | −28.3 | 0.740 | **422+** | hsa-miR-632 | −28.6 | 1.380 |
| **223+A** | hsa-miR-642a-5p | −29.9 | 0.740 | **422+** | hsa-miR-636 | −32.5 | 1.380 |
| **223+A** | hsa-miR-650 | −29.3 | 0.740 | **422+** | hsa-miR-661 | −32.3 | 1.380 |
| **223+A** | hsa-miR-642b-5p | −29.7 | 0.740 | **422+** | hsa-miR-671-5p | −32.4 | 1.380 |
| **227+A** | hsa-miR-219-5p | −26.4 | 0.854 | **422+** | hsa-miR-760 | −28.8 | 1.380 |
| **229+A** | hsa-miR-488-5p | −27.5 | 0.252 | **422+** | hsa-miR-642b-5p | −28.3 | 1.380 |
| **229+A** | hsa-miR-518f-5p | −27.9 | 0.252 | **425+A** | hsa-miR-23a-5p | −30.2 | 0.734 |
| **234+** | hsa-miR-18b-3p | −27.8 | 0.401 | **425+A** | hsa-miR-27a-5p | −30.0 | 0.734 |
| **238+** | hsa-miR-103a-3p | −28.8 | 0.331 | **425+A** | hsa-miR-223-5p | −29.2 | 0.734 |
| **238+** | hsa-miR-185-3p | −32.9 | 0.331 | **425+A** | hsa-miR-194-3p | −30.3 | 0.734 |
| **238+** | hsa-miR-301a-5p | −28.1 | 0.331 | **425+A** | hsa-miR-365a-5p | −32.5 | 0.734 |
| **238+** | hsa-miR-365b-5p | −31.0 | 0.331 | **425+A** | hsa-miR-365b-5p | −28.9 | 0.734 |
| **238+** | hsa-miR-574-5p | −28.7 | 0.331 | **425+A** | hsa-miR-370 | −29.3 | 0.734 |
| **238+** | hsa-miR-613 | −27.4 | 0.331 | **425+A** | hsa-miR-517-5p | −30.6 | 0.734 |
| **238+** | hsa-miR-638 | −33.3 | 0.331 | **425+A** | hsa-miR-514a-3p | −29.8 | 0.734 |
| **238+** | hsa-miR-639 | −29.6 | 0.331 | **425+A** | hsa-miR-376a-2-5p | −26.5 | 0.734 |
| **238+** | hsa-miR-762 | −31.8 | 0.331 | **425+A** | hsa-miR-555 | −32.7 | 0.734 |
| **238+** | hsa-miR-744-3p | −29.8 | 0.331 | **425+A** | hsa-miR-615-5p | −29.7 | 0.734 |
| **238+** | hsa-miR-942 | −31.1 | 0.331 | **425+A** | hsa-miR-638 | −31.3 | 0.734 |
| **238+A** | hsa-let-7d-5p | −30.2 | 0.331 | **425+A** | hsa-miR-939-5p | −32.7 | 0.734 |
| **238+A** | hsa-let-7e-5p | −32.3 | 0.331 | **427+** | hsa-let-7b-5p | −26.7 | 0.466 |
| **238+A** | hsa-miR-197-5p | −29.8 | 0.331 | **427+** | hsa-miR-211-5p | −26.9 | 0.466 |
| **238+A** | hsa-miR-9-5p | −31.4 | 0.331 | **427+** | hsa-miR-219-1-3p | −30.0 | 0.466 |
| **238+A** | hsa-miR-149-3p | −31.3 | 0.331 | **427+** | hsa-miR-191-3p | −33.4 | 0.466 |
| **238+A** | hsa-miR-188-5p | −29.5 | 0.331 | **427+** | hsa-miR-326 | −27.6 | 0.466 |
| **238+A** | hsa-miR-512-5p | −29.9 | 0.331 | **427+** | hsa-miR-571 | −26.0 | 0.466 |
| **238+A** | hsa-miR-600 | −28.0 | 0.331 | **427+** | hsa-miR-574-5p | −28.2 | 0.466 |
| **238+A** | hsa-miR-608 | −33.3 | 0.331 | **427+** | hsa-miR-550a-5p | −28.3 | 0.466 |
| **238+A** | hsa-miR-625-5p | −27.4 | 0.331 | **427+** | hsa-miR-629-3p | −27.8 | 0.466 |
| **238+A** | hsa-miR-940 | −35.0 | 0.331 | **427+** | hsa-miR-636 | −32.9 | 0.466 |
| **238+A** | hsa-miR-942 | −29.5 | 0.331 | **427+** | hsa-miR-661 | −32.6 | 0.466 |
| **238+A** | hsa-miR-323b-5p | −30.2 | 0.331 | **427+** | hsa-miR-147b | −27.6 | 0.466 |
| **239+A** | hsa-miR-383 | −27.3 | 0.906 | **427+** | hsa-miR-877-3p | −38.6 | 0.466 |
| **239+A** | hsa-miR-769-3p | −29.9 | 0.906 | **427+** | hsa-miR-548q | −26.4 | 0.466 |
| **239+A** | hsa-miR-920 | −27.1 | 0.906 | **43+** | hsa-miR-103a-2-5p | −26.9 | 0.457 |
| **24+** | hsa-miR-15a-3p | −28.3 | 0.883 | **43+** | hsa-miR-646 | −28.4 | 0.457 |
| **24+** | hsa-miR-22-3p | −28.6 | 0.883 | **43+** | hsa-miR-657 | −31.2 | 0.457 |
| **24+** | hsa-miR-27a-5p | −29.1 | 0.883 | **43+** | hsa-miR-764 | −27.5 | 0.457 |
| **24+** | hsa-miR-181a-5p | −28.4 | 0.883 | **43+** | hsa-miR-744-5p | −27.7 | 0.457 |
| **24+** | hsa-miR-185-3p | −29.1 | 0.883 | **432+** | hsa-miR-660-3p | −29.5 | 0.694 |
| **24+** | hsa-miR-320a | −34.4 | 0.883 | **435+** | hsa-miR-143-5p | −27.1 | 0.533 |
| **24+** | hsa-miR-330-5p | −34.9 | 0.883 | **435+** | hsa-miR-29c-5p | −29.6 | 0.533 |
| **24+** | hsa-miR-570-5p | −27.2 | 0.883 | **435+** | hsa-miR-361-5p | −29.4 | 0.533 |
| **24+** | hsa-miR-638 | −34.3 | 0.883 | **436+** | hsa-let-7b-5p | −27.1 | 0.813 |
| **24+** | hsa-miR-639 | −31.2 | 0.883 | **436+** | hsa-let-7c | −26.4 | 0.813 |
| **24+** | hsa-miR-644a | −26.7 | 0.883 | **436+** | hsa-miR-196a-5p | −28.5 | 0.813 |
| **24+** | hsa-miR-650 | −28.1 | 0.883 | **436+** | hsa-let-7i-5p | −26.8 | 0.813 |
| **24+** | hsa-miR-320b | −35.1 | 0.883 | **436+** | hsa-miR-191-3p | −27.3 | 0.813 |
| **24+** | hsa-miR-320c | −31.2 | 0.883 | **436+** | hsa-miR-375 | −27.0 | 0.813 |
| **24+** | hsa-miR-762 | −30.5 | 0.883 | **436+** | hsa-miR-196b-5p | −28.5 | 0.813 |
| **24+** | hsa-miR-939-5p | −31.2 | 0.883 | **436+** | hsa-miR-432-5p | −27.0 | 0.813 |
| **24+** | hsa-miR-320d | −26.6 | 0.883 | **436+** | hsa-miR-512-5p | −27.5 | 0.813 |
| **24+** | hsa-miR-548ai | −27.2 | 0.883 | **436+** | hsa-miR-668 | −30.3 | 0.813 |
| **24+A** | hsa-let-7g-3p | −27.6 | 1.335 | **436+** | hsa-miR-670 | −27.9 | 0.813 |
| **24+A** | hsa-let-7i-3p | −28.6 | 1.335 | **436+** | hsa-miR-760 | −28.7 | 0.813 |
| **24+A** | hsa-miR-143-5p | −32.6 | 1.335 | **44+** | hsa-miR-27a-3p | −26.1 | 1.272 |
| **24+A** | hsa-miR-125a-3p | −30.6 | 1.335 | **44+** | hsa-miR-212-3p | −26.8 | 1.272 |
| **24+A** | hsa-miR-185-3p | −29.8 | 1.335 | **44+** | hsa-miR-221-3p | −26.9 | 1.272 |
| **24+A** | hsa-miR-377-5p | −28.5 | 1.335 | **44+** | hsa-miR-185-5p | −30.7 | 1.272 |
| **24+A** | hsa-miR-449c-5p | −29.9 | 1.335 | **44+** | hsa-miR-34c-5p | −29.3 | 1.272 |
| **24+A** | hsa-miR-760 | −28.5 | 1.335 | **44+** | hsa-miR-377-5p | −28.7 | 1.272 |
| **24+A** | hsa-miR-323b-5p | −28.9 | 1.335 | **44+** | hsa-miR-331-3p | −30.9 | 1.272 |
| **241+A** | hsa-miR-106b-3p | −26.8 | 1.331 | **44+** | hsa-miR-520a-5p | −26.7 | 1.272 |
| **241+A** | hsa-miR-500a-3p | −27.7 | 1.331 | **44+** | hsa-miR-525-5p | −29.4 | 1.272 |
| **241+A** | hsa-miR-501-3p | −29.6 | 1.331 | **44+** | hsa-miR-503-5p | −34.2 | 1.272 |
| **241+A** | hsa-miR-502-3p | −26.7 | 1.331 | **44+** | hsa-miR-554 | −28.2 | 1.272 |
| **241+A** | hsa-miR-645 | −27.0 | 1.331 | **44+** | hsa-miR-92b-5p | −28.3 | 1.272 |
| **244+** | hsa-miR-150-3p | −29.6 | 1.646 | **44+** | hsa-miR-616-3p | −29.1 | 1.272 |
| **244+** | hsa-miR-765 | −27.9 | 1.646 | **44+** | hsa-miR-621 | −26.8 | 1.272 |
| **246+** | hsa-miR-31-5p | −26.8 | 0.540 | **44+** | hsa-miR-646 | −25.5 | 1.272 |
| **246+** | hsa-miR-330-5p | −31.1 | 0.540 | **44+** | hsa-miR-671-5p | −29.7 | 1.272 |
| **246+** | hsa-miR-337-3p | −31.9 | 0.540 | **44+** | hsa-miR-885-3p | −28.6 | 1.272 |
| **246+** | hsa-miR-326 | −28.7 | 0.540 | **44+** | hsa-miR-663b | −28.6 | 1.272 |
| **246+** | hsa-miR-346 | −32.6 | 0.540 | **44+** | hsa-miR-548s | −26.6 | 1.272 |
| **246+** | hsa-miR-484 | −29.4 | 0.540 | **44+** | hsa-miR-378e | −27.8 | 1.272 |
| **246+** | hsa-miR-432-3p | −28.3 | 0.540 | **44+** | hsa-miR-378h | −26.5 | 1.272 |
| **246+** | hsa-miR-193b-5p | −29.0 | 0.540 | **44+A** | hsa-miR-16-1-3p | −26.5 | 0.468 |
| **246+** | hsa-miR-509-3p | −27.6 | 0.540 | **44+A** | hsa-miR-96-5p | −27.4 | 0.468 |
| **246+** | hsa-miR-575 | −33.1 | 0.540 | **44+A** | hsa-miR-211-3p | −27.2 | 0.468 |
| **246+** | hsa-miR-584-3p | −30.9 | 0.540 | **44+A** | hsa-miR-145-5p | −28.4 | 0.468 |
| **246+** | hsa-miR-661 | −30.5 | 0.540 | **44+A** | hsa-miR-138-1-3p | −28.1 | 0.468 |
| **246+** | hsa-miR-541-5p | −28.9 | 0.540 | **44+A** | hsa-miR-185-5p | −27.2 | 0.468 |
| **246+** | hsa-miR-744-5p | −28.5 | 0.540 | **44+A** | hsa-miR-185-3p | −35.1 | 0.468 |
| **246+** | hsa-miR-760 | −28.2 | 0.540 | **44+A** | hsa-miR-130b-5p | −26.6 | 0.468 |
| **246+** | hsa-miR-935 | −30.6 | 0.540 | **44+A** | hsa-miR-365b-5p | −30.5 | 0.468 |
| **246+** | hsa-miR-718 | −31.5 | 0.540 | **44+A** | hsa-miR-378a-5p | −27.4 | 0.468 |
| **247+** | hsa-miR-937-5p | −29.0 | 1.114 | **44+A** | hsa-miR-338-3p | −28.0 | 0.468 |
| **248+A** | hsa-miR-192-5p | −26.5 | 0.271 | **44+A** | hsa-miR-346 | −28.0 | 0.468 |
| **248+A** | hsa-miR-199b-5p | −26.3 | 0.271 | **44+A** | hsa-miR-412 | −33.7 | 0.468 |
| **248+A** | hsa-miR-211-3p | −26.6 | 0.271 | **44+A** | hsa-miR-492 | −32.9 | 0.468 |
| **248+A** | hsa-miR-511 | −25.7 | 0.271 | **44+A** | hsa-miR-505-3p | −29.7 | 0.468 |
| **248+A** | hsa-miR-566 | −26.7 | 0.271 | **44+A** | hsa-miR-564 | −30.2 | 0.468 |
| **248+A** | hsa-miR-615-5p | −29.3 | 0.271 | **44+A** | hsa-miR-602 | −30.0 | 0.468 |
| **248+A** | hsa-miR-623 | −29.2 | 0.271 | **44+A** | hsa-miR-608 | −32.2 | 0.468 |
| **248+A** | hsa-miR-638 | −33.2 | 0.271 | **44+A** | hsa-miR-623 | −29.5 | 0.468 |
| **248+A** | hsa-miR-671-5p | −29.6 | 0.271 | **44+A** | hsa-miR-767-5p | −35.9 | 0.468 |
| **248+A** | hsa-miR-769-3p | −28.1 | 0.271 | **44+A** | hsa-miR-769-3p | −29.4 | 0.468 |
| **248+A** | hsa-miR-663b | −31.3 | 0.271 | **44+A** | hsa-miR-762 | −29.9 | 0.468 |
| **248+A** | hsa-miR-323b-3p | −28.5 | 0.271 | **44+A** | hsa-miR-770-5p | −29.5 | 0.468 |
| **249+** | hsa-miR-17-3p | −27.0 | 0.697 | **44+A** | hsa-miR-744-5p | −31.5 | 0.468 |
| **249+** | hsa-miR-25-3p | −29.2 | 0.697 | **44+A** | hsa-miR-877-3p | −27.0 | 0.468 |
| **249+** | hsa-miR-30d-3p | −26.3 | 0.697 | **44+A** | hsa-miR-760 | −29.2 | 0.468 |
| **249+** | hsa-miR-767-5p | −28.2 | 0.697 | **44+A** | hsa-miR-378b | −28.8 | 0.468 |
| **252+A** | hsa-miR-377-5p | −26.4 | 1.320 | **44+A** | hsa-miR-378c | −28.1 | 0.468 |
| **252+A** | hsa-miR-505-5p | −26.9 | 1.320 | **44+A** | hsa-miR-378e | −28.6 | 0.468 |
| **252+A** | hsa-miR-671-5p | −32.9 | 1.320 | **44+A** | hsa-miR-378h | −26.5 | 0.468 |
| **253+** | hsa-miR-212-5p | −27.8 | 0.835 | **440+A** | hsa-miR-320a | −28.8 | 0.542 |
| **253+** | hsa-miR-634 | −29.1 | 0.835 | **440+A** | hsa-miR-320b | −28.8 | 0.542 |
| **257+** | hsa-miR-23b-5p | −30.9 | 1.390 | **440+A** | hsa-miR-320c | −27.9 | 0.542 |
| **257+** | hsa-miR-138-5p | −29.3 | 1.390 | **440+A** | hsa-miR-761 | −30.7 | 0.542 |
| **257+** | hsa-miR-645 | −26.6 | 1.390 | **440+A** | hsa-miR-885-3p | −28.8 | 0.542 |
| **261+** | hsa-miR-25-3p | −27.3 | 1.130 | **440+A** | hsa-miR-320d | −26.6 | 0.542 |
| **261+** | hsa-miR-27a-5p | −28.2 | 1.130 | **440+A** | hsa-miR-320e | −26.3 | 0.542 |
| **261+** | hsa-miR-139-5p | −29.7 | 1.130 | **443+A** | hsa-miR-148a-5p | −29.9 | 0.671 |
| **261+** | hsa-miR-432-5p | −31.4 | 1.130 | **443+A** | hsa-miR-147a | −28.4 | 0.671 |
| **261+** | hsa-miR-1264 | −26.9 | 1.130 | **443+A** | hsa-miR-204-3p | −27.5 | 0.671 |
| **261+** | hsa-miR-449c-3p | −30.4 | 1.130 | **443+A** | hsa-miR-646 | −25.7 | 0.671 |
| **261+** | hsa-miR-675-5p | −29.7 | 1.130 | **443+A** | hsa-miR-664b-5p | −29.0 | 0.671 |
| **263+A** | hsa-miR-34a-5p | −26.3 | 0.230 | **445+A** | hsa-miR-216a-5p | −29.4 | 0.319 |
| **263+A** | hsa-miR-323a-5p | −27.9 | 0.230 | **446+** | hsa-miR-324-5p | −28.9 | 0.733 |
| **263+A** | hsa-miR-196b-3p | −26.9 | 0.230 | **446+** | hsa-miR-562 | −26.9 | 0.733 |
| **263+A** | hsa-miR-488-5p | −26.0 | 0.230 | **448+A** | hsa-miR-18a-3p | −29.0 | 0.346 |
| **263+A** | hsa-miR-584-3p | −30.4 | 0.230 | **448+A** | hsa-miR-718 | −30.0 | 0.346 |
| **263+A** | hsa-miR-612 | −31.6 | 0.230 | **448+A** | hsa-miR-514b-3p | −26.9 | 0.346 |
| **263+A** | hsa-miR-614 | −28.1 | 0.230 | **449+A** | hsa-miR-107 | −29.1 | 0.609 |
| **263+A** | hsa-miR-33b-3p | −28.0 | 0.230 | **449+A** | hsa-miR-515-3p | −28.7 | 0.609 |
| **263+A** | hsa-miR-770-5p | −27.8 | 0.230 | **449+A** | hsa-miR-593-5p | −29.3 | 0.609 |
| **263+A** | hsa-miR-885-3p | −31.7 | 0.230 | **449+A** | hsa-miR-614 | −30.9 | 0.609 |
| **263+A** | hsa-miR-940 | −30.9 | 0.230 | **449+A** | hsa-miR-33b-5p | −26.3 | 0.609 |
| **263+A** | hsa-miR-663b | −31.2 | 0.230 | **449+A** | hsa-miR-449c-5p | −28.6 | 0.609 |
| **265+A** | hsa-miR-27a-5p | −28.2 | 0.675 | **450+** | hsa-miR-149-3p | −27.6 | 0.357 |
| **265+A** | hsa-miR-211-3p | −26.5 | 0.675 | **450+** | hsa-miR-296-3p | −29.3 | 0.357 |
| **265+A** | hsa-miR-193a-5p | −31.3 | 0.675 | **450+** | hsa-miR-202-3p | −25.4 | 0.357 |
| **265+A** | hsa-miR-616-3p | −28.2 | 0.675 | **450+** | hsa-miR-519e-3p | −25.8 | 0.357 |
| **265+A** | hsa-miR-766-5p | −27.2 | 0.675 | **450+** | hsa-miR-33b-3p | −30.2 | 0.357 |
| **265+A** | hsa-miR-514b-3p | −26.3 | 0.675 | **450+** | hsa-miR-659-3p | −27.9 | 0.357 |
| **266+A** | hsa-miR-15a-3p | −26.9 | 1.770 | **450+** | hsa-miR-671-3p | −28.3 | 0.357 |
| **266+A** | hsa-miR-125a-3p | −28.7 | 1.770 | **450+** | hsa-miR-675-5p | −30.4 | 0.357 |
| **266+A** | hsa-miR-486-3p | −27.2 | 1.770 | **450+** | hsa-miR-875-3p | −26.4 | 0.357 |
| **266+A** | hsa-miR-503-3p | −28.9 | 1.770 | **450+** | hsa-miR-665 | −30.0 | 0.357 |
| **266+A** | hsa-miR-505-5p | −28.0 | 1.770 | **450+A** | hsa-miR-99a-3p | −26.2 | 0.610 |
| **266+A** | hsa-miR-575 | −25.7 | 1.770 | **450+A** | hsa-miR-99b-3p | −32.5 | 0.610 |
| **266+A** | hsa-miR-637 | −29.5 | 1.770 | **451+** | hsa-miR-135b-3p | −27.8 | 0.906 |
| **266+A** | hsa-miR-638 | −31.5 | 1.770 | **451+** | hsa-miR-575 | −28.4 | 0.906 |
| **266+A** | hsa-miR-769-5p | −27.4 | 1.770 | **451+** | hsa-miR-610 | −27.7 | 0.906 |
| **266+A** | hsa-miR-762 | −30.8 | 1.770 | **452+** | hsa-miR-93-3p | −26.7 | 1.266 |
| **266+A** | hsa-miR-323b-5p | −29.6 | 1.770 | **452+** | hsa-miR-187-5p | −29.3 | 1.266 |
| **266+A** | hsa-miR-892c-5p | −26.3 | 1.770 | **452+** | hsa-miR-216a-5p | −26.6 | 1.266 |
| **268+** | hsa-miR-26b-3p | −26.6 | 0.414 | **452+** | hsa-miR-222-3p | −31.5 | 1.266 |
| **268+** | hsa-miR-328 | −36.0 | 0.414 | **452+** | hsa-miR-367-5p | −26.9 | 1.266 |
| **268+** | hsa-miR-148b-3p | −26.1 | 0.414 | **452+** | hsa-miR-423-3p | −29.0 | 1.266 |
| **268+** | hsa-miR-629-3p | −27.1 | 0.414 | **452+** | hsa-miR-627 | −26.5 | 1.266 |
| **268+** | hsa-miR-767-3p | −29.9 | 0.414 | **452+** | hsa-miR-744-3p | −32.1 | 1.266 |
| **268+** | hsa-miR-874 | −30.1 | 0.414 | **452+** | hsa-miR-885-3p | −32.2 | 1.266 |
| **268+** | hsa-miR-548p | −25.3 | 0.414 | **452+** | hsa-miR-887 | −27.8 | 1.266 |
| **269+** | hsa-let-7e-3p | −29.6 | 0.712 | **452+** | hsa-miR-663b | −28.2 | 1.266 |
| **269+** | hsa-miR-15a-3p | −26.5 | 0.712 | **453+A** | hsa-miR-25-3p | −28.8 | 0.616 |
| **269+** | hsa-miR-23a-3p | −26.4 | 0.712 | **453+A** | hsa-miR-92a-3p | −30.3 | 0.616 |
| **269+** | hsa-miR-30c-2-3p | −29.0 | 0.712 | **453+A** | hsa-miR-211-5p | −31.6 | 0.616 |
| **269+** | hsa-miR-185-3p | −32.9 | 0.712 | **453+A** | hsa-miR-127-5p | −28.4 | 0.616 |
| **269+** | hsa-miR-30c-1-3p | −27.4 | 0.712 | **453+A** | hsa-miR-149-5p | −29.7 | 0.616 |
| **269+** | hsa-miR-377-3p | −25.3 | 0.712 | **453+A** | hsa-miR-185-3p | −28.4 | 0.616 |
| **269+** | hsa-miR-323a-3p | −28.3 | 0.712 | **453+A** | hsa-miR-324-3p | −31.8 | 0.616 |
| **269+** | hsa-miR-324-5p | −27.9 | 0.712 | **453+A** | hsa-miR-432-5p | −29.6 | 0.616 |
| **269+** | hsa-miR-92b-3p | −27.0 | 0.712 | **453+A** | hsa-miR-92b-3p | −29.2 | 0.616 |
| **269+** | hsa-miR-550a-3p | −28.2 | 0.712 | **453+A** | hsa-miR-623 | −30.1 | 0.616 |
| **269+** | hsa-miR-593-3p | −28.3 | 0.712 | **453+A** | hsa-miR-636 | −29.2 | 0.616 |
| **269+** | hsa-miR-615-3p | −37.7 | 0.712 | **453+A** | hsa-miR-637 | −32.2 | 0.616 |
| **269+** | hsa-miR-650 | −30.4 | 0.712 | **453+A** | hsa-miR-639 | −30.1 | 0.616 |
| **269+** | hsa-miR-760 | −30.1 | 0.712 | **453+A** | hsa-miR-762 | −38.9 | 0.616 |
| **269+** | hsa-miR-939-5p | −31.4 | 0.712 | **453+A** | hsa-miR-760 | −29.9 | 0.616 |
| **269+** | hsa-miR-544b | −29.8 | 0.712 | **454+** | hsa-let-7f-5p | −26.4 | 0.750 |
| **269+A** | hsa-miR-27a-5p | −27.0 | 1.817 | **454+** | hsa-miR-147a | −25.8 | 0.750 |
| **269+A** | hsa-miR-211-3p | −27.7 | 1.817 | **454+** | hsa-let-7g-5p | −27.5 | 0.750 |
| **269+A** | hsa-miR-149-3p | −29.2 | 1.817 | **454+** | hsa-miR-185-3p | −28.7 | 0.750 |
| **269+A** | hsa-miR-185-3p | −30.5 | 1.817 | **454+** | hsa-miR-483-5p | −28.4 | 0.750 |
| **269+A** | hsa-miR-365a-5p | −27.8 | 1.817 | **454+** | hsa-miR-608 | −30.3 | 0.750 |
| **269+A** | hsa-miR-365b-5p | −27.8 | 1.817 | **454+** | hsa-miR-648 | −26.1 | 0.750 |
| **269+A** | hsa-miR-638 | −33.5 | 1.817 | **454+** | hsa-miR-663a | −30.4 | 0.750 |
| **269+A** | hsa-miR-660-3p | −26.2 | 1.817 | **454+** | hsa-miR-762 | −29.6 | 0.750 |
| **269+A** | hsa-miR-769-5p | −34.6 | 1.817 | **454+** | hsa-miR-877-5p | −29.1 | 0.750 |
| **269+A** | hsa-miR-769-3p | −31.3 | 1.817 | **456+A** | hsa-miR-31-5p | −27.4 | 0.484 |
| **269+A** | hsa-miR-766-5p | −27.8 | 1.817 | **456+A** | hsa-miR-34a-5p | −29.1 | 0.484 |
| **269+A** | hsa-miR-762 | −33.7 | 1.817 | **456+A** | hsa-miR-200a-5p | −28.7 | 0.484 |
| **269+A** | hsa-miR-937-5p | −29.1 | 1.817 | **456+A** | hsa-miR-193b-5p | −29.2 | 0.484 |
| **269+A** | hsa-miR-939-5p | −37.4 | 1.817 | **456+A** | hsa-miR-512-3p | −28.2 | 0.484 |
| **269+A** | hsa-miR-548q | −27.4 | 1.817 | **456+A** | hsa-miR-564 | −27.4 | 0.484 |
| **274+** | hsa-miR-197-5p | −32.6 | 0.908 | **456+A** | hsa-miR-541-3p | −28.3 | 0.484 |
| **274+** | hsa-miR-34a-5p | −28.9 | 0.908 | **456+A** | hsa-miR-939-5p | −30.8 | 0.484 |
| **274+** | hsa-miR-34b-5p | −28.7 | 0.908 | **457+** | hsa-miR-127-5p | −28.4 | 0.434 |
| **274+** | hsa-miR-34c-5p | −32.0 | 0.908 | **457+** | hsa-miR-523-3p | −30.3 | 0.434 |
| **274+** | hsa-miR-328 | −32.5 | 0.908 | **457+** | hsa-miR-518a-5p | −26.1 | 0.434 |
| **274+** | hsa-miR-608 | −30.8 | 0.908 | **457+** | hsa-miR-527 | −26.1 | 0.434 |
| **274+** | hsa-miR-449b-5p | −27.2 | 0.908 | **457+** | hsa-miR-578 | −26.5 | 0.434 |
| **274+** | hsa-miR-449c-5p | −37.3 | 0.908 | **457+** | hsa-miR-596 | −28.9 | 0.434 |
| **274+** | hsa-miR-920 | −27.2 | 0.908 | **457+** | hsa-miR-635 | −27.7 | 0.434 |
| **274+** | hsa-miR-663b | −31.5 | 0.908 | **457+** | hsa-miR-658 | −29.0 | 0.434 |
| **274+A** | hsa-miR-124-5p | −29.9 | 0.908 | **457+** | hsa-miR-744-5p | −27.6 | 0.434 |
| **274+A** | hsa-miR-646 | −26.6 | 0.908 | **458+** | hsa-let-7a-5p | −27.3 | 0.798 |
| **274+A** | hsa-miR-449c-5p | −30.3 | 0.908 | **458+** | hsa-let-7b-5p | −28.3 | 0.798 |
| **275+A** | hsa-miR-125b-1-3p | −27.8 | 0.458 | **458+** | hsa-let-7c | −27.6 | 0.798 |
| **275+A** | hsa-miR-128 | −29.3 | 0.458 | **458+** | hsa-let-7d-5p | −28.1 | 0.798 |
| **275+A** | hsa-miR-526b-5p | −27.2 | 0.458 | **458+** | hsa-miR-93-3p | −27.6 | 0.798 |
| **275+A** | hsa-miR-767-5p | −27.7 | 0.458 | **458+** | hsa-miR-217 | −28.4 | 0.798 |
| **275+A** | hsa-miR-548w | −26.7 | 0.458 | **458+** | hsa-let-7g-5p | −25.9 | 0.798 |
| **276+** | hsa-miR-370 | −30.4 | 0.868 | **458+** | hsa-let-7i-5p | −26.6 | 0.798 |
| **276+** | hsa-miR-526b-3p | −28.1 | 0.868 | **458+** | hsa-miR-150-3p | −30.3 | 0.798 |
| **276+** | hsa-miR-505-5p | −33.6 | 0.868 | **458+** | hsa-miR-485-3p | −29.5 | 0.798 |
| **276+** | hsa-miR-658 | −33.0 | 0.868 | **458+** | hsa-miR-202-3p | −28.5 | 0.798 |
| **276+** | hsa-miR-939-5p | −31.5 | 0.868 | **458+** | hsa-miR-509-3p | −26.6 | 0.798 |
| **276+** | hsa-miR-378g | −31.2 | 0.868 | **458+** | hsa-miR-455-3p | −26.1 | 0.798 |
| **276+A** | hsa-miR-19b-2-5p | −27.4 | 0.868 | **458+** | hsa-miR-657 | −30.0 | 0.798 |
| **276+A** | hsa-miR-199a-3p | −28.4 | 0.868 | **458+** | hsa-miR-671-5p | −30.7 | 0.798 |
| **276+A** | hsa-miR-199b-3p | −28.4 | 0.868 | **458+** | hsa-miR-892b | −32.3 | 0.798 |
| **276+A** | hsa-miR-149-5p | −30.9 | 0.868 | **458+** | hsa-miR-541-3p | −27.0 | 0.798 |
| **276+A** | hsa-miR-331-3p | −30.5 | 0.868 | **458+** | hsa-miR-744-5p | −27.6 | 0.798 |
| **276+A** | hsa-miR-612 | −34.1 | 0.868 | **458+** | hsa-miR-544b | −26.6 | 0.798 |
| **276+A** | hsa-miR-767-5p | −33.9 | 0.868 | **458+A** | hsa-miR-25-5p | −26.0 | 1.274 |
| **276+A** | hsa-miR-762 | −32.6 | 0.868 | **458+A** | hsa-miR-27a-5p | −28.1 | 1.274 |
| **276+A** | hsa-miR-374b-3p | −28.3 | 0.868 | **458+A** | hsa-miR-197-5p | −30.2 | 1.274 |
| **278+** | hsa-miR-22-3p | −26.6 | 1.999 | **458+A** | hsa-miR-211-3p | −26.4 | 1.274 |
| **278+** | hsa-miR-135a-3p | −27.6 | 1.999 | **458+A** | hsa-miR-373-3p | −26.3 | 1.274 |
| **278+** | hsa-miR-34b-5p | −26.8 | 1.999 | **458+A** | hsa-miR-342-5p | −28.5 | 1.274 |
| **278+** | hsa-miR-296-3p | −32.3 | 1.999 | **458+A** | hsa-miR-148b-5p | −25.3 | 1.274 |
| **278+** | hsa-miR-370 | −29.8 | 1.999 | **458+A** | hsa-miR-339-5p | −28.2 | 1.274 |
| **278+** | hsa-miR-493-5p | −28.8 | 1.999 | **458+A** | hsa-miR-146b-3p | −30.0 | 1.274 |
| **278+** | hsa-miR-193b-5p | −29.5 | 1.999 | **458+A** | hsa-miR-532-5p | −26.4 | 1.274 |
| **278+** | hsa-miR-449c-5p | −28.6 | 1.999 | **458+A** | hsa-miR-574-5p | −29.5 | 1.274 |
| **278+** | hsa-miR-762 | −32.4 | 1.999 | **458+A** | hsa-miR-541-3p | −26.7 | 1.274 |
| **278+** | hsa-miR-937-5p | −27.5 | 1.999 | **462+A** | hsa-miR-103a-2-5p | −30.2 | 0.353 |
| **278+** | hsa-miR-939-5p | −31.5 | 1.999 | **462+A** | hsa-miR-149-5p | −34.7 | 0.353 |
| **28+** | hsa-miR-330-5p | −28.6 | 0.461 | **462+A** | hsa-miR-483-3p | −31.9 | 0.353 |
| **28+** | hsa-miR-659-3p | −29.1 | 0.461 | **462+A** | hsa-miR-636 | −37.8 | 0.353 |
| **281+** | hsa-miR-197-5p | −33.3 | 0.679 | **462+A** | hsa-miR-637 | −36.1 | 0.353 |
| **281+** | hsa-miR-211-3p | −27.7 | 0.679 | **465+** | hsa-miR-197-5p | −34.9 | 0.747 |
| **281+** | hsa-miR-27b-5p | −26.8 | 0.679 | **465+** | hsa-miR-34a-5p | −34.0 | 0.747 |
| **281+** | hsa-miR-432-5p | −27.4 | 0.679 | **465+** | hsa-miR-211-3p | −34.6 | 0.747 |
| **281+** | hsa-miR-505-5p | −28.0 | 0.679 | **465+** | hsa-miR-138-5p | −29.8 | 0.747 |
| **281+** | hsa-miR-575 | −27.0 | 0.679 | **465+** | hsa-miR-149-3p | −34.3 | 0.747 |
| **281+** | hsa-miR-638 | −31.2 | 0.679 | **465+** | hsa-miR-449a | −29.6 | 0.747 |
| **281+** | hsa-miR-658 | −30.1 | 0.679 | **465+** | hsa-miR-431-3p | −28.5 | 0.747 |
| **281+** | hsa-miR-762 | −30.3 | 0.679 | **465+** | hsa-miR-92b-5p | −32.0 | 0.747 |
| **281+** | hsa-miR-760 | −28.8 | 0.679 | **465+** | hsa-miR-663a | −31.9 | 0.747 |
| **281+** | hsa-miR-920 | −26.8 | 0.679 | **465+** | hsa-miR-449b-5p | −28.2 | 0.747 |
| **282+A** | hsa-miR-363-5p | −28.0 | 3.605 | **465+** | hsa-miR-659-3p | −31.9 | 0.747 |
| **282+A** | hsa-miR-323a-5p | −29.0 | 3.605 | **466+A** | hsa-miR-512-3p | −29.5 | 2.094 |
| **282+A** | hsa-miR-499a-3p | −26.4 | 3.605 | **468+** | hsa-let-7b-5p | −28.7 | 0.376 |
| **282+A** | hsa-miR-564 | −26.0 | 3.605 | **468+** | hsa-miR-196a-5p | −29.8 | 0.376 |
| **283+** | hsa-miR-92a-3p | −28.5 | 1.174 | **468+** | hsa-miR-302d-5p | −28.8 | 0.376 |
| **283+** | hsa-miR-103a-2-5p | −27.1 | 1.174 | **468+** | hsa-miR-383 | −28.7 | 0.376 |
| **283+** | hsa-miR-302a-3p | −28.9 | 1.174 | **468+** | hsa-miR-196b-5p | −28.9 | 0.376 |
| **283+** | hsa-miR-302d-3p | −27.2 | 1.174 | **468+** | hsa-miR-503-5p | −30.1 | 0.376 |
| **283+** | hsa-miR-498 | −30.1 | 1.174 | **468+** | hsa-miR-550a-5p | −33.9 | 0.376 |
| **283+** | hsa-miR-520f | −28.5 | 1.174 | **468+** | hsa-miR-650 | −30.6 | 0.376 |
| **283+** | hsa-miR-520a-3p | −30.9 | 1.174 | **468+** | hsa-miR-550a-3-5p | −30.2 | 0.376 |
| **283+** | hsa-miR-520b | −26.1 | 1.174 | **468+** | hsa-miR-766-5p | −31.4 | 0.376 |
| **283+** | hsa-miR-520c-3p | −28.4 | 1.174 | **469+A** | hsa-miR-93-3p | −27.0 | 0.327 |
| **283+** | hsa-miR-518c-5p | −30.3 | 1.174 | **469+A** | hsa-miR-181a-2-3p | −27.2 | 0.327 |
| **283+** | hsa-miR-520d-3p | −32.8 | 1.174 | **469+A** | hsa-miR-182-3p | −27.7 | 0.327 |
| **283+** | hsa-miR-92b-3p | −31.8 | 1.174 | **469+A** | hsa-miR-296-5p | −29.1 | 0.327 |
| **283+** | hsa-miR-596 | −29.7 | 1.174 | **469+A** | hsa-miR-330-5p | −30.3 | 0.327 |
| **283+** | hsa-miR-665 | −36.0 | 1.174 | **469+A** | hsa-miR-328 | −30.2 | 0.327 |
| **283+** | hsa-miR-937-5p | −29.6 | 1.174 | **469+A** | hsa-miR-326 | −31.7 | 0.327 |
| **283+** | hsa-miR-663b | −28.9 | 1.174 | **469+A** | hsa-miR-526b-5p | −28.9 | 0.327 |
| **283+** | hsa-miR-302e | −25.4 | 1.174 | **469+A** | hsa-miR-612 | −28.9 | 0.327 |
| **283+A** | hsa-miR-211-3p | −29.4 | 3.391 | **469+A** | hsa-miR-637 | −29.8 | 0.327 |
| **283+A** | hsa-miR-149-3p | −36.7 | 3.391 | **469+A** | hsa-miR-499b-3p | −28.2 | 0.327 |
| **283+A** | hsa-miR-185-3p | −29.0 | 3.391 | **47+** | hsa-miR-221-5p | −26.7 | 0.521 |
| **283+A** | hsa-miR-455-5p | −26.6 | 3.391 | **47+** | hsa-miR-125a-3p | −29.4 | 0.521 |
| **283+A** | hsa-miR-640 | −30.2 | 3.391 | **47+** | hsa-miR-328 | −28.7 | 0.521 |
| **283+A** | hsa-miR-671-5p | −30.2 | 3.391 | **47+** | hsa-miR-519c-5p | −30.0 | 0.521 |
| **283+A** | hsa-miR-937-3p | −28.6 | 3.391 | **47+** | hsa-miR-519b-5p | −30.0 | 0.521 |
| **283+A** | hsa-miR-939-5p | −34.2 | 3.391 | **47+** | hsa-miR-523-5p | −30.0 | 0.521 |
| **285+** | hsa-miR-19b-1-5p | −26.7 | 0.553 | **47+** | hsa-miR-518f-5p | −31.4 | 0.521 |
| **285+** | hsa-miR-105-3p | −31.0 | 0.553 | **47+** | hsa-miR-526a | −30.4 | 0.521 |
| **285+** | hsa-miR-34a-5p | −27.1 | 0.553 | **47+** | hsa-miR-520c-5p | −30.4 | 0.521 |
| **285+** | hsa-miR-181c-3p | −28.5 | 0.553 | **47+** | hsa-miR-518e-5p | −30.0 | 0.521 |
| **285+** | hsa-miR-211-3p | −26.8 | 0.553 | **47+** | hsa-miR-518d-5p | −30.4 | 0.521 |
| **285+** | hsa-miR-140-5p | −26.6 | 0.553 | **47+** | hsa-miR-522-5p | −30.0 | 0.521 |
| **285+** | hsa-miR-149-3p | −27.6 | 0.553 | **47+** | hsa-miR-519a-5p | −30.0 | 0.521 |
| **285+** | hsa-miR-365b-5p | −28.3 | 0.553 | **47+** | hsa-miR-608 | −29.8 | 0.521 |
| **285+** | hsa-miR-370 | −32.7 | 0.553 | **47+** | hsa-miR-33b-3p | −30.1 | 0.521 |
| **285+** | hsa-miR-345-3p | −35.3 | 0.553 | **47+** | hsa-miR-638 | −33.6 | 0.521 |
| **285+** | hsa-miR-423-3p | −29.4 | 0.553 | **47+** | hsa-miR-933 | −31.5 | 0.521 |
| **285+** | hsa-miR-431-5p | −27.1 | 0.553 | **47+** | hsa-miR-103b | −28.2 | 0.521 |
| **285+** | hsa-miR-491-5p | −38.1 | 0.553 | **47+** | hsa-miR-548aq-5p | −26.3 | 0.521 |
| **285+** | hsa-miR-146b-3p | −31.5 | 0.553 | **471+A** | hsa-miR-29b-3p | −26.8 | 0.819 |
| **285+** | hsa-miR-526b-5p | −26.8 | 0.553 | **471+A** | hsa-miR-648 | −25.6 | 0.819 |
| **285+** | hsa-miR-516a-5p | −27.8 | 0.553 | **472+A** | hsa-miR-103b | −28.3 | 0.822 |
| **285+** | hsa-miR-502-5p | −26.1 | 0.553 | **473+A** | hsa-miR-17-3p | −27.7 | 0.271 |
| **285+** | hsa-miR-503-3p | −29.2 | 0.553 | **473+A** | hsa-miR-23a-5p | −31.4 | 0.271 |
| **285+** | hsa-miR-513a-5p | −25.3 | 0.553 | **473+A** | hsa-miR-24-3p | −27.4 | 0.271 |
| **285+** | hsa-miR-572 | −31.7 | 0.553 | **473+A** | hsa-miR-27a-5p | −29.2 | 0.271 |
| **285+** | hsa-miR-608 | −29.3 | 0.553 | **473+A** | hsa-miR-33a-5p | −26.5 | 0.271 |
| **285+** | hsa-miR-623 | −33.0 | 0.553 | **473+A** | hsa-miR-93-3p | −28.7 | 0.271 |
| **285+** | hsa-miR-671-5p | −32.9 | 0.553 | **473+A** | hsa-miR-187-3p | −28.5 | 0.271 |
| **285+** | hsa-miR-874 | −30.7 | 0.553 | **473+A** | hsa-miR-214-3p | −28.2 | 0.271 |
| **285+** | hsa-miR-920 | −28.3 | 0.553 | **473+A** | hsa-miR-191-3p | −32.3 | 0.271 |
| **285+** | hsa-miR-939-5p | −31.2 | 0.553 | **473+A** | hsa-miR-149-5p | −33.4 | 0.271 |
| **285+** | hsa-miR-513b | −25.6 | 0.553 | **473+A** | hsa-miR-149-3p | −33.4 | 0.271 |
| **285+** | hsa-miR-544b | −26.0 | 0.553 | **473+A** | hsa-miR-185-3p | −27.7 | 0.271 |
| **287+A** | hsa-miR-20a-3p | −26.0 | 0.254 | **473+A** | hsa-miR-193a-5p | −28.5 | 0.271 |
| **287+A** | hsa-miR-331-5p | −28.7 | 0.254 | **473+A** | hsa-miR-195-5p | −25.1 | 0.271 |
| **287+A** | hsa-miR-483-3p | −31.0 | 0.254 | **473+A** | hsa-miR-365a-5p | −27.9 | 0.271 |
| **288+A** | hsa-miR-125b-5p | −28.0 | 0.447 | **473+A** | hsa-miR-365b-5p | −27.4 | 0.271 |
| **288+A** | hsa-miR-125a-5p | −29.8 | 0.447 | **473+A** | hsa-miR-328 | −29.3 | 0.271 |
| **288+A** | hsa-miR-136-3p | −27.4 | 0.447 | **473+A** | hsa-miR-331-3p | −27.2 | 0.271 |
| **289+** | hsa-miR-921 | −29.0 | 0.810 | **473+A** | hsa-miR-431-5p | −28.2 | 0.271 |
| **289+A** | hsa-miR-639 | −28.4 | 0.847 | **473+A** | hsa-miR-486-3p | −27.3 | 0.271 |
| **291+** | hsa-miR-424-3p | −30.3 | 1.142 | **473+A** | hsa-miR-146b-3p | −28.6 | 0.271 |
| **291+** | hsa-miR-670 | −29.6 | 1.142 | **473+A** | hsa-miR-502-5p | −28.6 | 0.271 |
| **292+** | hsa-miR-23a-5p | −30.1 | 0.630 | **473+A** | hsa-miR-554 | −28.2 | 0.271 |
| **292+** | hsa-miR-211-3p | −28.7 | 0.630 | **473+A** | hsa-miR-602 | −31.3 | 0.271 |
| **292+** | hsa-miR-127-5p | −27.0 | 0.630 | **473+A** | hsa-miR-621 | −27.6 | 0.271 |
| **292+** | hsa-miR-149-3p | −27.9 | 0.630 | **473+A** | hsa-miR-33b-5p | −28.1 | 0.271 |
| **292+** | hsa-miR-185-3p | −27.6 | 0.630 | **473+A** | hsa-miR-637 | −30.5 | 0.271 |
| **292+** | hsa-miR-423-3p | −28.9 | 0.630 | **473+A** | hsa-miR-638 | −32.4 | 0.271 |
| **292+** | hsa-miR-485-5p | −27.3 | 0.630 | **473+A** | hsa-miR-639 | −29.8 | 0.271 |
| **292+** | hsa-miR-193b-5p | −30.4 | 0.630 | **473+A** | hsa-miR-644a | −25.0 | 0.271 |
| **292+** | hsa-miR-612 | −28.4 | 0.630 | **473+A** | hsa-miR-767-5p | −28.1 | 0.271 |
| **292+** | hsa-miR-650 | −26.9 | 0.630 | **473+A** | hsa-miR-874 | −33.2 | 0.271 |
| **292+** | hsa-miR-671-5p | −30.7 | 0.630 | **473+A** | hsa-miR-744-5p | −30.9 | 0.271 |
| **292+** | hsa-miR-769-3p | −28.1 | 0.630 | **473+A** | hsa-miR-939-5p | −31.8 | 0.271 |
| **292+** | hsa-miR-762 | −28.8 | 0.630 | **473+A** | hsa-miR-663b | −33.1 | 0.271 |
| **292+** | hsa-miR-885-3p | −30.8 | 0.630 | **473+A** | hsa-miR-378j | −25.8 | 0.271 |
| **292+** | hsa-miR-877-5p | −29.5 | 0.630 | **476+** | hsa-miR-103a-2-5p | −26.8 | 0.735 |
| **295+A** | hsa-miR-29b-1-5p | −26.4 | 0.848 | **476+** | hsa-miR-198 | −29.5 | 0.735 |
| **295+A** | hsa-miR-204-5p | −29.1 | 0.848 | **476+** | hsa-miR-185-5p | −27.0 | 0.735 |
| **295+A** | hsa-miR-211-5p | −27.9 | 0.848 | **476+** | hsa-miR-328 | −32.3 | 0.735 |
| **295+A** | hsa-miR-330-5p | −28.8 | 0.848 | **476+** | hsa-miR-622 | −27.7 | 0.735 |
| **295+A** | hsa-miR-326 | −26.4 | 0.848 | **476+** | hsa-miR-658 | −30.9 | 0.735 |
| **295+A** | hsa-miR-331-3p | −29.1 | 0.848 | **476+** | hsa-miR-921 | −30.3 | 0.735 |
| **295+A** | hsa-miR-522-3p | −25.8 | 0.848 | **476+** | hsa-miR-936 | −27.2 | 0.735 |
| **295+A** | hsa-miR-609 | −28.0 | 0.848 | **476+A** | hsa-miR-27a-5p | −27.9 | 0.735 |
| **295+A** | hsa-miR-613 | −24.8 | 0.848 | **476+A** | hsa-miR-218-2-3p | −27.5 | 0.735 |
| **295+A** | hsa-miR-642b-5p | −26.4 | 0.848 | **476+A** | hsa-miR-149-3p | −30.5 | 0.735 |
| **296+** | hsa-let-7c | −28.1 | 0.670 | **476+A** | hsa-miR-185-3p | −31.4 | 0.735 |
| **296+** | hsa-miR-24-2-5p | −30.7 | 0.670 | **476+A** | hsa-miR-423-5p | −29.1 | 0.735 |
| **296+** | hsa-let-7i-3p | −29.5 | 0.670 | **476+A** | hsa-miR-623 | −27.9 | 0.735 |
| **296+** | hsa-miR-365b-5p | −31.5 | 0.670 | **476+A** | hsa-miR-762 | −29.5 | 0.735 |
| **296+** | hsa-miR-324-3p | −31.8 | 0.670 | **477+A** | hsa-miR-103a-2-5p | −27.2 | 0.298 |
| **296+** | hsa-miR-513a-5p | −28.2 | 0.670 | **477+A** | hsa-miR-211-5p | −27.2 | 0.298 |
| **296+** | hsa-miR-574-5p | −35.6 | 0.670 | **477+A** | hsa-miR-133a | −30.7 | 0.298 |
| **296+** | hsa-miR-574-3p | −30.5 | 0.670 | **477+A** | hsa-miR-149-5p | −30.4 | 0.298 |
| **296+** | hsa-miR-636 | −33.2 | 0.670 | **477+A** | hsa-miR-150-5p | −32.1 | 0.298 |
| **296+** | hsa-miR-637 | −32.8 | 0.670 | **477+A** | hsa-miR-185-3p | −29.7 | 0.298 |
| **296+** | hsa-miR-639 | −32.8 | 0.670 | **477+A** | hsa-miR-193a-5p | −28.1 | 0.298 |
| **296+** | hsa-miR-675-5p | −33.2 | 0.670 | **477+A** | hsa-miR-296-5p | −30.7 | 0.298 |
| **298+** | hsa-miR-22-5p | −27.4 | 0.454 | **477+A** | hsa-miR-370 | −27.3 | 0.298 |
| **298+** | hsa-miR-34a-5p | −28.3 | 0.454 | **477+A** | hsa-miR-330-5p | −28.3 | 0.298 |
| **298+** | hsa-miR-412 | −32.4 | 0.454 | **477+A** | hsa-miR-328 | −38.2 | 0.298 |
| **298+** | hsa-miR-517-5p | −27.9 | 0.454 | **477+A** | hsa-miR-323a-5p | −27.5 | 0.298 |
| **298+** | hsa-miR-671-5p | −34.5 | 0.454 | **477+A** | hsa-miR-326 | −35.6 | 0.298 |
| **298+** | hsa-miR-939-5p | −35.3 | 0.454 | **477+A** | hsa-miR-331-3p | −27.8 | 0.298 |
| **299+A** | hsa-let-7a-2-3p | −26.5 | 0.563 | **477+A** | hsa-miR-133b | −30.7 | 0.298 |
| **299+A** | hsa-miR-27a-3p | −26.9 | 0.563 | **477+A** | hsa-miR-425-3p | −27.3 | 0.298 |
| **299+A** | hsa-miR-92a-2-5p | −26.2 | 0.563 | **477+A** | hsa-miR-488-5p | −32.8 | 0.298 |
| **299+A** | hsa-miR-30c-2-3p | −32.4 | 0.563 | **477+A** | hsa-miR-532-3p | −27.9 | 0.298 |
| **299+A** | hsa-miR-30d-5p | −27.6 | 0.563 | **477+A** | hsa-miR-572 | −27.7 | 0.298 |
| **299+A** | hsa-miR-204-5p | −30.1 | 0.563 | **477+A** | hsa-miR-623 | −27.6 | 0.298 |
| **299+A** | hsa-miR-204-3p | −29.3 | 0.563 | **477+A** | hsa-miR-637 | −30.7 | 0.298 |
| **299+A** | hsa-miR-211-5p | −27.6 | 0.563 | **477+A** | hsa-miR-642a-5p | −28.0 | 0.298 |
| **299+A** | hsa-miR-211-3p | −32.4 | 0.563 | **477+A** | hsa-miR-659-3p | −28.5 | 0.298 |
| **299+A** | hsa-miR-212-5p | −27.0 | 0.563 | **477+A** | hsa-miR-877-3p | −28.5 | 0.298 |
| **299+A** | hsa-miR-214-3p | −27.5 | 0.563 | **477+A** | hsa-miR-940 | −29.6 | 0.298 |
| **299+A** | hsa-let-7g-3p | −30.4 | 0.563 | **477+A** | hsa-miR-942 | −29.3 | 0.298 |
| **299+A** | hsa-let-7i-3p | −29.7 | 0.563 | **477+A** | hsa-miR-718 | −30.8 | 0.298 |
| **299+A** | hsa-miR-27b-3p | −25.4 | 0.563 | **479+** | hsa-let-7b-5p | −27.4 | 1.158 |
| **299+A** | hsa-miR-125b-5p | −29.7 | 0.563 | **479+** | hsa-let-7d-5p | −27.4 | 1.158 |
| **299+A** | hsa-miR-130a-5p | −26.0 | 0.563 | **479+** | hsa-miR-27a-5p | −28.9 | 1.158 |
| **299+A** | hsa-miR-132-5p | −26.5 | 0.563 | **479+** | hsa-miR-214-5p | −27.9 | 1.158 |
| **299+A** | hsa-miR-9-5p | −26.4 | 0.563 | **479+** | hsa-miR-296-5p | −29.2 | 1.158 |
| **299+A** | hsa-miR-125a-5p | −32.4 | 0.563 | **479+** | hsa-miR-566 | −26.7 | 1.158 |
| **299+A** | hsa-miR-146a-3p | −25.5 | 0.563 | **479+** | hsa-miR-614 | −32.6 | 1.158 |
| **299+A** | hsa-miR-149-5p | −27.7 | 0.563 | **479+** | hsa-miR-615-5p | −29.9 | 1.158 |
| **299+A** | hsa-miR-149-3p | −31.6 | 0.563 | **479+** | hsa-miR-662 | −29.1 | 1.158 |
| **299+A** | hsa-miR-185-3p | −38.2 | 0.563 | **479+** | hsa-miR-762 | −31.6 | 1.158 |
| **299+A** | hsa-miR-188-5p | −31.3 | 0.563 | **479+** | hsa-miR-760 | −29.5 | 1.158 |
| **299+A** | hsa-miR-188-3p | −30.9 | 0.563 | **479+** | hsa-miR-939-5p | −38.8 | 1.158 |
| **299+A** | hsa-miR-106b-3p | −29.2 | 0.563 | **479+** | hsa-miR-663b | −30.6 | 1.158 |
| **299+A** | hsa-miR-30c-1-3p | −32.4 | 0.563 | **479+** | hsa-miR-548ag | −26.1 | 1.158 |
| **299+A** | hsa-miR-296-3p | −30.9 | 0.563 | **48+A** | hsa-miR-26a-5p | −27.8 | 0.374 |
| **299+A** | hsa-miR-370 | −31.3 | 0.563 | **48+A** | hsa-miR-425-3p | −29.8 | 0.374 |
| **299+A** | hsa-miR-378a-5p | −31.1 | 0.563 | **48+A** | hsa-miR-490-5p | −27.5 | 0.374 |
| **299+A** | hsa-miR-380-5p | −26.2 | 0.563 | **48+A** | hsa-miR-503-3p | −29.0 | 0.374 |
| **299+A** | hsa-miR-330-5p | −27.1 | 0.563 | **48+A** | hsa-miR-567 | −27.4 | 0.374 |
| **299+A** | hsa-miR-326 | −27.3 | 0.563 | **48+A** | hsa-miR-593-3p | −27.5 | 0.374 |
| **299+A** | hsa-miR-331-3p | −27.5 | 0.563 | **48+A** | hsa-miR-598 | −33.4 | 0.374 |
| **299+A** | hsa-miR-324-5p | −31.6 | 0.563 | **48+A** | hsa-miR-637 | −29.4 | 0.374 |
| **299+A** | hsa-miR-345-5p | −32.1 | 0.563 | **48+A** | hsa-miR-769-3p | −30.2 | 0.374 |
| **299+A** | hsa-miR-425-3p | −29.1 | 0.563 | **48+A** | hsa-miR-877-3p | −28.9 | 0.374 |
| **299+A** | hsa-miR-450a-5p | −25.5 | 0.563 | **48+A** | hsa-miR-513b | −26.4 | 0.374 |
| **299+A** | hsa-miR-433 | −29.1 | 0.563 | **482+A** | hsa-miR-539-5p | −28.7 | 1.239 |
| **299+A** | hsa-miR-412 | −28.5 | 0.563 | **5+A** | hsa-miR-657 | −28.6 | 0.784 |
| **299+A** | hsa-miR-483-3p | −28.4 | 0.563 | **54+** | hsa-miR-17-5p | −28.2 | 1.219 |
| **299+A** | hsa-miR-489 | −29.5 | 0.563 | **54+** | hsa-miR-19b-1-5p | −33.1 | 1.219 |
| **299+A** | hsa-miR-491-5p | −27.0 | 0.563 | **54+** | hsa-miR-19b-2-5p | −30.1 | 1.219 |
| **299+A** | hsa-miR-146b-3p | −30.6 | 0.563 | **54+** | hsa-miR-20a-5p | −26.8 | 1.219 |
| **299+A** | hsa-miR-493-5p | −25.8 | 0.563 | **54+** | hsa-miR-27a-5p | −26.8 | 1.219 |
| **299+A** | hsa-miR-526b-5p | −27.5 | 0.563 | **54+** | hsa-miR-31-5p | −27.3 | 1.219 |
| **299+A** | hsa-miR-518e-3p | −26.9 | 0.563 | **54+** | hsa-miR-93-5p | −31.3 | 1.219 |
| **299+A** | hsa-miR-501-5p | −30.5 | 0.563 | **54+** | hsa-miR-106a-5p | −26.0 | 1.219 |
| **299+A** | hsa-miR-584-3p | −28.6 | 0.563 | **54+** | hsa-miR-128 | −25.7 | 1.219 |
| **299+A** | hsa-miR-550a-5p | −30.6 | 0.563 | **54+** | hsa-miR-20b-5p | −28.2 | 1.219 |
| **299+A** | hsa-miR-608 | −30.1 | 0.563 | **54+** | hsa-miR-490-3p | −30.0 | 1.219 |
| **299+A** | hsa-miR-612 | −29.0 | 0.563 | **54+** | hsa-miR-146b-3p | −28.0 | 1.219 |
| **299+A** | hsa-miR-615-3p | −31.7 | 0.563 | **54+** | hsa-miR-516b-5p | −26.3 | 1.219 |
| **299+A** | hsa-miR-621 | −30.5 | 0.563 | **54+** | hsa-miR-572 | −31.2 | 1.219 |
| **299+A** | hsa-miR-623 | −27.6 | 0.563 | **54+** | hsa-miR-600 | −28.4 | 1.219 |
| **299+A** | hsa-miR-626 | −24.2 | 0.563 | **54+** | hsa-miR-637 | −28.8 | 1.219 |
| **299+A** | hsa-miR-642a-5p | −26.8 | 0.563 | **54+** | hsa-miR-638 | −29.9 | 1.219 |
| **299+A** | hsa-miR-647 | −28.3 | 0.563 | **54+** | hsa-miR-449c-5p | −28.3 | 1.219 |
| **299+A** | hsa-miR-663a | −29.7 | 0.563 | **54+** | hsa-miR-762 | −33.9 | 1.219 |
| **299+A** | hsa-miR-654-5p | −35.4 | 0.563 | **54+** | hsa-miR-744-5p | −26.9 | 1.219 |
| **299+A** | hsa-miR-671-3p | −27.4 | 0.563 | **54+** | hsa-miR-939-5p | −34.6 | 1.219 |
| **299+A** | hsa-miR-550a-3-5p | −27.6 | 0.563 | **56+** | hsa-miR-29a-5p | −27.7 | 1.154 |
| **299+A** | hsa-miR-762 | −35.4 | 0.563 | **56+** | hsa-miR-204-3p | −26.9 | 1.154 |
| **299+A** | hsa-miR-675-3p | −27.0 | 0.563 | **56+** | hsa-miR-30b-3p | −26.4 | 1.154 |
| **299+A** | hsa-miR-874 | −30.0 | 0.563 | **56+** | hsa-miR-149-3p | −34.0 | 1.154 |
| **299+A** | hsa-miR-541-3p | −30.3 | 0.563 | **56+** | hsa-miR-185-3p | −27.6 | 1.154 |
| **299+A** | hsa-miR-744-5p | −29.4 | 0.563 | **56+** | hsa-miR-30c-1-3p | −28.8 | 1.154 |
| **299+A** | hsa-miR-939-3p | −29.8 | 0.563 | **56+** | hsa-miR-485-5p | −26.2 | 1.154 |
| **299+A** | hsa-miR-323b-5p | −28.8 | 0.563 | **56+** | hsa-miR-193b-3p | −26.6 | 1.154 |
| **299+A** | hsa-miR-550b-2-5p | −30.2 | 0.563 | **56+** | hsa-miR-519b-3p | −26.4 | 1.154 |
| **3+** | hsa-let-7a-2-3p | −26.5 | 0.366 | **56+** | hsa-miR-608 | −33.2 | 1.154 |
| **3+** | hsa-miR-139-3p | −29.4 | 0.366 | **56+** | hsa-miR-623 | −28.6 | 1.154 |
| **3+** | hsa-miR-125b-1-3p | −28.1 | 0.366 | **56+** | hsa-miR-762 | −30.3 | 1.154 |
| **3+** | hsa-miR-193b-5p | −29.8 | 0.366 | **56+** | hsa-miR-663b | −28.8 | 1.154 |
| **3+** | hsa-miR-623 | −29.7 | 0.366 | **57+** | hsa-miR-19b-2-5p | −26.0 | 0.809 |
| **3+** | hsa-miR-760 | −26.7 | 0.366 | **57+** | hsa-miR-34a-5p | −27.8 | 0.809 |
| **302+** | hsa-miR-484 | −33.2 | 0.793 | **57+** | hsa-miR-143-5p | −28.1 | 0.809 |
| **304+A** | hsa-miR-27b-5p | −27.4 | 1.300 | **57+** | hsa-miR-376a-2-5p | −26.4 | 0.809 |
| **304+A** | hsa-miR-193a-5p | −27.7 | 1.300 | **57+** | hsa-miR-575 | −26.8 | 0.809 |
| **304+A** | hsa-miR-363-5p | −27.6 | 1.300 | **57+** | hsa-miR-616-3p | −30.6 | 0.809 |
| **304+A** | hsa-miR-378a-5p | −28.5 | 1.300 | **57+** | hsa-miR-626 | −26.7 | 0.809 |
| **304+A** | hsa-miR-193b-5p | −28.3 | 1.300 | **57+** | hsa-miR-33b-3p | −29.9 | 0.809 |
| **304+A** | hsa-miR-532-5p | −29.7 | 1.300 | **57+** | hsa-miR-639 | −28.5 | 0.809 |
| **304+A** | hsa-miR-636 | −28.9 | 1.300 | **57+** | hsa-miR-762 | −30.2 | 0.809 |
| **304+A** | hsa-miR-663a | −34.7 | 1.300 | **57+** | hsa-miR-937-5p | −29.0 | 0.809 |
| **304+A** | hsa-miR-659-3p | −28.3 | 1.300 | **57+** | hsa-miR-664b-5p | −30.6 | 0.809 |
| **304+A** | hsa-miR-668 | −32.2 | 1.300 | **59+** | hsa-miR-19b-1-5p | −28.1 | 0.890 |
| **304+A** | hsa-miR-670 | −26.2 | 1.300 | **59+** | hsa-miR-503-3p | −29.8 | 0.890 |
| **304+A** | hsa-miR-760 | −29.4 | 1.300 | **6+** | hsa-miR-23a-5p | −34.1 | 0.833 |
| **305+** | hsa-miR-27a-5p | −29.4 | 0.547 | **6+** | hsa-miR-27a-5p | −36.3 | 0.833 |
| **305+** | hsa-miR-92a-2-5p | −27.3 | 0.547 | **6+** | hsa-miR-23b-5p | −29.2 | 0.833 |
| **305+** | hsa-miR-149-3p | −30.2 | 0.547 | **6+** | hsa-miR-125b-1-3p | −28.3 | 0.833 |
| **305+** | hsa-miR-99b-3p | −30.2 | 0.547 | **6+** | hsa-miR-135a-3p | −29.4 | 0.833 |
| **305+** | hsa-miR-423-3p | −32.1 | 0.547 | **6+** | hsa-miR-149-3p | −31.2 | 0.833 |
| **305+** | hsa-miR-193b-5p | −34.3 | 0.547 | **6+** | hsa-miR-30c-1-3p | −31.3 | 0.833 |
| **305+** | hsa-miR-593-5p | −35.6 | 0.547 | **6+** | hsa-miR-296-3p | −30.3 | 0.833 |
| **305+** | hsa-miR-596 | −29.9 | 0.547 | **6+** | hsa-miR-193b-5p | −30.1 | 0.833 |
| **305+** | hsa-miR-608 | −31.3 | 0.547 | **6+** | hsa-miR-505-5p | −27.7 | 0.833 |
| **305+** | hsa-miR-661 | −30.9 | 0.547 | **6+** | hsa-miR-637 | −32.7 | 0.833 |
| **305+** | hsa-miR-658 | −32.2 | 0.547 | **6+** | hsa-miR-639 | −29.9 | 0.833 |
| **305+** | hsa-miR-939-5p | −36.2 | 0.547 | **6+** | hsa-miR-762 | −34.1 | 0.833 |
| **305+** | hsa-miR-664b-5p | −32.7 | 0.547 | **6+** | hsa-miR-760 | −31.4 | 0.833 |
| **305+A** | hsa-miR-22-5p | −29.8 | 0.696 | **61+A** | hsa-miR-24-3p | −31.4 | 0.660 |
| **305+A** | hsa-miR-93-3p | −29.7 | 0.696 | **61+A** | hsa-miR-92a-2-5p | −27.2 | 0.660 |
| **305+A** | hsa-miR-27b-5p | −27.1 | 0.696 | **61+A** | hsa-miR-197-5p | −37.3 | 0.660 |
| **305+A** | hsa-miR-296-3p | −30.8 | 0.696 | **61+A** | hsa-miR-198 | −28.8 | 0.660 |
| **305+A** | hsa-miR-331-3p | −30.8 | 0.696 | **61+A** | hsa-miR-125b-1-3p | −28.3 | 0.660 |
| **305+A** | hsa-miR-423-3p | −29.9 | 0.696 | **61+A** | hsa-miR-149-3p | −28.7 | 0.660 |
| **305+A** | hsa-miR-484 | −31.2 | 0.696 | **61+A** | hsa-miR-193a-5p | −28.6 | 0.660 |
| **305+A** | hsa-miR-608 | −31.2 | 0.696 | **61+A** | hsa-miR-323a-5p | −32.8 | 0.660 |
| **305+A** | hsa-miR-612 | −38.5 | 0.696 | **61+A** | hsa-miR-423-5p | −30.0 | 0.660 |
| **305+A** | hsa-miR-657 | −31.7 | 0.696 | **61+A** | hsa-miR-423-3p | −31.5 | 0.660 |
| **305+A** | hsa-miR-670 | −29.0 | 0.696 | **61+A** | hsa-miR-513a-5p | −29.5 | 0.660 |
| **305+A** | hsa-miR-888-3p | −27.4 | 0.696 | **61+A** | hsa-miR-514a-5p | −28.3 | 0.660 |
| **305+A** | hsa-miR-877-3p | −28.4 | 0.696 | **61+A** | hsa-miR-557 | −31.0 | 0.660 |
| **305+A** | hsa-miR-939-3p | −32.3 | 0.696 | **61+A** | hsa-miR-550a-5p | −30.9 | 0.660 |
| **306+** | hsa-miR-31-5p | −27.0 | 0.646 | **61+A** | hsa-miR-602 | −32.5 | 0.660 |
| **306+** | hsa-miR-31-3p | −26.5 | 0.646 | **61+A** | hsa-miR-608 | −32.5 | 0.660 |
| **306+** | hsa-miR-192-5p | −26.3 | 0.646 | **61+A** | hsa-miR-612 | −36.3 | 0.660 |
| **306+** | hsa-miR-345-5p | −31.0 | 0.646 | **61+A** | hsa-miR-661 | −32.8 | 0.660 |
| **306+** | hsa-miR-634 | −26.8 | 0.646 | **61+A** | hsa-miR-762 | −30.1 | 0.660 |
| **306+** | hsa-miR-550a-3-5p | −27.0 | 0.646 | **61+A** | hsa-miR-874 | −33.3 | 0.660 |
| **306+** | hsa-miR-550b-2-5p | −31.9 | 0.646 | **61+A** | hsa-miR-147b | −27.3 | 0.660 |
| **306+** | hsa-miR-664b-3p | −27.0 | 0.646 | **61+A** | hsa-miR-939-5p | −31.0 | 0.660 |
| **31+** | hsa-miR-564 | −27.2 | 2.317 | **61+A** | hsa-miR-550b-2-5p | −27.9 | 0.660 |
| **310+** | hsa-miR-149-3p | −30.1 | 0.578 | **61+A** | hsa-miR-378g | −27.7 | 0.660 |
| **310+** | hsa-miR-765 | −28.3 | 0.578 | **61+A** | hsa-miR-371b-5p | −29.2 | 0.660 |
| **310+** | hsa-miR-939-5p | −33.3 | 0.578 | **63+** | hsa-miR-31-5p | −28.4 | 0.676 |
| **310+** | hsa-miR-544b | −26.0 | 0.578 | **67+** | hsa-miR-185-5p | −27.6 | 0.865 |
| **311+** | hsa-miR-27a-5p | −31.0 | 0.891 | **67+** | hsa-miR-423-5p | −27.2 | 0.865 |
| **311+** | hsa-miR-211-3p | −26.6 | 0.891 | **67+** | hsa-miR-765 | −26.7 | 0.865 |
| **311+** | hsa-let-7g-5p | −25.5 | 0.891 | **67+** | hsa-miR-541-5p | −28.1 | 0.865 |
| **311+** | hsa-miR-138-5p | −27.8 | 0.891 | **67+** | hsa-miR-920 | −27.0 | 0.865 |
| **311+** | hsa-miR-185-3p | −29.1 | 0.891 | **67+** | hsa-miR-939-5p | −31.7 | 0.865 |
| **311+** | hsa-miR-296-3p | −29.3 | 0.891 | **67+** | hsa-miR-940 | −31.6 | 0.865 |
| **311+** | hsa-miR-491-5p | −28.2 | 0.891 | **67+A** | hsa-miR-26b-3p | −27.8 | 0.865 |
| **311+** | hsa-miR-146b-3p | −28.0 | 0.891 | **67+A** | hsa-miR-212-5p | −26.9 | 0.865 |
| **311+** | hsa-miR-518a-3p | −26.6 | 0.891 | **67+A** | hsa-miR-342-5p | −25.8 | 0.865 |
| **311+** | hsa-miR-612 | −30.5 | 0.891 | **67+A** | hsa-miR-632 | −26.9 | 0.865 |
| **311+** | hsa-miR-663a | −35.6 | 0.891 | **68+** | hsa-miR-22-3p | −26.5 | 1.199 |
| **311+** | hsa-miR-744-5p | −29.5 | 0.891 | **68+** | hsa-miR-27a-5p | −28.4 | 1.199 |
| **311+** | hsa-miR-760 | −27.2 | 0.891 | **68+** | hsa-miR-585 | −25.6 | 1.199 |
| **311+** | hsa-miR-939-5p | −31.5 | 0.891 | **70+** | hsa-let-7g-5p | −26.7 | 0.693 |
| **311+** | hsa-miR-940 | −36.5 | 0.891 | **70+** | hsa-miR-27b-3p | −26.1 | 0.693 |
| **311+** | hsa-miR-378b | −27.8 | 0.891 | **70+** | hsa-miR-149-3p | −30.6 | 0.693 |
| **317+** | hsa-miR-574-5p | −27.4 | 1.403 | **70+** | hsa-miR-185-5p | −29.6 | 0.693 |
| **317+** | hsa-miR-760 | −28.4 | 1.403 | **70+** | hsa-miR-302c-5p | −27.7 | 0.693 |
| **319+A** | hsa-miR-129-5p | −27.6 | 1.669 | **70+** | hsa-miR-486-3p | −28.5 | 0.693 |
| **319+A** | hsa-miR-623 | −30.5 | 1.669 | **70+** | hsa-miR-503-3p | −29.3 | 0.693 |
| **319+A** | hsa-miR-629-3p | −29.9 | 1.669 | **70+** | hsa-miR-600 | −31.2 | 0.693 |
| **319+A** | hsa-miR-648 | −26.8 | 1.669 | **70+** | hsa-miR-608 | −29.3 | 0.693 |
| **319+A** | hsa-miR-877-3p | −28.1 | 1.669 | **70+** | hsa-miR-637 | −32.5 | 0.693 |
| **319+A** | hsa-miR-642b-5p | −28.6 | 1.669 | **70+** | hsa-miR-650 | −29.4 | 0.693 |
| **321+A** | hsa-miR-24-1-5p | −26.0 | 0.808 | **70+** | hsa-miR-762 | −33.1 | 0.693 |
| **321+A** | hsa-miR-198 | −30.9 | 0.808 | **73+** | hsa-miR-27a-5p | −28.0 | 1.273 |
| **321+A** | hsa-miR-9-5p | −26.0 | 0.808 | **73+** | hsa-miR-143-5p | −31.1 | 1.273 |
| **321+A** | hsa-miR-149-3p | −33.8 | 0.808 | **73+** | hsa-miR-188-3p | −25.9 | 1.273 |
| **321+A** | hsa-miR-423-5p | −28.6 | 0.808 | **73+** | hsa-miR-106b-3p | −28.7 | 1.273 |
| **321+A** | hsa-miR-512-3p | −27.0 | 0.808 | **73+** | hsa-miR-135b-3p | −27.6 | 1.273 |
| **321+A** | hsa-miR-628-5p | −25.2 | 0.808 | **73+** | hsa-miR-425-3p | −28.6 | 1.273 |
| **321+A** | hsa-miR-766-5p | −30.3 | 0.808 | **73+** | hsa-miR-500a-5p | −28.1 | 1.273 |
| **321+A** | hsa-miR-762 | −30.9 | 0.808 | **73+** | hsa-miR-554 | −26.5 | 1.273 |
| **321+A** | hsa-miR-939-5p | −33.7 | 0.808 | **73+** | hsa-miR-598 | −33.9 | 1.273 |
| **323+** | hsa-miR-15a-3p | −34.4 | 0.642 | **73+** | hsa-miR-637 | −32.1 | 1.273 |
| **323+** | hsa-miR-30b-3p | −27.0 | 0.642 | **73+** | hsa-miR-640 | −26.7 | 1.273 |
| **324+** | hsa-miR-29b-1-5p | −29.1 | 0.497 | **73+** | hsa-miR-892a | −26.2 | 1.273 |
| **324+** | hsa-miR-34a-5p | −26.5 | 0.497 | **73+** | hsa-miR-744-5p | −28.6 | 1.273 |
| **324+** | hsa-miR-143-5p | −28.6 | 0.497 | **73+** | hsa-miR-760 | −28.8 | 1.273 |
| **324+** | hsa-miR-299-3p | −26.7 | 0.497 | **73+** | hsa-miR-941 | −27.3 | 1.273 |
| **324+** | hsa-miR-370 | −30.0 | 0.497 | **73+** | hsa-miR-500b | −24.5 | 1.273 |
| **324+** | hsa-miR-323a-5p | −28.8 | 0.497 | **77+** | hsa-miR-431-5p | −27.4 | 0.739 |
| **324+** | hsa-miR-486-3p | −29.8 | 0.497 | **77+** | hsa-miR-501-5p | −30.3 | 0.739 |
| **324+** | hsa-miR-491-5p | −28.1 | 0.497 | **77+** | hsa-miR-574-3p | −27.7 | 0.739 |
| **324+** | hsa-miR-502-5p | −26.0 | 0.497 | **77+** | hsa-miR-499b-5p | −26.5 | 0.739 |
| **324+** | hsa-miR-574-5p | −30.5 | 0.497 | **77+A** | hsa-miR-575 | −27.5 | 0.739 |
| **324+** | hsa-miR-608 | −32.9 | 0.497 | **77+A** | hsa-miR-920 | −28.1 | 0.739 |
| **324+** | hsa-miR-615-5p | −28.9 | 0.497 | **78+** | hsa-miR-24-3p | −28.4 | 0.677 |
| **324+** | hsa-miR-637 | −32.8 | 0.497 | **78+** | hsa-miR-149-3p | −27.8 | 0.677 |
| **324+** | hsa-miR-659-3p | −27.6 | 0.497 | **78+** | hsa-miR-486-3p | −32.0 | 0.677 |
| **324+A** | hsa-miR-198 | −27.1 | 0.804 | **78+** | hsa-miR-503-3p | −31.5 | 0.677 |
| **324+A** | hsa-let-7i-3p | −27.5 | 0.804 | **78+** | hsa-miR-602 | −30.5 | 0.677 |
| **324+A** | hsa-miR-125a-3p | −29.3 | 0.804 | **78+** | hsa-miR-622 | −27.4 | 0.677 |
| **324+A** | hsa-miR-185-3p | −27.4 | 0.804 | **78+** | hsa-miR-657 | −29.8 | 0.677 |
| **324+A** | hsa-miR-194-3p | −27.7 | 0.804 | **78+** | hsa-miR-744-5p | −32.5 | 0.677 |
| **324+A** | hsa-miR-365b-5p | −27.2 | 0.804 | **78+** | hsa-miR-760 | −28.0 | 0.677 |
| **324+A** | hsa-miR-486-3p | −26.6 | 0.804 | **8+** | hsa-miR-381-3p | −27.1 | 6.657 |
| **324+A** | hsa-miR-491-5p | −29.7 | 0.804 | **8+** | hsa-miR-596 | −33.5 | 6.657 |
| **324+A** | hsa-miR-498 | −29.0 | 0.804 | **84+A** | hsa-miR-660-3p | −26.8 | 1.650 |
| **324+A** | hsa-miR-519e-5p | −26.0 | 0.804 | **84+A** | hsa-miR-642b-5p | −30.2 | 1.650 |
| **324+A** | hsa-miR-504 | −28.1 | 0.804 | **85+A** | hsa-miR-149-3p | −28.6 | 0.921 |
| **324+A** | hsa-miR-508-5p | −32.0 | 0.804 | **85+A** | hsa-miR-188-5p | −28.0 | 0.921 |
| **324+A** | hsa-miR-557 | −28.0 | 0.804 | **85+A** | hsa-miR-188-3p | −26.8 | 0.921 |
| **324+A** | hsa-miR-587 | −27.1 | 0.804 | **85+A** | hsa-miR-485-5p | −28.4 | 0.921 |
| **324+A** | hsa-miR-550a-3p | −26.7 | 0.804 | **85+A** | hsa-miR-516b-3p | −24.9 | 0.921 |
| **324+A** | hsa-miR-602 | −34.6 | 0.804 | **85+A** | hsa-miR-516a-3p | −24.9 | 0.921 |
| **324+A** | hsa-miR-612 | −28.7 | 0.804 | **85+A** | hsa-miR-718 | −28.8 | 0.921 |
| **324+A** | hsa-miR-615-3p | −34.6 | 0.804 | **85+A** | hsa-miR-892c-3p | −26.4 | 0.921 |
| **324+A** | hsa-miR-663a | −34.8 | 0.804 | **88+** | hsa-let-7a-5p | −27.0 | 0.499 |
| **324+A** | hsa-miR-654-5p | −28.2 | 0.804 | **88+** | hsa-let-7b-5p | −29.3 | 0.499 |
| **324+A** | hsa-miR-654-3p | −26.8 | 0.804 | **88+** | hsa-let-7c | −29.9 | 0.499 |
| **324+A** | hsa-miR-762 | −30.3 | 0.804 | **88+** | hsa-let-7d-5p | −27.6 | 0.499 |
| **324+A** | hsa-miR-764 | −30.2 | 0.804 | **88+** | hsa-let-7f-5p | −26.9 | 0.499 |
| **324+A** | hsa-miR-676-3p | −26.3 | 0.804 | **88+** | hsa-miR-92a-1-5p | −29.1 | 0.499 |
| **325+A** | hsa-miR-191-5p | −30.4 | 0.288 | **88+** | hsa-miR-93-3p | −32.0 | 0.499 |
| **325+A** | hsa-miR-424-3p | −31.6 | 0.288 | **88+** | hsa-miR-204-5p | −28.5 | 0.499 |
| **325+A** | hsa-miR-490-5p | −26.3 | 0.288 | **88+** | hsa-miR-211-5p | −29.9 | 0.499 |
| **325+A** | hsa-miR-638 | −31.4 | 0.288 | **88+** | hsa-miR-211-3p | −29.6 | 0.499 |
| **325+A** | hsa-miR-663a | −30.5 | 0.288 | **88+** | hsa-miR-214-5p | −29.2 | 0.499 |
| **326+** | hsa-miR-93-3p | −28.9 | 1.611 | **88+** | hsa-let-7g-5p | −28.7 | 0.499 |
| **326+** | hsa-miR-193a-5p | −28.1 | 1.611 | **88+** | hsa-let-7i-5p | −32.3 | 0.499 |
| **326+** | hsa-miR-493-3p | −30.7 | 1.611 | **88+** | hsa-miR-133a | −28.6 | 0.499 |
| **326+** | hsa-miR-514a-3p | −26.4 | 1.611 | **88+** | hsa-miR-191-3p | −33.4 | 0.499 |
| **326+** | hsa-miR-593-3p | −26.4 | 1.611 | **88+** | hsa-miR-149-3p | −30.5 | 0.499 |
| **326+** | hsa-miR-514b-3p | −27.3 | 1.611 | **88+** | hsa-miR-185-5p | −30.5 | 0.499 |
| **327+A** | hsa-miR-532-3p | −30.7 | 1.768 | **88+** | hsa-miR-370 | −33.2 | 0.499 |
| **327+A** | hsa-miR-612 | −29.7 | 1.768 | **88+** | hsa-miR-328 | −34.1 | 0.499 |
| **327+A** | hsa-miR-627 | −26.3 | 1.768 | **88+** | hsa-miR-133b | −28.6 | 0.499 |
| **327+A** | hsa-miR-449c-5p | −29.7 | 1.768 | **88+** | hsa-miR-346 | −36.8 | 0.499 |
| **327+A** | hsa-miR-888-3p | −29.2 | 1.768 | **88+** | hsa-miR-449a | −28.3 | 0.499 |
| **327+A** | hsa-miR-642b-5p | −27.9 | 1.768 | **88+** | hsa-miR-572 | −28.6 | 0.499 |
| **329+** | hsa-miR-19a-5p | −26.7 | 0.855 | **88+** | hsa-miR-585 | −29.7 | 0.499 |
| **329+** | hsa-miR-24-2-5p | −28.4 | 0.856 | **88+** | hsa-miR-608 | −34.4 | 0.499 |
| **329+** | hsa-miR-30b-3p | −30.0 | 0.857 | **88+** | hsa-miR-612 | −33.4 | 0.499 |
| **329+** | hsa-miR-575 | −30.2 | 0.858 | **88+** | hsa-miR-623 | −29.1 | 0.499 |
| **329+** | hsa-miR-885-3p | −29.6 | 0.859 | **88+** | hsa-miR-629-3p | −30.3 | 0.499 |
| **329+** | hsa-miR-939-5p | −37.0 | 0.860 | **88+** | hsa-miR-636 | −31.3 | 0.499 |
| **33+** | hsa-miR-339-5p | −29.1 | 0.325 | **88+** | hsa-miR-646 | −28.1 | 0.499 |
| **33+** | hsa-miR-18b-3p | −30.1 | 0.325 | **88+** | hsa-miR-449b-5p | −28.3 | 0.499 |
| **33+** | hsa-miR-409-3p | −28.3 | 0.325 | **88+** | hsa-miR-449c-3p | −31.8 | 0.499 |
| **33+** | hsa-miR-491-5p | −28.5 | 0.325 | **88+** | hsa-miR-762 | −36.2 | 0.499 |
| **33+** | hsa-miR-634 | −27.8 | 0.325 | **88+** | hsa-miR-761 | −29.5 | 0.499 |
| **33+** | hsa-miR-636 | −29.2 | 0.325 | **88+** | hsa-miR-765 | −32.3 | 0.499 |
| **33+** | hsa-miR-640 | −27.4 | 0.325 | **88+** | hsa-miR-675-5p | −32.2 | 0.499 |
| **33+** | hsa-miR-647 | −28.1 | 0.325 | **88+** | hsa-miR-675-3p | −28.2 | 0.499 |
| **33+** | hsa-miR-935 | −31.0 | 0.325 | **88+** | hsa-miR-147b | −27.3 | 0.499 |
| **333+A** | hsa-miR-103a-3p | −31.4 | 0.701 | **88+** | hsa-miR-885-3p | −30.4 | 0.499 |
| **333+A** | hsa-miR-107 | −31.3 | 0.701 | **88+** | hsa-miR-877-3p | −28.5 | 0.499 |
| **333+A** | hsa-miR-125a-3p | −28.1 | 0.701 | **88+** | hsa-miR-920 | −28.1 | 0.499 |
| **333+A** | hsa-miR-149-3p | −34.2 | 0.701 | **88+** | hsa-miR-939-5p | −30.3 | 0.499 |
| **333+A** | hsa-miR-342-5p | −27.2 | 0.701 | **88+** | hsa-miR-718 | −34.2 | 0.499 |
| **333+A** | hsa-miR-423-3p | −29.5 | 0.701 | **88+A** | hsa-miR-25-5p | −29.8 | 0.704 |
| **333+A** | hsa-miR-511 | −28.7 | 0.701 | **88+A** | hsa-miR-211-5p | −29.0 | 0.704 |
| **333+A** | hsa-miR-432-5p | −29.2 | 0.701 | **88+A** | hsa-miR-191-3p | −31.2 | 0.704 |
| **333+A** | hsa-miR-552 | −26.5 | 0.701 | **88+A** | hsa-miR-149-5p | −32.0 | 0.704 |
| **333+A** | hsa-miR-659-3p | −29.9 | 0.701 | **88+A** | hsa-miR-149-3p | −34.1 | 0.704 |
| **333+A** | hsa-miR-766-5p | −28.6 | 0.701 | **88+A** | hsa-miR-194-3p | −28.4 | 0.704 |
| **333+A** | hsa-miR-762 | −32.4 | 0.701 | **88+A** | hsa-miR-34c-5p | −29.7 | 0.704 |
| **333+A** | hsa-miR-760 | −27.5 | 0.701 | **88+A** | hsa-miR-296-3p | −28.6 | 0.704 |
| **333+A** | hsa-miR-920 | −27.8 | 0.701 | **88+A** | hsa-miR-449a | −27.5 | 0.704 |
| **333+A** | hsa-miR-664b-5p | −29.5 | 0.701 | **88+A** | hsa-miR-409-3p | −30.0 | 0.704 |
| **334+** | hsa-miR-15a-3p | −32.0 | 1.213 | **88+A** | hsa-miR-146b-3p | −29.1 | 0.704 |
| **334+** | hsa-miR-23a-5p | −30.0 | 1.213 | **88+A** | hsa-miR-636 | −33.9 | 0.704 |
| **334+** | hsa-miR-27a-5p | −26.7 | 1.213 | **88+A** | hsa-miR-639 | −35.4 | 0.704 |
| **334+** | hsa-miR-34a-5p | −28.0 | 1.213 | **88+A** | hsa-miR-642a-5p | −30.2 | 0.704 |
| **334+** | hsa-miR-200b-5p | −27.6 | 1.213 | **88+A** | hsa-miR-661 | −32.8 | 0.704 |
| **334+** | hsa-miR-23b-5p | −27.2 | 1.213 | **88+A** | hsa-miR-663a | −33.9 | 0.704 |
| **334+** | hsa-miR-125b-1-3p | −29.6 | 1.213 | **88+A** | hsa-miR-449b-5p | −28.4 | 0.704 |
| **334+** | hsa-miR-185-3p | −28.5 | 1.213 | **88+A** | hsa-miR-658 | −35.9 | 0.704 |
| **334+** | hsa-miR-194-3p | −34.9 | 1.213 | **88+A** | hsa-miR-744-5p | −29.6 | 0.704 |
| **334+** | hsa-miR-432-5p | −30.6 | 1.213 | **88+A** | hsa-miR-877-3p | −33.2 | 0.704 |
| **334+** | hsa-miR-652-5p | −31.5 | 1.213 | **88+A** | hsa-miR-920 | −30.0 | 0.704 |
| **334+** | hsa-miR-671-5p | −34.6 | 1.213 | **88+A** | hsa-miR-933 | −32.7 | 0.704 |
| **334+** | hsa-miR-449c-5p | −28.0 | 1.213 | **88+A** | hsa-miR-939-5p | −33.0 | 0.704 |
| **334+** | hsa-miR-762 | −35.3 | 1.213 | **88+A** | hsa-miR-942 | −28.6 | 0.704 |
| **334+** | hsa-miR-670 | −25.9 | 1.213 | **88+A** | hsa-miR-718 | −29.0 | 0.704 |
| **334+** | hsa-miR-298 | −27.9 | 1.213 | **9+A** | hsa-miR-204-5p | −25.9 | 1.431 |
| **334+** | hsa-miR-873-5p | −27.0 | 1.213 | **9+A** | hsa-miR-211-5p | −26.6 | 1.431 |
| **334+** | hsa-miR-760 | −26.8 | 1.213 | **9+A** | hsa-miR-212-5p | −30.0 | 1.431 |
| **334+A** | hsa-miR-197-5p | −28.8 | 1.213 | **9+A** | hsa-miR-578 | −26.5 | 1.431 |
| **334+A** | hsa-miR-10b-5p | −26.7 | 1.213 | **9+A** | hsa-miR-593-3p | −29.3 | 1.431 |
| **334+A** | hsa-miR-296-3p | −31.8 | 1.213 | **9+A** | hsa-miR-659-3p | −31.5 | 1.431 |
| **334+A** | hsa-miR-365b-5p | −27.9 | 1.213 | **9+A** | hsa-miR-454-3p | −29.0 | 1.431 |
| **334+A** | hsa-miR-146b-3p | −28.3 | 1.213 | **9+A** | hsa-miR-764 | −27.5 | 1.431 |
| **334+A** | hsa-miR-493-5p | −29.6 | 1.213 | **9+A** | hsa-miR-877-3p | −32.4 | 1.431 |
| **334+A** | hsa-miR-596 | −27.8 | 1.213 | **9+A** | hsa-miR-642b-5p | −27.8 | 1.431 |
| **334+A** | hsa-miR-612 | −34.1 | 1.213 | **92+A** | hsa-miR-185-3p | −31.1 | 0.853 |
| **334+A** | hsa-miR-615-5p | −29.1 | 1.213 | **92+A** | hsa-miR-106b-3p | −29.6 | 0.853 |
| **334+A** | hsa-miR-638 | −32.6 | 1.213 | **92+A** | hsa-miR-326 | −30.5 | 0.853 |
| **334+A** | hsa-miR-646 | −26.4 | 1.213 | **92+A** | hsa-miR-602 | −29.7 | 0.853 |
| **334+A** | hsa-miR-650 | −30.5 | 1.213 | **92+A** | hsa-miR-663a | −36.0 | 0.853 |
| **334+A** | hsa-miR-661 | −29.1 | 1.213 | **92+A** | hsa-miR-658 | −31.2 | 0.853 |
| **334+A** | hsa-miR-449c-5p | −28.0 | 1.213 | **92+A** | hsa-miR-876-3p | −26.4 | 0.853 |
| **334+A** | hsa-miR-885-3p | −30.2 | 1.213 | **92+A** | hsa-miR-103b | −30.2 | 0.853 |
| **334+A** | hsa-miR-940 | −28.5 | 1.213 | **96+** | hsa-miR-631 | −27.7 | 0.426 |
| **334+A** | hsa-miR-663b | −32.3 | 1.213 | **97+A** | hsa-miR-103a-2-5p | −28.5 | 0.644 |
| **335+** | hsa-miR-31-5p | −30.1 | 1.347 | **97+A** | hsa-miR-125a-3p | −29.9 | 0.644 |
| **335+** | hsa-miR-221-3p | −29.3 | 1.347 | **97+A** | hsa-miR-194-3p | −33.8 | 0.644 |
| **335+** | hsa-miR-222-3p | −30.2 | 1.347 | **97+A** | hsa-miR-557 | −30.4 | 0.644 |
| **335+** | hsa-miR-365b-5p | −27.3 | 1.347 | **97+A** | hsa-miR-298 | −31.6 | 0.644 |

**Table S3.** T-UCRs/miRNAs predicted single and multiple binding sites.

| **T-UCRs without  miRNA Binding** | **T-UCRs with  Binding Site for  Single miRNAs** | **T-UCRs with Binding  Site for Multiple  miRNAs (from 2 to 70)** | **miRNA with  Binding Site for  Single T-UCRs** | **miRNA with Binding  Site for Multiple  T-UCRs (from 2 to 47)** |
| --- | --- | --- | --- | --- |
| 10+ | 102+ | 102+A | hsa-let-7d-3p | hsa-let-7a-5p |
| 128+ | 106+A | 12+ | hsa-let-7f-1-3p | hsa-let-7e-5p |
| 173+A | 112+A | 121+A | hsa-miR-105-5p | hsa-let-7f-5p |
| 206+A | 115+A | 141+ | hsa-miR-106a-5p | hsa-miR-105-3p |
| 221+ | 125+A | 146+ | hsa-miR-10b-5p | hsa-miR-106a-3p |
| 225+A | 133+A | 166+ | hsa-miR-122-5p | hsa-miR-124-5p |
| 230+ | 138+ | 191+A | hsa-miR-127-3p | hsa-miR-125b-5p |
| 247+A | 150+ | 229+A | hsa-miR-129-1-3p | hsa-miR-1264 |
| 30+ | 156+A | 244+ | hsa-miR-129-2-3p | hsa-miR-130b-5p |
| 302+A | 172+A | 253+ | hsa-miR-130a-5p | hsa-miR-132-3p |
| 322+ | 188+ | 28+ | hsa-miR-132-5p | hsa-miR-135b-3p |
| 322+A | 208+ | 291+ | hsa-miR-134 | hsa-miR-136-3p |
| 325+ | 208+A | 317+ | hsa-miR-135a-5p | hsa-miR-139-5p |
| 34+A | 227+A | 323+ | hsa-miR-135b-5p | hsa-miR-140-5p |
| 405+A | 234+ | 352+ | hsa-miR-138-1-3p | hsa-miR-147a |
| 432+A | 247+ | 359+ | hsa-miR-140-3p | hsa-miR-148b-3p |
| 89+ | 289+ | 360+ | hsa-miR-141-5p | hsa-miR-15b-5p |
|  | 289+A | 446+ | hsa-miR-145-3p | hsa-miR-16-1-3p |
|  | 302+ | 450+A | hsa-miR-146a-3p | hsa-miR-181c-3p |
|  | 31+ | 471+A | hsa-miR-146a-5p | hsa-miR-192-5p |
|  | 359+A | 59+ | hsa-miR-146b-5p | hsa-miR-19a-5p |
|  | 385+A | 77+A | hsa-miR-148a-3p | hsa-miR-212-3p |
|  | 399+A | 8+ | hsa-miR-148a-5p | hsa-miR-216a-3p |
|  | 4+ | 84+A | hsa-miR-148b-5p | hsa-miR-216a-5p |
|  | 410+ | 126+A | hsa-miR-150-5p | hsa-miR-219-1-3p |
|  | 432+ | 137+A | hsa-miR-151a-3p | hsa-miR-219-5p |
|  | 445+A | 139+ | hsa-miR-151a-5p | hsa-miR-22-5p |
|  | 466+A | 142+A | hsa-miR-151b | hsa-miR-222-5p |
|  | 472+A | 163+ | hsa-miR-152 | hsa-miR-23a-3p |
|  | 482+A | 168+A | hsa-miR-16-5p | hsa-miR-24-2-5p |
|  | 5+A | 17+A | hsa-miR-17-5p | hsa-miR-28-3p |
|  | 63+ | 21+A | hsa-miR-181a-5p | hsa-miR-28-5p |
|  | 96+ | 239+A | hsa-miR-182-3p | hsa-miR-29b-2-5p |
|  |  | 252+A | hsa-miR-184 | hsa-miR-31-3p |
|  |  | 257+ | hsa-miR-185-3p | hsa-miR-323a-3p |
|  |  | 274+A | hsa-miR-191-5p | hsa-miR-339-3p |
|  |  | 287+A | hsa-miR-193a-3p | hsa-miR-371a-5p |
|  |  | 288+A | hsa-miR-196b-3p | hsa-miR-371b-5p |
|  |  | 349+ | hsa-miR-197-3p | hsa-miR-373-3p |
|  |  | 399+ | hsa-miR-199a-3p | hsa-miR-383 |
|  |  | 403+ | hsa-miR-199b-3p | hsa-miR-409-5p |
|  |  | 403+A | hsa-miR-199b-5p | hsa-miR-424-3p |
|  |  | 435+ | hsa-miR-200a-5p | hsa-miR-424-5p |
|  |  | 448+A | hsa-miR-200b-5p | hsa-miR-431-3p |
|  |  | 451+ | hsa-miR-20a-3p | hsa-miR-450b-3p |
|  |  | 68+ | hsa-miR-20a-5p | hsa-miR-483-5p |
|  |  | 10+ | hsa-miR-20b-5p | hsa-miR-485-3p |
|  |  | 12+A | hsa-miR-218-5p | hsa-miR-498 |
|  |  | 126+ | hsa-miR-219-2-3p | hsa-miR-499b-5p |
|  |  | 140+A | hsa-miR-221-5p | hsa-miR-500b |
|  |  | 19+ | hsa-miR-223-3p | hsa-miR-508-3p |
|  |  | 213+A | hsa-miR-223-5p | hsa-miR-509-3p |
|  |  | 220+ | hsa-miR-224-5p | hsa-miR-510 |
|  |  | 223+A | hsa-miR-24-1-5p | hsa-miR-513b |
|  |  | 249+ | hsa-miR-26a-1-3p | hsa-miR-514a-3p |
|  |  | 282+A | hsa-miR-26a-5p | hsa-miR-514a-5p |
|  |  | 310+ | hsa-miR-299-3p | hsa-miR-515-3p |
|  |  | 350+ | hsa-miR-29a-5p | hsa-miR-516a-5p |
|  |  | 363+A | hsa-miR-29b-3p | hsa-miR-516b-5p |
|  |  | 372+A | hsa-miR-29c-5p | hsa-miR-518e-5p |
|  |  | 377+ | hsa-miR-301a-5p | hsa-miR-518f-5p |
|  |  | 402+ | hsa-miR-302a-3p | hsa-miR-519a-5p |
|  |  | 402+A | hsa-miR-302c-5p | hsa-miR-519b-3p |
|  |  | 404+ | hsa-miR-302d-3p | hsa-miR-519b-5p |
|  |  | 420+ | hsa-miR-302d-5p | hsa-miR-519c-5p |
|  |  | 67+A | hsa-miR-302e | hsa-miR-519e-3p |
|  |  | 77+ | hsa-miR-30a-3p | hsa-miR-520b |
|  |  | 134+ | hsa-miR-30d-3p | hsa-miR-520c-3p |
|  |  | 136+ | hsa-miR-30d-5p | hsa-miR-520d-3p |
|  |  | 153+A | hsa-miR-320e | hsa-miR-520f |
|  |  | 182+ | hsa-miR-323b-3p | hsa-miR-523-3p |
|  |  | 241+A | hsa-miR-325 | hsa-miR-526a |
|  |  | 275+A | hsa-miR-337-3p | hsa-miR-548v |
|  |  | 325+A | hsa-miR-337-5p | hsa-miR-550a-3p |
|  |  | 358+ | hsa-miR-338-3p | hsa-miR-555 |
|  |  | 374+A | hsa-miR-34a-3p | hsa-miR-563 |
|  |  | 389+ | hsa-miR-34c-3p | hsa-miR-574-3p |
|  |  | 390+ | hsa-miR-361-5p | hsa-miR-584-5p |
|  |  | 419+ | hsa-miR-363-3p | hsa-miR-588 |
|  |  | 43+ | hsa-miR-367-3p | hsa-miR-598 |
|  |  | 443+A | hsa-miR-371a-3p | hsa-miR-609 |
|  |  | 462+A | hsa-miR-372 | hsa-miR-617 |
|  |  | 97+A | hsa-miR-374b-3p | hsa-miR-626 |
|  |  | 159+A | hsa-miR-377-3p | hsa-miR-631 |
|  |  | 170+A | hsa-miR-378c | hsa-miR-635 |
|  |  | 177+A | hsa-miR-378j | hsa-miR-659-5p |
|  |  | 179+ | hsa-miR-380-5p | hsa-miR-662 |
|  |  | 18+ | hsa-miR-381-3p | hsa-miR-664a-5p |
|  |  | 180+ | hsa-miR-421 | hsa-miR-676-3p |
|  |  | 181+ | hsa-miR-422a | hsa-miR-761 |
|  |  | 189+ | hsa-miR-432-3p | hsa-miR-873-3p |
|  |  | 192+A | hsa-miR-450a-3p | hsa-miR-873-5p |
|  |  | 265+A | hsa-miR-450a-5p | hsa-miR-892b |
|  |  | 276+ | hsa-miR-452-3p | hsa-miR-892c-5p |
|  |  | 298+ | hsa-miR-454-3p | hsa-miR-936 |
|  |  | 3+ | hsa-miR-455-3p | hsa-miR-941 |
|  |  | 319+A | hsa-miR-455-5p | hsa-let-7i-5p |
|  |  | 326+ | hsa-miR-487a | hsa-miR-125a-5p |
|  |  | 327+A | hsa-miR-489 | hsa-miR-133a |
|  |  | 329+ | hsa-miR-490-3p | hsa-miR-133b |
|  |  | 356+ | hsa-miR-492 | hsa-miR-139-3p |
|  |  | 369+ | hsa-miR-497-3p | hsa-miR-15a-5p |
|  |  | 412+A | hsa-miR-499a-3p | hsa-miR-17-3p |
|  |  | 449+A | hsa-miR-499b-3p | hsa-miR-181a-2-3p |
|  |  | 185+ | hsa-miR-500a-5p | hsa-miR-187-3p |
|  |  | 185+A | hsa-miR-501-3p | hsa-miR-193b-3p |
|  |  | 261+ | hsa-miR-505-3p | hsa-miR-195-5p |
|  |  | 268+ | hsa-miR-506-3p | hsa-miR-196a-5p |
|  |  | 36+A | hsa-miR-508-5p | hsa-miR-196b-5p |
|  |  | 371+A | hsa-miR-509-3-5p | hsa-miR-202-3p |
|  |  | 440+A | hsa-miR-513c-5p | hsa-miR-20b-3p |
|  |  | 476+A | hsa-miR-514b-5p | hsa-miR-217 |
|  |  | 67+ | hsa-miR-515-5p | hsa-miR-218-1-3p |
|  |  | 143+A | hsa-miR-516a-3p | hsa-miR-218-2-3p |
|  |  | 160+ | hsa-miR-516b-3p | hsa-miR-22-3p |
|  |  | 170+ | hsa-miR-517a-3p | hsa-miR-27a-3p |
|  |  | 196+ | hsa-miR-517b-3p | hsa-miR-30c-2-3p |
|  |  | 283+A | hsa-miR-517c-3p | hsa-miR-33a-5p |
|  |  | 306+ | hsa-miR-518a-3p | hsa-miR-33b-5p |
|  |  | 342+ | hsa-miR-518a-5p | hsa-miR-361-3p |
|  |  | 366+ | hsa-miR-518c-5p | hsa-miR-367-5p |
|  |  | 381+ | hsa-miR-518d-5p | hsa-miR-375 |
|  |  | 392+A | hsa-miR-518d-5p | hsa-miR-376a-2-5p |
|  |  | 396+ | hsa-miR-518e-3p | hsa-miR-377-5p |
|  |  | 456+A | hsa-miR-518e-5p | hsa-miR-378h |
|  |  | 476+ | hsa-miR-518f-5p | hsa-miR-381-5p |
|  |  | 85+A | hsa-miR-519a-5p | hsa-miR-409-3p |
|  |  | 92+A | hsa-miR-519b-5p | hsa-miR-486-5p |
|  |  | 180+A | hsa-miR-519c-3p | hsa-miR-493-3p |
|  |  | 218+ | hsa-miR-519c-5p | hsa-miR-493-5p |
|  |  | 24+A | hsa-miR-519e-5p | hsa-miR-500a-3p |
|  |  | 276+A | hsa-miR-520a-3p | hsa-miR-501-5p |
|  |  | 33+ | hsa-miR-520a-5p | hsa-miR-502-3p |
|  |  | 345+A | hsa-miR-520a-5p | hsa-miR-508-5p |
|  |  | 377+A | hsa-miR-520c-5p | hsa-miR-509-5p |
|  |  | 457+ | hsa-miR-520c-5p | hsa-miR-513a-5p |
|  |  | 78+ | hsa-miR-520e | hsa-miR-516a-3p |
|  |  | 190+A | hsa-miR-521 | hsa-miR-516b-3p |
|  |  | 201+ | hsa-miR-522-3p | hsa-miR-517-5p |
|  |  | 215+A | hsa-miR-526b-3p | hsa-miR-518c-5p |
|  |  | 217+A | hsa-miR-527 | hsa-miR-522-5p |
|  |  | 274+ | hsa-miR-539-3p | hsa-miR-523-5p |
|  |  | 295+A | hsa-miR-539-5p | hsa-miR-532-3p |
|  |  | 321+A | hsa-miR-542-5p | hsa-miR-532-5p |
|  |  | 343+A | hsa-miR-548ag | hsa-miR-541-5p |
|  |  | 417+ | hsa-miR-548ai | hsa-miR-548au-3p |
|  |  | 450+ | hsa-miR-548aq-5p | hsa-miR-551a |
|  |  | 454+ | hsa-miR-548at-5p | hsa-miR-566 |
|  |  | 468+ | hsa-miR-548aw | hsa-miR-600 |
|  |  | 9+A | hsa-miR-548b-3p | hsa-miR-613 |
|  |  | 16+ | hsa-miR-548p | hsa-miR-616-3p |
|  |  | 238+ | hsa-miR-548s | hsa-miR-619 |
|  |  | 278+ | hsa-miR-548w | hsa-miR-625-5p |
|  |  | 281+ | hsa-miR-551b-3p | hsa-miR-627 |
|  |  | 340+ | hsa-miR-552 | hsa-miR-634 |
|  |  | 372+ | hsa-miR-558 | hsa-miR-640 |
|  |  | 382+A | hsa-miR-562 | hsa-miR-644a |
|  |  | 452+ | hsa-miR-567 | hsa-miR-664b-3p |
|  |  | 465+ | hsa-miR-569 | hsa-miR-758-5p |
|  |  | 469+A | hsa-miR-570-5p | hsa-miR-766-3p |
|  |  | 48+A | hsa-miR-571 | hsa-miR-770-5p |
|  |  | 1+ | hsa-miR-573 | hsa-miR-888-3p |
|  |  | 149+ | hsa-miR-587 | hsa-miR-892a |
|  |  | 151+A | hsa-miR-597 | hsa-miR-892c-3p |
|  |  | 248+A | hsa-miR-601 | hsa-miR-935 |
|  |  | 263+A | hsa-miR-604 | hsa-miR-937-3p |
|  |  | 266+A | hsa-miR-611 | hsa-miR-96-5p |
|  |  | 296+ | hsa-miR-620 | hsa-miR-99b-3p |
|  |  | 304+A | hsa-miR-624-3p | hsa-let-7c |
|  |  | 436+ | hsa-miR-628-5p | hsa-let-7e-3p |
|  |  | 458+A | hsa-miR-642a-3p | hsa-let-7g-3p |
|  |  | 57+ | hsa-miR-642b-3p | hsa-miR-127-5p |
|  |  | 70+ | hsa-miR-652-5p | hsa-miR-128 |
|  |  | 238+A | hsa-miR-654-3p | hsa-miR-147b |
|  |  | 305+ | hsa-miR-660-5p | hsa-miR-18b-3p |
|  |  | 56+ | hsa-miR-708-3p | hsa-miR-19b-2-5p |
|  |  | 200+ | hsa-miR-875-3p | hsa-miR-214-3p |
|  |  | 305+A | hsa-miR-876-3p | hsa-miR-24-3p |
|  |  | 324+ | hsa-miR-885-5p | hsa-miR-27b-3p |
|  |  | 354+A | hsa-miR-887 | hsa-miR-29b-1-5p |
|  |  | 362+A | hsa-miR-890 | hsa-miR-320d |
|  |  | 425+A | hsa-miR-93-5p | hsa-miR-331-5p |
|  |  | 427+ | hsa-miR-934 | hsa-miR-345-3p |
|  |  | 479+ | hsa-miR-938 | hsa-miR-34b-5p |
|  |  | 6+ | hsa-miR-939-5p | hsa-miR-363-5p |
|  |  | 122+ | hsa-miR-942 | hsa-miR-378a-5p |
|  |  | 269+A | hsa-miR-943 | hsa-miR-378b |
|  |  | 292+ | hsa-miR-96-3p | hsa-miR-378e |
|  |  | 333+A | hsa-miR-99a-3p | hsa-miR-378g |
|  |  | 397+A |  | hsa-miR-425-3p |
|  |  | 416+ |  | hsa-miR-433 |
|  |  | 453+A |  | hsa-miR-483-3p |
|  |  | 122+A |  | hsa-miR-488-5p |
|  |  | 149+A |  | hsa-miR-490-5p |
|  |  | 20+ |  | hsa-miR-497-5p |
|  |  | 311+ |  | hsa-miR-504 |
|  |  | 73+ |  | hsa-miR-512-5p |
|  |  | 158+A |  | hsa-miR-525-5p |
|  |  | 162+A |  | hsa-miR-526b-5p |
|  |  | 246+ |  | hsa-miR-544b |
|  |  | 269+ |  | hsa-miR-548q |
|  |  | 283+ |  | hsa-miR-578 |
|  |  | 334+A |  | hsa-miR-614 |
|  |  | 335+ |  | hsa-miR-645 |
|  |  | 343+ |  | hsa-miR-648 |
|  |  | 111+ |  | hsa-miR-654-5p |
|  |  | 195+ |  | hsa-miR-660-3p |
|  |  | 24+ |  | hsa-miR-708-5p |
|  |  | 334+ |  | hsa-miR-744-3p |
|  |  | 378+A |  | hsa-miR-764 |
|  |  | 162+ |  | hsa-miR-767-3p |
|  |  | 458+ |  | hsa-miR-9-5p |
|  |  | 47+ |  | hsa-miR-921 |
|  |  | 157+A |  | hsa-miR-92b-3p |
|  |  | 421+ |  | hsa-miR-933 |
|  |  | 422+ |  | hsa-let-7g-5p |
|  |  | 44+ |  | hsa-miR-106b-3p |
|  |  | 54+ |  | hsa-miR-135a-3p |
|  |  | 145+A |  | hsa-miR-145-5p |
|  |  | 393+ |  | hsa-miR-187-5p |
|  |  | 129+ |  | hsa-miR-188-3p |
|  |  | 144+ |  | hsa-miR-18a-3p |
|  |  | 346+ |  | hsa-miR-204-5p |
|  |  | 324+A |  | hsa-miR-210 |
|  |  | 14+ |  | hsa-miR-221-3p |
|  |  | 61+A |  | hsa-miR-25-3p |
|  |  | 88+A |  | hsa-miR-25-5p |
|  |  | 344+A |  | hsa-miR-26b-3p |
|  |  | 414+ |  | hsa-miR-27b-5p |
|  |  | 346+A |  | hsa-miR-30b-3p |
|  |  | 477+A |  | hsa-miR-30c-1-3p |
|  |  | 183+ |  | hsa-miR-330-3p |
|  |  | 285+ |  | hsa-miR-345-5p |
|  |  | 408+A |  | hsa-miR-34c-5p |
|  |  | 44+A |  | hsa-miR-449b-5p |
|  |  | 388+A |  | hsa-miR-449c-3p |
|  |  | 473+A |  | hsa-miR-485-5p |
|  |  | 388+ |  | hsa-miR-514b-3p |
|  |  | 88+ |  | hsa-miR-550b-2-5p |
|  |  | 398+ |  | hsa-miR-610 |
|  |  | 398+A |  | hsa-miR-629-3p |
|  |  | 13+A |  | hsa-miR-665 |
|  |  | 299+A |  | hsa-miR-675-3p |
|  |  |  |  | hsa-miR-942 |
|  |  |  |  | hsa-let-7a-2-3p |
|  |  |  |  | hsa-let-7b-5p |
|  |  |  |  | hsa-let-7d-5p |
|  |  |  |  | hsa-miR-103a-3p |
|  |  |  |  | hsa-miR-103b |
|  |  |  |  | hsa-miR-129-5p |
|  |  |  |  | hsa-miR-188-5p |
|  |  |  |  | hsa-miR-222-3p |
|  |  |  |  | hsa-miR-320c |
|  |  |  |  | hsa-miR-324-5p |
|  |  |  |  | hsa-miR-449a |
|  |  |  |  | hsa-miR-503-5p |
|  |  |  |  | hsa-miR-505-5p |
|  |  |  |  | hsa-miR-512-3p |
|  |  |  |  | hsa-miR-550a-3-5p |
|  |  |  |  | hsa-miR-554 |
|  |  |  |  | hsa-miR-585 |
|  |  |  |  | hsa-miR-621 |
|  |  |  |  | hsa-miR-647 |
|  |  |  |  | hsa-miR-664b-5p |
|  |  |  |  | hsa-miR-668 |
|  |  |  |  | hsa-miR-670 |
|  |  |  |  | hsa-miR-769-5p |
|  |  |  |  | hsa-miR-92a-3p |
|  |  |  |  | hsa-miR-92b-5p |
|  |  |  |  | hsa-miR-138-5p |
|  |  |  |  | hsa-miR-193a-5p |
|  |  |  |  | hsa-miR-194-3p |
|  |  |  |  | hsa-miR-198 |
|  |  |  |  | hsa-miR-19b-1-5p |
|  |  |  |  | hsa-miR-212-5p |
|  |  |  |  | hsa-miR-296-5p |
|  |  |  |  | hsa-miR-298 |
|  |  |  |  | hsa-miR-412 |
|  |  |  |  | hsa-miR-423-5p |
|  |  |  |  | hsa-miR-484 |
|  |  |  |  | hsa-miR-502-5p |
|  |  |  |  | hsa-miR-503-3p |
|  |  |  |  | hsa-miR-622 |
|  |  |  |  | hsa-miR-642a-5p |
|  |  |  |  | hsa-miR-671-3p |
|  |  |  |  | hsa-miR-766-5p |
|  |  |  |  | hsa-miR-92a-1-5p |
|  |  |  |  | hsa-miR-92a-2-5p |
|  |  |  |  | hsa-miR-107 |
|  |  |  |  | hsa-miR-150-3p |
|  |  |  |  | hsa-miR-185-5p |
|  |  |  |  | hsa-miR-204-3p |
|  |  |  |  | hsa-miR-23b-5p |
|  |  |  |  | hsa-miR-323b-5p |
|  |  |  |  | hsa-miR-33b-3p |
|  |  |  |  | hsa-miR-432-5p |
|  |  |  |  | hsa-miR-511 |
|  |  |  |  | hsa-miR-541-3p |
|  |  |  |  | hsa-miR-564 |
|  |  |  |  | hsa-miR-574-5p |
|  |  |  |  | hsa-miR-584-3p |
|  |  |  |  | hsa-miR-615-3p |
|  |  |  |  | hsa-miR-642b-5p |
|  |  |  |  | hsa-miR-939-3p |
|  |  |  |  | hsa-miR-103a-2-5p |
|  |  |  |  | hsa-miR-191-3p |
|  |  |  |  | hsa-miR-320a |
|  |  |  |  | hsa-miR-320b |
|  |  |  |  | hsa-miR-342-5p |
|  |  |  |  | hsa-miR-431-5p |
|  |  |  |  | hsa-miR-550a-5p |
|  |  |  |  | hsa-miR-593-5p |
|  |  |  |  | hsa-miR-602 |
|  |  |  |  | hsa-miR-632 |
|  |  |  |  | hsa-miR-646 |
|  |  |  |  | hsa-miR-765 |
|  |  |  |  | hsa-miR-767-5p |
|  |  |  |  | hsa-miR-769-3p |
|  |  |  |  | hsa-miR-877-5p |
|  |  |  |  | hsa-miR-93-3p |
|  |  |  |  | hsa-miR-149-5p |
|  |  |  |  | hsa-miR-211-5p |
|  |  |  |  | hsa-miR-214-5p |
|  |  |  |  | hsa-miR-31-5p |
|  |  |  |  | hsa-miR-324-3p |
|  |  |  |  | hsa-miR-330-5p |
|  |  |  |  | hsa-miR-557 |
|  |  |  |  | hsa-miR-657 |
|  |  |  |  | hsa-miR-874 |
|  |  |  |  | hsa-miR-937-5p |
|  |  |  |  | hsa-miR-940 |
|  |  |  |  | hsa-let-7i-3p |
|  |  |  |  | hsa-miR-125b-1-3p |
|  |  |  |  | hsa-miR-15a-3p |
|  |  |  |  | hsa-miR-23a-5p |
|  |  |  |  | hsa-miR-323a-5p |
|  |  |  |  | hsa-miR-331-3p |
|  |  |  |  | hsa-miR-339-5p |
|  |  |  |  | hsa-miR-491-5p |
|  |  |  |  | hsa-miR-575 |
|  |  |  |  | hsa-miR-596 |
|  |  |  |  | hsa-miR-328 |
|  |  |  |  | hsa-miR-593-3p |
|  |  |  |  | hsa-miR-718 |
|  |  |  |  | hsa-miR-877-3p |
|  |  |  |  | hsa-miR-486-3p |
|  |  |  |  | hsa-miR-675-5p |
|  |  |  |  | hsa-miR-125a-3p |
|  |  |  |  | hsa-miR-326 |
|  |  |  |  | hsa-miR-34a-5p |
|  |  |  |  | hsa-miR-365a-5p |
|  |  |  |  | hsa-miR-193b-5p |
|  |  |  |  | hsa-miR-346 |
|  |  |  |  | hsa-miR-449c-5p |
|  |  |  |  | hsa-miR-572 |
|  |  |  |  | hsa-miR-639 |
|  |  |  |  | hsa-miR-659-3p |
|  |  |  |  | hsa-miR-636 |
|  |  |  |  | hsa-miR-658 |
|  |  |  |  | hsa-miR-920 |
|  |  |  |  | hsa-miR-146b-3p |
|  |  |  |  | hsa-miR-650 |
|  |  |  |  | hsa-miR-671-5p |
|  |  |  |  | hsa-miR-143-5p |
|  |  |  |  | hsa-miR-365b-5p |
|  |  |  |  | hsa-miR-423-3p |
|  |  |  |  | hsa-miR-615-5p |
|  |  |  |  | hsa-miR-211-3p |
|  |  |  |  | hsa-miR-370 |
|  |  |  |  | hsa-miR-661 |
|  |  |  |  | hsa-miR-296-3p |
|  |  |  |  | hsa-miR-885-3p |
|  |  |  |  | hsa-miR-623 |
|  |  |  |  | hsa-miR-197-5p |
|  |  |  |  | hsa-miR-637 |
|  |  |  |  | hsa-miR-744-5p |
|  |  |  |  | hsa-miR-663b |
|  |  |  |  | hsa-miR-612 |
|  |  |  |  | hsa-miR-663a |
|  |  |  |  | hsa-miR-760 |
|  |  |  |  | hsa-miR-27a-5p |
|  |  |  |  | hsa-miR-638 |
|  |  |  |  | hsa-miR-185-3p |
|  |  |  |  | hsa-miR-608 |
|  |  |  |  | hsa-miR-149-3p |
|  |  |  |  | hsa-miR-762 |
|  |  |  |  | hsa-miR-939-Sp |

**Table S4.** Clinical characteristics of BlCa patients *.

| **Features** | | | **Clinical Stage** | | | | **Pathological Grade** | | |
| --- | --- | --- | --- | --- | --- | --- | --- | --- | --- |
|  | **Age** | **Sex (Male/Female)** | **Ta-T0** | **T1** | **T2** | **T3-T4** | **G1** | **G2** | **G3** |
| BlCa (N = 24) | 64.5 ± 13.8 | 19/5 | 12 | 3 | 3 | 6 | 4 | 8 | 12 |
| NBE (N = 17) | 61.9 ± 7.1 | 17/0 | / | / | / | / | / | / | / |

* The median follow-up duration for these patients was 27.82 months. All patients were classified according to the 1997 UICC TNM classification for the stage and OMS 2004 for the grade.
